# Donor–acceptor type co-crystals of arylthio-substituted tetrathiafulvalenes and fullerenes

**DOI:** 10.3762/bjoc.11.117

**Published:** 2015-06-19

**Authors:** Xiaofeng Lu, Jibin Sun, Shangxi Zhang, Longfei Ma, Lei Liu, Hui Qi, Yongliang Shao, Xiangfeng Shao

**Affiliations:** 1 State Key Laboratory of Applied Organic Chemistry, Lanzhou University, Tianshui Southern Road 222, Lanzhou 730000, Gansu Province, P. R. China

**Keywords:** arylthio-substituted tetrathiafulvalene, co-crystal, donor–acceptor system, fullerene

## Abstract

A series of donor–acceptor type co-crystals of fullerene (as the acceptor) and arylthio-substituted tetrathiafulvalene derivatives (Ar-S-TTF, as the donor) were prepared and their structural features were thoroughly investigated. The formation of co-crystals relies on the flexibility of Ar-S-TTF and the size matches between Ar-S-TTF and fullerene. Regarding their compositions, the studied co-crystals can be divided into two types, where types I and II have donor:acceptor ratios of 1:1 and 1:2, respectively. Multiple intermolecular interactions are observed between the donor and acceptor, which act to stabilize the structures of the resulting co-crystals. In the type I co-crystals, the fullerene molecule is surrounded by four Ar-S-TTF molecules, that is, two Ar-S-TTF molecules form a sandwich structure with one fullerene molecule and the other two Ar-S-TTF molecules interact with the fullerene molecule along their lateral axes. In the type II co-crystals, one fullerene molecule has the donor–acceptor mode similar to that in type I, whereas the other fullerene molecule is substantially surrounded by the aryl groups on Ar-S-TTF molecules and the solvent molecules.

## Introduction

Tetrathiafulvalene (TTF) [1–3] and its derivatives have attracted significant interest for decades. This is because this unique heterocycle system has provided most of the organic conductors possessing diverse electronic ground states [4–11]. Owing to their good electron donating ability and reversible electrochemical activity, TTF derivatives have recently been employed as building blocks for functional supramolecular systems [12–22]. Among the TTF-based supramolecular systems, those involving fullerene molecules are of growing interest due to their potential application in organic voltaics [20]. The TTF–fullerene dyad is a typical donor–acceptor (D–A) system, where TTFs and fullerenes act as donors and acceptors, respectively. The TTF–fullerene dyad can be constructed by (1) connection of two components through covalent bonds [23–27] or (2) supramolecular assembly between TTFs (as host) and fullerene (as guest) [28–41].

For the formation of the host–guest type supramolecular system, the shape and size complementarity between TTFs and fullerenes are key factors that give rise to the effective surface contact for the stabilization of the resulting supramolecular structures. Because pristine TTF cannot form good enough surface contact with fullerenes due to the shape and size mismatch [42], chemical modifications of TTF have been carried out. To this regard, introduction of substituents onto the peripheral sites [43–54] and expansion of the π-systems have been reported [55–59]. As reported, the π-extended TTFs (exTTF) can encapsulate fullerenes in solution, and form the inclusion complex with fullerenes as well [28–37]. Very recently, we have disclosed a facile approach toward the arylthio-substituted TTFs (hereafter denoted as Ar-S-TTF) [60–62], which bear four aryl groups on the peripheral positions of the TTF core through the sulfur bridges. The Ar-S-TTF molecules are size and shape matched for fullerenes (C_60_/C_70_), and the peripheral aryls show large rotational freedom that could adjust their spatial alignment to adapt to the environmental variations [61].

Regarding the structural feature of Ar-S-TTF, we have performed the complexation of Ar-S-TTFs with C_60_/C_70_, and found that Ar-S-TTF could form D–A type inclusion complexes with C_60_/C_70_ [63]. Crystallographic investigation reveals that the multidimensional interaction networks consisting of a central TTF core, peripheral aryls, and fullerenes are the key factors to stabilize the resulting supramolecular structures. Meanwhile, the solid state absorption study indicates that the inclusion complexes display a photoexcited electronic transition between Ar–S–TTF and C_60_/C_70_. To gain further insight into the structural features of Ar-S-TTF upon complexation with C_60_/C_70_, we have carried out the preparation a series of [(Ar-S-TTF)–(fullerene)] co-crystals and investigated their solid state structure, as reported herein. In this report we focus on the synthesis, composition (donor:acceptor ratio), and crystal structure of the resulting co-crystals.

## Results and Discussion

### Synthesis and compositions

On the basis of hundreds of experimental runs, we found that the Ar-S-TTFs possessing the first redox potential (*E*_1/2_^1^) smaller than 0.6 V could form the co-crystal-type complexes with fullerene molecules C_60_ and/or C_70_, whereas those with *E*_1/2_^1^ > 0.6 V could not afford the desired complexes. The complexes obtained thus far are intrinsically neutral [63], which means the charge transfer does not take place between Ar-S-TTF and fullerenes in the ground state. In this regard, the *E*_1/2_^1^ values of Ar-S-TTFs would not affect the formation of co-crystals. On the other hand, the interaction between the aryls and fullerene molecules is very important to stabilize the structure of the co-crystals as reported in the crystal structures section. For Ar-S-TTFs exhibiting *E*_1/2_^1^ > 0.6 V, the aryl groups are more electron deficient than the phenyl [61–62]. The interaction between the electron-deficient aryls and fullerenes is weak; consequently, these TTFs (*E*_1/2_^1^ > 0.6 V) could not form the co-crystals with fullerenes.

**Scheme 1:**
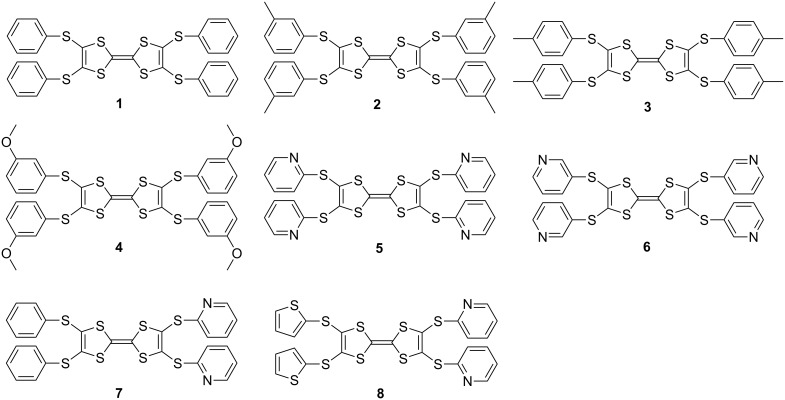
Chemical structures of Ar-S-TTFs **1**–**8**.

Among the [(Ar-S-TTF)–(fullerene)] complexes obtained to this point, the complexes of Ar-S-TTFs **1**–**8** (Scheme 1) with C_60_/C_70_ were cropped in the single crystalline form, and the others were obtained as powdery samples with difficult to determine compositions. The [(Ar-S-TTF)–(fullerene)] co-crystals were prepared by evaporating the mixed solution of Ar-S-TTF and the corresponding fullerenes at room temperature. As a typical example, compound **1** (12.8 mg, 2 mmol) and C_60_ (7.2 mg, 1 mmol) were dissolved in carbon disulfide (CS_2_, 7 mL), and the resulting mixed solution was then placed in the dark hood and left standing without disruption. After 2 weeks, the black block-like single crystalline complex was cropped, and the composition of the complex was determined to be **1**·(C_60_)_2_·(CS_2_)_2_ on the basis of the X-ray single crystal structure analysis. The synthetic conditions, compositions, yields, and morphologies for the 11 complexes are summarized in Table 1.

**Table 1:** Experimental conditions for the preparation of the co-crystals.

	Donor	Acceptor	Solvent^a^	Complex^b^	Appearance

C_60_ complexes	**1**, 12.8 mg	C_60_, 7.2 mg	CS_2_, 7 mL	**1**·(C_60_)_2_·(CS_2_)_2_, 7.3 mg	Black block
	**1**, 7 mg	C_60_, 5.7 mg	PhCl, 6 mL	**1**·(C_60_)_2_·PhCl, 7.5 mg	Black block
	**2**, 6.9 mg	C_60_, 5.5 mg	PhCl, 14 mL	**2**·C_60_, 8.2 mg	Black plate^c^
	**4**, 34.1 mg	C_60_, 14.4 mg	CS_2_, 14 mL	**4**·C_60_·CS_2_, 29.2 mg	Black block^c^
	**6**, 10 mg	C_60_, 11 mg	PhCl, 9 mL	**6**·(C_60_)_2_·(PhCl)_2_, 17.1 mg	Black block
	**7**, 14.4 mg	C_60_, 5.5 mg	PhCl, 12 mL	**7**·C_60_, 8.8 mg	Black plate
	**8**, 14.6 mg	C_60_, 5.5 mg	PhCl, 12 mL	**8**·C_60_, 9.7 mg	Black block

C_70_ complexes	**1**, 9 mg	C_70_, 5.9 mg	PhCl, 12 mL	**1**·C_70_, 9.2 mg	Black plate
	**2**, 6.9 mg	C_70_, 4.2 mg	PhCl, 10 mL	**2**·(C_70_)_4_·(PhCl)_2_, 6.1 mg	Black prism
	**3**, 13.8 mg	C_70_, 8.4 mg	PhCl, 10 mL	**3**·(C_70_)_2_·(PhCl)_2_, 6 mg	Black prism^c^
	**5**, 6.5 mg	C_70_, 4.2 mg	PhCl, 8 mL	**5**·C_70_, 4.1 mg	Black block

^a^CS_2_: carbon disulfide, PhCl: chlorobenzene; ^b^the compositions of the co-crystals were determined by X-ray single crystal structure analysis; ^c^see reference [63].

Concerning the compositions (donor:acceptor ratio), most of the co-crystals could be divided into two types (I and II), except for **2**·(C_70_)_4_·(PhCl)_2_. The ratio of donor:acceptor (abbreviated as D:A) for the type I and type II co-crystals are 1:1 and 1:2, respectively. No solvent molecule was involved in most of the type I co-crystals, except for **4**·C_60_·CS_2_, which contains a small, linear solvent, CS_2_. On the other hand, all of the type II co-crystals contain solvent molecules in their matrix. Since one Ar-S-TTF is capable of encapsulating a fullerene molecule [63], the larger ratio of fullerenes would result in the formation of additional void space by fullerene molecules, which could potentially accommodate the solvent molecules. In a previous report, we have proposed that C_70_ tends to form co-crystals with a larger acceptor ratio [63]. However, the present results suggest that this prediction would not hold because both C_60_ and C_70_ form the type I and type II co-crystals with Ar-S-TTFs as shown in Table 1. The D:A ratio for the co-crystals results from the cooperative effects of the geometry of Ar-S-TTF (particularly the peripheral aryls), the shape and size of the fullerene molecules, and the solvent molecules. Although we cannot presently provide a clear estimation of the D:A ratio of the co-crystals, this work further demonstrates that Ar-S-TTFs are promising candidates to serve as receptors for fullerenes and have diverse supramolecular structures.

### Crystal structures

The single crystalline structure of the complex is suitable for X-ray single crystal diffraction measurements. In most cases, the fullerene molecules and solvent molecules are disordered. The disorder of fullerenes and solvents cannot be suppressed even at low temperature, and can thus be characterized as having statistic rather than rotational disorder. The selected crystallographic data are summarized in Tables S1 and S2 in Supporting Information File 1. In the following sections, the structures of the type I and type II co-crystals will be discussed in sequence.

#### Type I co-crystals

The type I co-crystals include four C_60_ complexes and two C_70_ complexes, namely, **2**·C_60_, **4**·C_60_·CS_2_, **7**·C_60_, **8**·C_60_, **1**·C_70_, and **5**·C_70_. It should be noted that we recently reported the structures of **2**·C_60_ and **4**·C_60_·CS_2_ [63]. As a typical example of the newly obtained co-crystals, the structure of **5**·C_70_ is discussed here, and those of **7**·C_60_, **8**·C_60_, and **1**·C_70_ are provided in the Supporting Information File 1 (Figures S4–S15).

**Figure 1:**
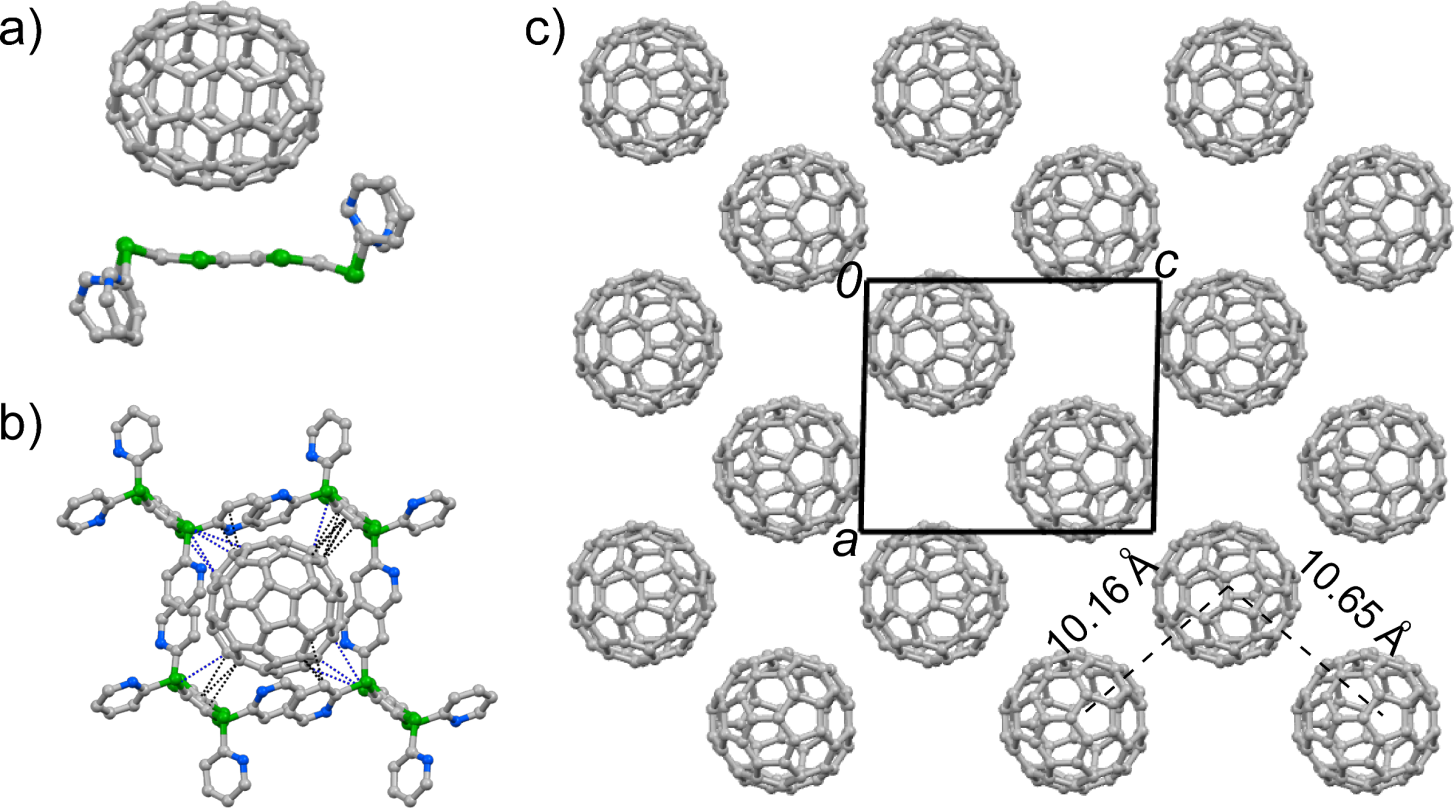
Crystal structure of **5**·C_70_. a) Unit cell contents viewed along the short axis of **5**; b) Interactions between **5** and C_70_, where the blue and black dashed lines represent the C–S and C–C contacts, respectively; c) packing motif of C_70_ molecules viewed along the *b*-axis. The grey, blue and green spheres represent carbon, nitrogen and sulfur atoms, respectively, and the hydrogen atoms are omitted for clarity.

Complex **5**·C_70_ crystallizes in the triclinic space group *P*-1 with one molecule **5** and one C_70_ crystallographically unique (Figure 1a). The central TTF core on molecule **5** has a chair conformation. The molecular geometry of **5** in the co-crystal, both the spatial alignment of pyridyl groups and the conformation of the TTF core, is very close to its neutral crystalline form obtained in CS_2_ [61]. The C_70_ molecule is surrounded by four molecules **5** (Figure 1b), and the long axes of C_70_ and TTF5 are almost parallel to each other. A pair of **5** and a C_70_ molecule in a sandwich configuration is stabilized by multiple intermolecular atomic close contacts [64]: C–S of 3.33–3.36 Å and C–C of 3.17–3.40 Å. The other two molecules of **5** have C–S contacts (3.23–3.50 Å) with C_70_ along their lateral axes. Moreover, the C–C contacts (3.18–3.36 Å) are also observed between the peripheral pyridyl groups and C_70_. Compound **5** and C_70_ form separated layers (Figures S1–S3 in Supporting Information File 1), and the arrangement of C_70_ molecules in the *ac*-plane is shown in Figure 1c. The center-to-center distances between C_70_ molecules along the (*a*−*c*) and (*a*+*c*) directions are 10.2 Å and 10.6 Å, respectively. The former is close to the short axis of C_70_ (10 Å), thus C_70_ molecules form one-dimensional (1D) columnar arrays along this direction. The structural features for both donor–acceptor interaction mode and fullerene arrangement of **7**·C_60_, **8**·C_60_, and **1**·C_70_ are very similar to that of **5**·C_70_.

#### Type II co-crystals

Among the type II co-crystals, the structure of **3**·(C_70_)_2_·(PhCl)_2_ has been reported [63]. Herein, we report the structures of **1**·(C_60_)_2_·(CS_2_)_2_ and **1**·(C_60_)_2_·PhCl, and that of **6**·(C_60_)_2_·(PhCl)_2_ is provided in the Supporting Information File 1 (Figures S25–S29).

**Figure 2:**
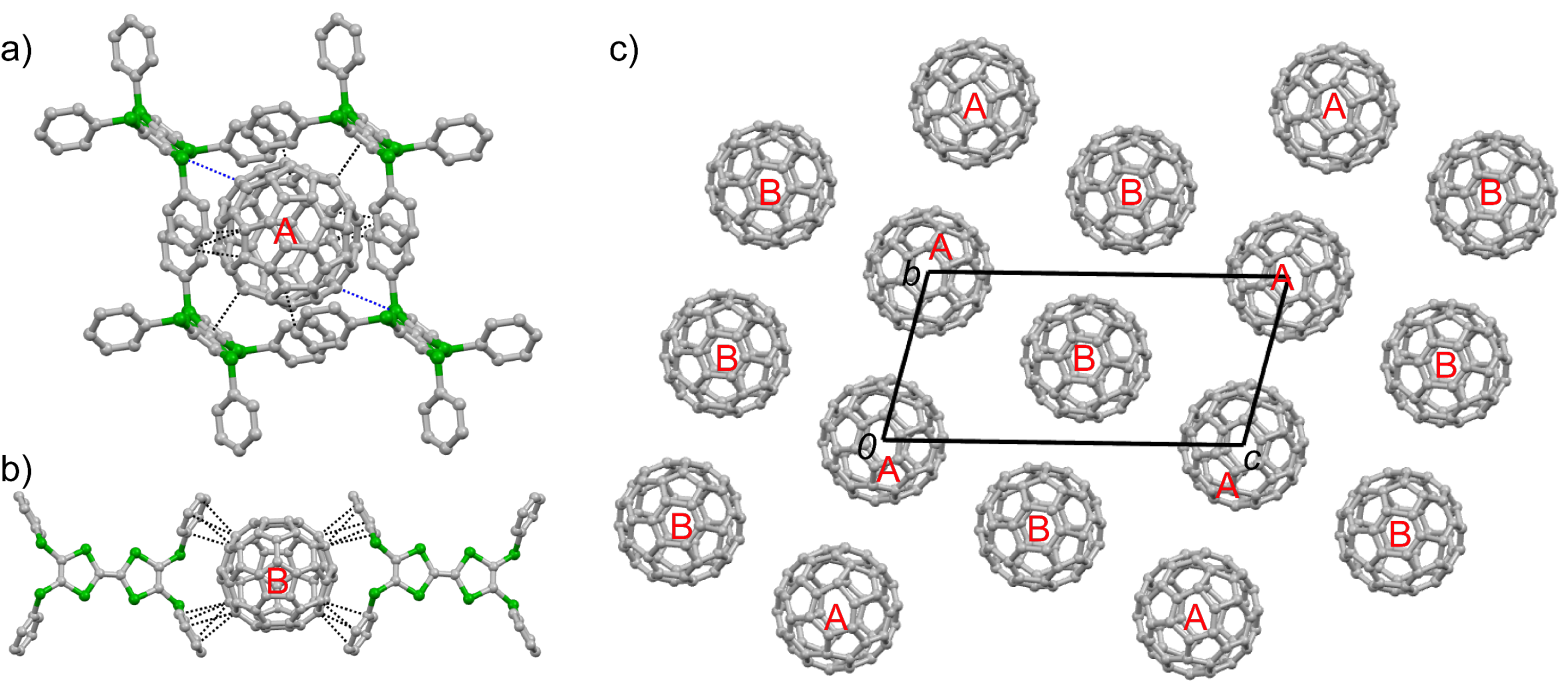
Crystal structure of **1**·(C_60_)_2_·(CS_2_)_2_. a) Interactions of C_60_ molecule **A** with **1**, where the blue and black dashed lines indicate the C–S and C–C contacts between **1** and C_60_, respectively. b) Interactions of C_60_ molecule **B** with **1**; c) packing motifs of C_60_, depiction along the *a*-axis. The hydrogen atoms and solvent molecules are omitted for clarity.

**Figure 3:**
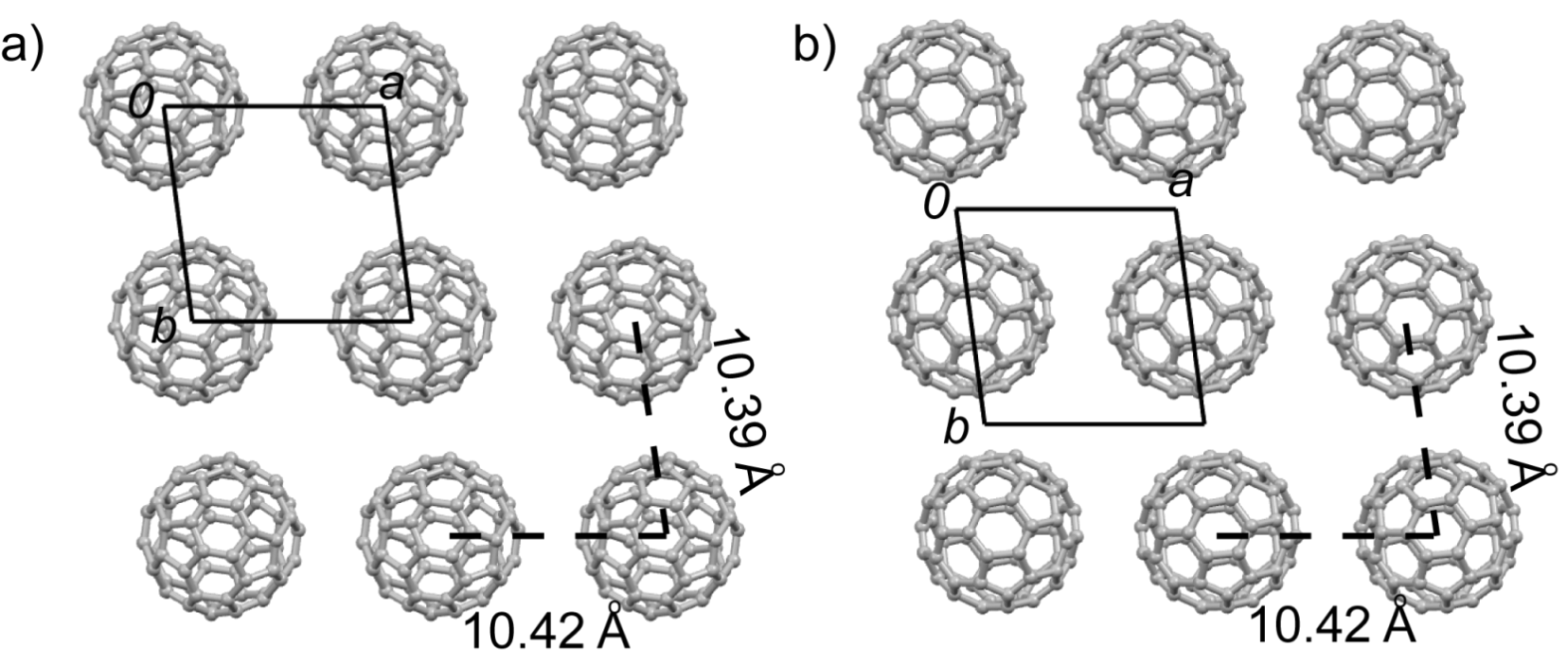
Packing motifs of C_60_ molecules **A** (a) and **B** (b) of **1**·(C_60_)_2_·(CS_2_)_2_ in the crystallographic *ab*-plane with the center-to-center distance between the neighboring C_60_ molecules shown.

Complex **1**·(C_60_)_2_·(CS_2_)_2_ crystallizes in the triclinic space group *P*-1. The asymmetric unit contains half a molecule of **1**, two halves of C_60_ (**A** and **B**), and one CS_2_ molecule. The central C_6_S_4_ moieties of both molecules **1** (**A** and **B**) are nearly planar. Molecule **A** is disordered and surrounded by four molecules of **1**. The donor–acceptor interaction mode for molecule **A** is very similar to those in type I co-crystals (Figure 2a): a pair of molecules of **1** forms a sandwich structure with **A** through multiple interactions (C–S, 3.50 Å; C–C, 3.20–3.38 Å), and the other two molecules of **1** interact with **A** along their lateral axes. On the other hand, the C_60_ molecule **B** is ordered and surrounded by four phenyl groups with multiple C–C contacts (3.19–3.38 Å) as shown in Figure 2b. **1** and C_60_ form separated layers (Figures S16–S17 in Supporting Information File 1). The packing structure of C_60_ in the *bc*-plane is shown in Figure 2c. Molecules **A** and **B** form two kinds of two-dimensional (2D) sheets in the *ab*-plane as shown in Figure 3, and two sheets alternate along the *c*-axis. In both 2D sheets, the center-to-center distances between adjacent C_60_ molecules are 10.4 Å along the *a*- and *b*-axes.

Complex **1**·(C_60_)_2_·PhCl crystallizes in the triclinic space group *P*-1. Unlike **1**·(C_60_)_2_·(CS_2_)_2_, the asymmetric unit of **1**·(C_60_)_2_·PhCl contains one molecule of **1**, four halves of C_60_ (**A**, **B**, **C**, and **D**), and one PhCl molecule. The central TTF core of **1** is in a planar conformation. As shown in Figure S18 in Supporting Information File 1, C_60_ molecules **A** and **B** have a donor–acceptor interaction mode similar to those in the type I co-crystals, and several C–C (3.17–3.39 Å) and C–S (3.46–3.49 Å) contacts are observed between the central TTF core of **1** and C_60_ molecules. On the other hand, C_60_ molecules **C** and **D** are surrounded by the phenyl groups of **1** and solvent molecules PhCl (Figure S19 in Supporting Information File 1). The donor and acceptor molecules that form the separated layers are shown in Figures S20–S24 (Supporting Information File 1). Molecules **A** and **B** form a 2D sheet in the *ab*-plane (Figure S23 in Supporting Information File 1), where **A** and **B** form the different columnar arrays along the *a*-axis. The center-to-center distances between the adjacent C_60_ molecules are around 10.4 Å along the *a*- and *b*-axes. Molecules **C** and **D** also form the 2D sheet in the *ab*-plane (Figure S24 in Supporting Information File 1), and the configuration of the molecular arrays is similar to that in the **AB** sheet. The **AB** and **CD** sheets alternate along the crystallographic *c*-axis.

#### Co-crystal **2**·(C_70_)_4_·(PhCl)_2_

**Figure 4:**
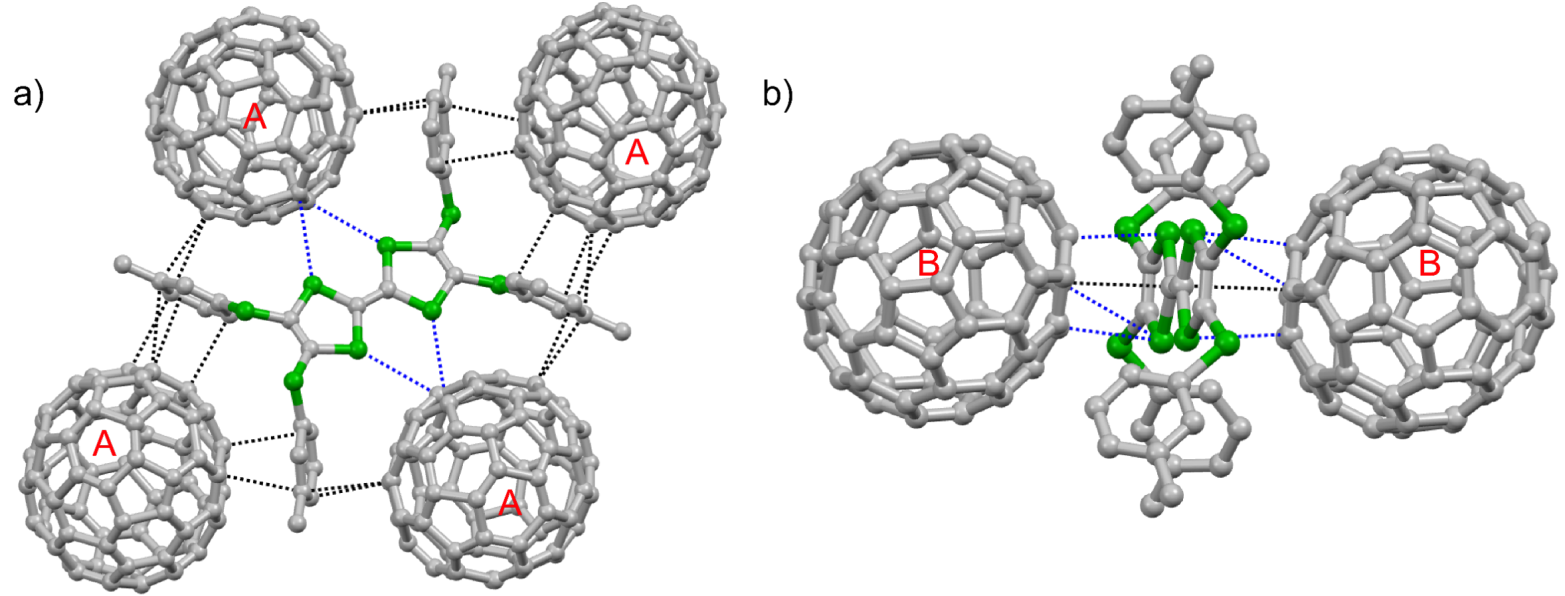
Crystal structure of **2**·(C_70_)_4_·(PhCl)_2_. a) Interactions between C_70_ molecule **A** and **2**; b) Interactions between C_70_ molecule **B** and **2**. The blue and black dashed lines indicated the intermolecular C–S and C–C contacts, respectively. The hydrogen atoms are omitted for clarity.

This complex crystallizes in the monoclinic space group *C*2/*n*, and the asymmetric unit contains half of molecule **2**, two C_70_ molecules (**A** and **B**), and two halves of PhCl molecules. The central C_6_S_4_ moiety of molecule **2** is nearly planar. In types I and II co-crystals, fullerene molecules are encapsulated by Ar-S-TTF molecules. However, in **2**·(C_70_)_4_·(PhCl)_2_, molecule **2** penetrates into the void space formed by C_70_ molecules due to the large A:D ratio (4:1). Referring Figure 4, one molecule **2** is surrounded by six C_70_ neighbors. Along the longitudinal and lateral axes of molecule **2**, four **A** molecules are located to show multiple donor–acceptor interactions (Figure 4a): C–C contacts (3.22–3.37 Å) between *p*-tolyl moieties and C_70_ along the longitudinal axis of **2**, and C–S contacts (3.44, 3.47Å) between the central TTF core and C_70_ along the lateral axis of molecule **2**. On the other hand, two **B** molecules are located above and below the mean plane of **2** (Figure 4b), and there are C–S (3.44–3.50 Å) and C–C (3.13, 3.35 Å) contacts between the C_70_ molecules and the central TTF core of **2**. The C_70_ molecules form the 3D network of the present co-crystals. As shown in Figure S30 Supporting Information File 1, one **A** molecule is surrounded by five **B** molecules, and one **B** molecule is surrounded by five **A** molecules. There are multiple C–C close contacts (3.20–3.38 Å) between the neighboring C_70_ molecules along the different directions, which result in the 3D carrier transport pathway [38–41].

#### Structural comparison

As has previously been reported, the van der Waals length of the C_6_S_8_ core in the Ar-S-TTF molecule is about 12.8 Å [61,63], which is larger than the van der Waals diameters of C_60_ (10 Å) and C_70_ (11 Å) [65–66]. In this regard, a single Ar-S-TTF molecule is able to encapsulate C_60_/C_70_, and the size difference between C_60_ and C_70_ would not be the sole factor determining the composition of the co-crystal. The D:A ratio for the co-crystal is attributed to the cooperation of the geometry of the Ar-S-TTF (particularly the geometry and rotational freedom of the peripheral aryls), the shape and the size of the fullerene molecules, and the solvent molecules. Furthermore, the dynamic effect on the crystal growth is also taken into account.

Additionally, the D:A ratio of the co-crystal plays a significant role on the donor–acceptor interaction mode and the packing motif of the fullerenes. In the type I co-crystals (D:A = 1:1), the donor–acceptor interactions mainly exist between the central TTF core of the Ar-S-TTF and the fullerene molecules. Therefore, Ar-S-TTF serves as the host and the fullerene molecule is the guest. The fullerene molecules form the 1D columnar stacks with a center-to-center distance of around 10.3 Å, which is comparable with that of superconducting C_60_ complexes with alkali metals (e.g.,10.29 Å in RbCs_2_C_60_) [67]. When the ratio of fullerene molecules increases (i.e., the type II co-crystals, D:A = 1:2), one fullerene molecule is substantially encapsulated by the central TTF core of Ar-S-TTF, and another fullerene molecule is surrounded by the aryls on the Ar-S-TTF and solvent molecules. In this case, the Ar-S-TTF molecule still acts as the host for at least one of the fullerene molecules. Moreover, the dimensionality of the packing motifs of fullerene molecules increases, resulting in a 2D network through multiple van der Waals forces. Upon further increase of the ratio of fullerene molecules in the co-crystal (e.g., **2**·(C_70_)_4_·(PhCl)_2_), the packing structure becomes dominated by the C_70_ molecule, which forms the 3D network. The Ar-S-TTF and solvent molecules serve as the guests to occupy the void formed by the C_70_ molecules.

The present results demonstrate that Ar-S-TTF molecules have three key features that enable formation of donor–acceptor type co-crystals with fullerenes: (1) size and shape complementarity, (2) flexibility, and (3) the ability to introduce an additional interaction with fullerene by peripheral aryls. While the interactions between the TTF framework and fullerenes have been observed in many TTF–fullerene supramolecular systems [38–41], the rotational freedom of the peripheral aryls on Ar-S-TTF causes the aryls to locate at the appropriate positions to form additional interactions with fullerenes. This therefore enhances the stability of the resulting co-crystals. By complexation with Ar-S-TTF molecules, the 1D columnar array, 2D sheets, and 3D networks of fullerene molecules have been successfully established, resulting in the carrier transport pathway, in principle. However, the ground state of the present co-crystals is intrinsically neutral, as demonstrated by the IR spectra (Figures S34–S40 in Supporting Information File 1). To improve the functionality of the co-crystals, one interesting strategy would be the generation of itinerant carriers in the co-crystals, i.e., charge transfer between the donor and acceptor in the ground state.

## Conclusion

In summary, we have prepared eleven donor–acceptor type co-crystals of Ar-S-TTFs with fullerenes (C_60_/C_70_), and performed thorough investigations on their solid state structures. These co-crystals mainly belong to two types according to the donor:acceptor ratios (D:A), types I and II having D:A of 1:1 and 1:2, respectively. The composition of the co-crystals is thought to be the cooperative consequence of the molecular geometry of Ar-S-TTF, the shape and size of the fullerene molecule, the solvent adduct, and the crystallization dynamics. The donor–acceptor interaction mode and the packing motif of the fullerenes largely depend on the composition of the co-crystal. The present results suggest that Ar-S-TTF molecules would be promising receptors for fullerenes as they are easily accessible. Meanwhile, Ar-S-TTFs possess the unique structural features to encapsulate fullerenes. That means the size matches with that of fullerenes and the flexibility is helpful to adapt to the shape of the fullerenes, which is supported by the additional stabilization force from the peripheral aryls moieties. Moreover, by varying the peripheral aryls and solvents, the 1D, 2D, and 3D packing motifs of fullerenes can be selectively achieved.

## Supporting Information

File 1 Additional experimental data.

File 2 Crystallographic data in CIF format.
