# Supplementary material for: Donor–acceptor type co-crystals of arylthio-substituted tetrathiafulvalenes and fullerenes
Source: Beilstein J Org Chem. 2015 Jun 19;11:1043–51. doi: 10.3762/bjoc.11.117 (PMC4505149; doi:10.3762/bjoc.11.117)
Supplement: File 1 — Additional experimental data. [file Beilstein_J_Org_Chem-11-1043-s001.pdf]

Supporting Information
for

Donor–acceptor type co-crystals of arylthio-substituted tetrathiafulvalenes and fullerenes

Xiaofeng Lu, Jibin Sun, Shangxi Zhang, Longfei Ma, Lei Liu, Hui Qi, Yongliang Shao, and Xiangfeng Shao*

Address: State Key Laboratory of Applied Organic Chemistry, Lanzhou University, Tianshui Southern Road 222, Lanzhou 730000, Gansu Province, P. R. China.

Email: Xiangfeng Shao - shaoxf@lzu.edu.cn

* Corresponding author

Experimental

Chlorobenzene (PhCl) and carbon disulfide (CS_2) were distilled over P_2O_5 under inert conditions and stored in a long-necked flask under an argon atmosphere. **1**–**8** were prepared according to a method reported in the literature [1–3] and further purified by recrystallization from CH_2Cl_2/*n*-hexane to give red crystalline samples. C_{60} and C_{70} were purchased from NiChem Fine Technology Co. Ltd (Taiwan) and further purified by recrystallization from toluene.

The samples for the crystallographic analyses of the complexes were obtained by slow evaporation of the solvent. **1**•$(C_{60})_2$•$(CS_2)_2$ was selected as an example. It was prepared by slow evaporation of the mixed solution of **1** (12.8 mg, 2×10^{-5}mol) and C_{60} (5.7 mg, 1×10^{-5}mol) in CS_2 (7 ml) at room temperature (RT). After 15 days, the black block single crystals were formed. The complex was collected by suction, washed with a mixture of CH_2Cl_2/petroleum ether, then dried in vacuo (7.3 mg were obtained).

The X-ray diffraction measurements was carried out on SuperNova (Agilent) type diffractometer. The crystal structures of complexes were solved by a direct methods *SIR2004* [4] and refined by the full-matrix least-squares method on F^2 by means of *SHELXL-97* [5]. The calculated positions of the hydrogen atoms were included in the final refinement. The crystallographic data are summarized in Table S1 and Table S2. The absorption spectra were measured in solid state by dispersing the complexes in a KBr pellet, respectively. Fourier transform infrared (FT-IR) spectra (400–4000 cm^{-1}) were recorded on a Thermo Electron Corporation Nicolet Nexus 670 FT-IR Spectrometer with a resolution of 4 cm^{-1}. The experimental PXRD patterns were collected at 25 °C on a X'Pert PRO (PANalytical) X-ray diffractometer using Cu*Kα*-radiation (λ = 1.5418 Å) with the generator settings 40 kV and 40 mA. Thermogravimetric analyses (TGA) were conducted on 1090B type thermal analyzer (Dupont Engineering Polymers).

Table S1: Selected crystallographic data for the type I co-crystals.

	**7**•C_{60}	**8**•C_{60}	**1**•C_{70}	**5**•C_{70}
CCDC number				
Empirical formula	$C_{88}H_{18}N_2S_8$	$C_{84}H_{12}N_2S_{10}$	$C_{100}H_{20}S_8$	$C_{96}H_{16}N_4S_8$
Formula weight	1359.52	1369.56	1478.65	1481.61
Temperature / K	136(2)	107(10)	150(1)	110(14)
λ / Å	0.71073	0.71073	1.54184	1.54184
Crystal size / mm^3	0.08×0.12×0.18	0.24×0.28×0.35	0.01×0.15×0.17	0.05×0.06×0.11
Crystal system	Triclinic	Triclinic	Triclinic	Triclinic
space group	*P*-1	*P*-1	*P*1	P-1
a / Å	10.3135(4)	10.1099(9)	10.3115(7)	13.3996(6)
b / Å	10.3164(6)	10.6008(11)	10.6597(7)	14.3549(7)
c / Å	13.7643(11)	13.3530(17)	14.2727(14)	15.9348(8)
α / °	68.271(7)	67.122(11)	68.533(8)	71.206(4)
β / °	86.126(5)	82.300(9)	88.990(7)	87.261(4)
γ / °	79.343(15)	79.207(8)	80.494(6)	77.386(4)
V / Å3	1336.98(15)	1292.2(3)	1438.4(2)	2830.8(2)
Z	1	1	1	2
d_{calc} / g cm^{-3}	1.689	1.760	1.707	1.738
μ / mm^{-1}	0.398	0.490	3.39	3.469
$2\theta_{max}$ / °	55.82	57.27	155.79	140.12
Data / restraints / parameters	4832 / 318 / 442	5392/1048/866	6249/ 701 / 817	10738 / 696 / 973
GooF	1.154	1.039	1.054	1.149
R [$I > 2\sigma(I)$]	0.0840	0.0845	0.1163	0.0838
wR2	0.2130	0.2069	0.2271	0.2240

Table S2: Selected crystallographic data for the type II co-crystals and the exceptional one.

	**1**•$(C_{60})_2$•$(CS_2)_2$	**1**•$(C_{60})_2$•PhCl	**6**•$(C_{60})_2$•$(PhCl)_2$	**2**•$(C_{70})_4$•$(PhCl)_2$
CCDC number				
empirical formula	$C_{152}H_{20}S_{10}$	$C_{156}H_{20}S_8Cl$	$C_{158}H_{30}N_4S_8Cl_2$	$C_{326}H_{28}S_8Cl_2$
formula weight	2230.40	2185.65	2307.21	4270.86
temperature / K	113(2)	291.89(10)	113(2)	113(2)
λ / Å	0.71073	0.71073	0.71073	0.71073
crystal size / mm^3	0.20×0.20×0.20	0.12×0.15×0.20	0.20×0.20×0.20	0.05×0.05×0.20
crystal system	Triclinic	Triclinic	Triclinic	Monoclinic
space group	*P*-1	*P*-1	*P*-1	*C*2/n
a / Å	10.422(4)	10.4255(7)	10.439(4)	22.859(5)
b / Å	10.386(3)	20.8257(19)	10.466(4)	27.904(6)
c / Å	21.722(7)	21.2111(11)	42.064(18)	26.409(6)
α / °	73.300(16)	101.302(6)	83.276(15)	90
β / °	73.605(16)	101.293(5)	83.192(15)	92.808(5)
γ / °	78.378(19)	100.743(6)	79.164(17)	90
V / Å3	2142.0(13)	4310.0(6)	4461.0(3)	16825.0(7)
Z	1	2	2	4
d_{calc} / g cm^{-3}	1.729	1.684	1.718	1.686
μ / mm^{-1}	0.380	0.313	0.337	0.223
$2\theta_{max}$ / °	52.04	57.94	52.05	52.05
Data / restraints / parameters	8341/ 515 / 720	19916 / 1002 / 1387	13413 / 1040 / 1438	16564 / 1032 / 1534
GooF	1.071	1.896	1.092	1.085
R [$I > 2\sigma(I)$]	0.1067	0.1826	0.1195	0.0856
wR2	0.2238	0.3598	0.2620	0.1793

References

1. Sun, J.; Lu, X.; Shao, J.; Li, X.; Zhang, S.; Wang, B.; Zhao, J.; Shao, Y.; Fang, R.; Wang, Z.; Yu, W.; Shao, X. *Chem. Eur. J.*, **2013**, *19*, 12517. doi: 10.1002/chem.201301819
2. Sun, J.; Lu, X.; Shao, J.; Cui, Z.; Shao, Y.; Jiang, G.; Yu, W.; Shao, X. *RSC Adv.*,**2013**, *3*, 10193. doi: 10.1039/C3RA41349G
3. Sun, J.; Lu, X.; Ishikawa, M.; Nakano, Y.; Zhang, S.; Zhao, J.; Shao, Y.; Wang, Z.; Yamochi, H.; Shao, X. *J. Mater. Chem. C*, **2014**, *2*, 8017. doi: 10.1002/chem.201402327
4. Burla, M. C.; Caliandro, R.; Camalli, M.; Carrozzini, B.; Cascarano, G. L.; de Caro, L.; Giacovazzo, C.; Polidori, G.; Spagna, R. *J. Appl. Cryst.*, **2005**, *38*, 381. doi: 10.1107/S002188980403225X
5. Sheldrick, G. M. SHELXL-97, *A Program for Crystal Structure Refinement*. University of Göttingen, Göttingen, Germany, **1997**.

Crystal structures

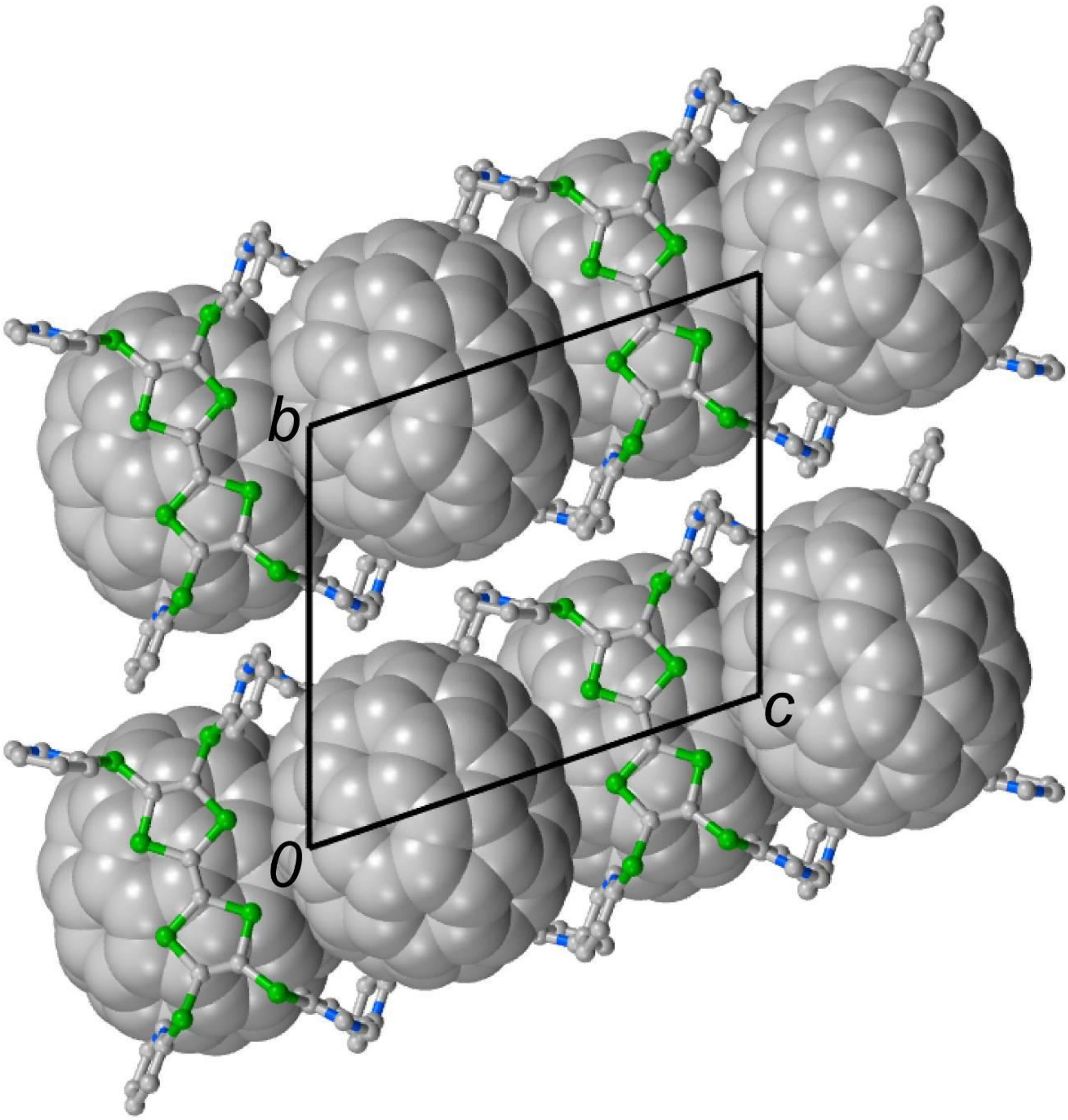

Figure S1: Crystal structure of **5**•C_{70} projected along the crystallographic *a*-axis. The C_{70} molecules are drawn in spacefill style and the hydrogen atoms omitted for clarity.

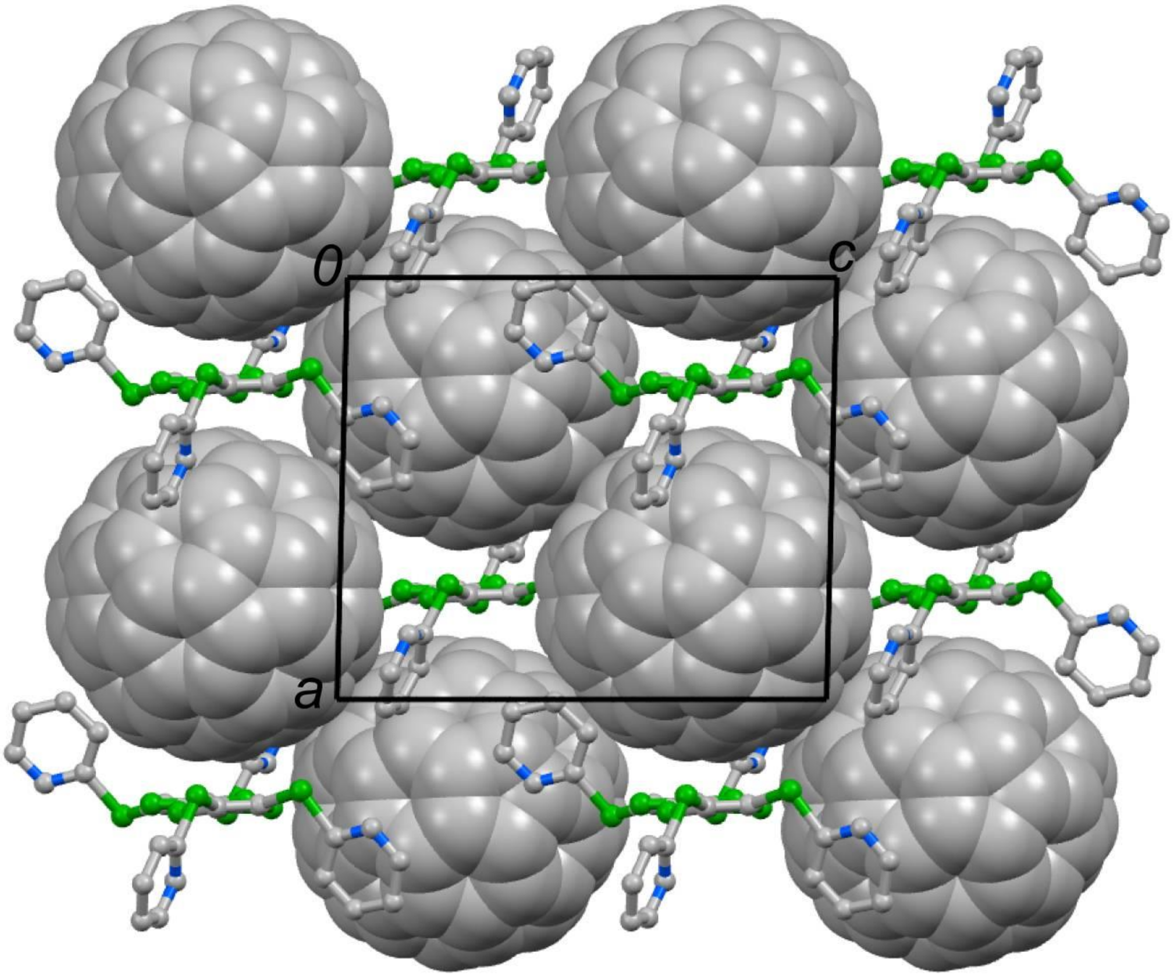

Figure S2: Crystal structure of **5**•C_{70} projected along the crystallographic *b*-axis. The C_{70} molecules are drawn in spacefill style and the hydrogen atoms omitted for clarity.

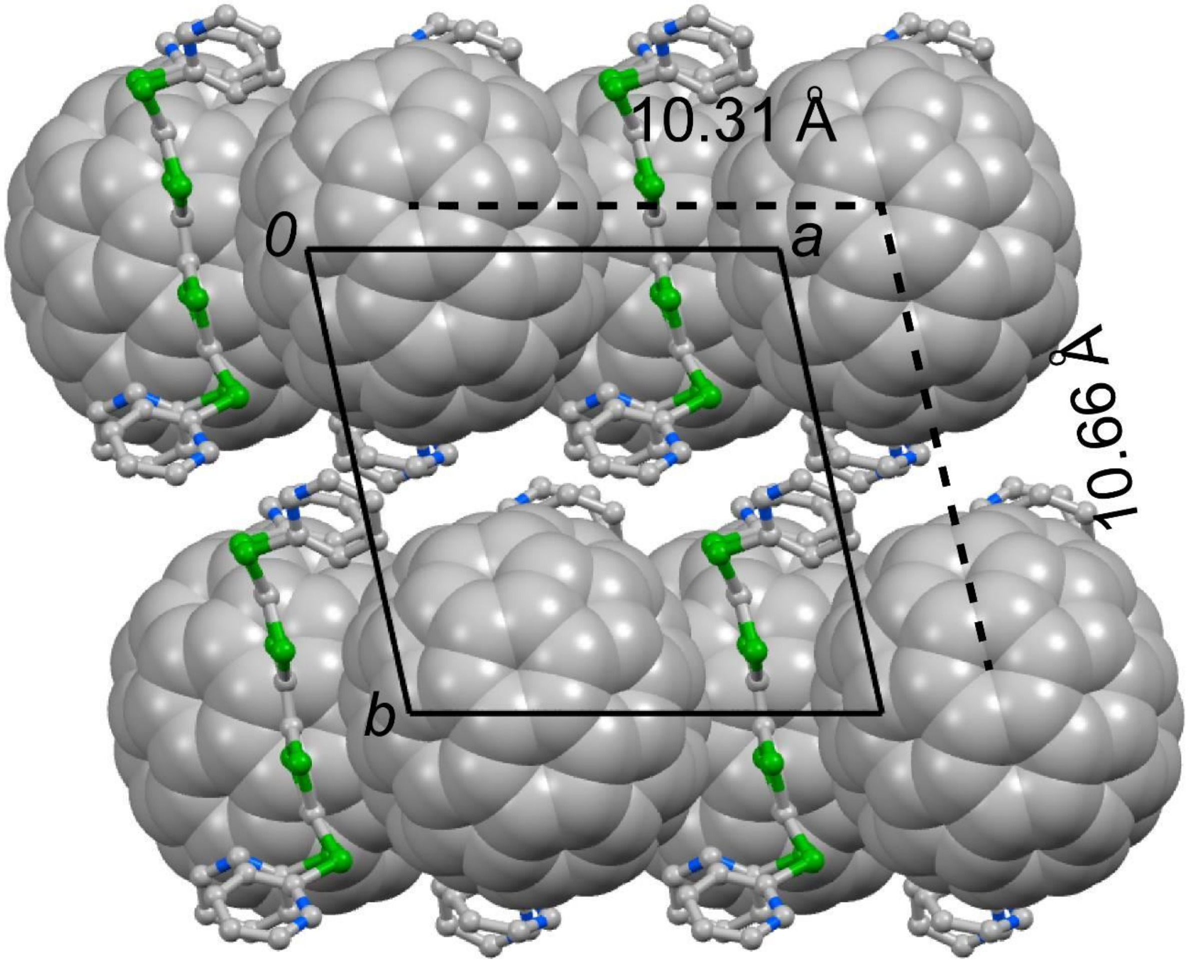

Figure S3: Crystal structure of **5**•C_{70} projected along the crystallographic *c*-axis. The C_{70} molecules are drawn in spacefill style and the hydrogen atoms omitted for clarity.

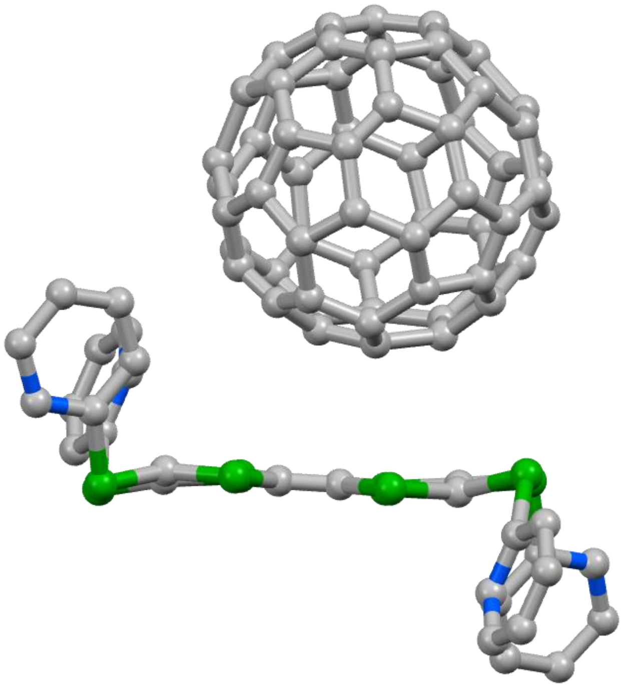

Figure S4: The unit cell contents of **7**•C_{60} viewed along the short axis of **7**. The hydrogen atoms are omitted for clarity. The complex crystallizes in the triclinic space group *P*-1, and the asymmetric unit contains one **7** and one C_{60}. The central TTF core on **7** is nearly planar. The phenyl groups and the 2-pyridyl groups are disordered. Thus, it is difficult to distinguish the phenyl and 2-pyridyl groups by the single crystal structure analysis.

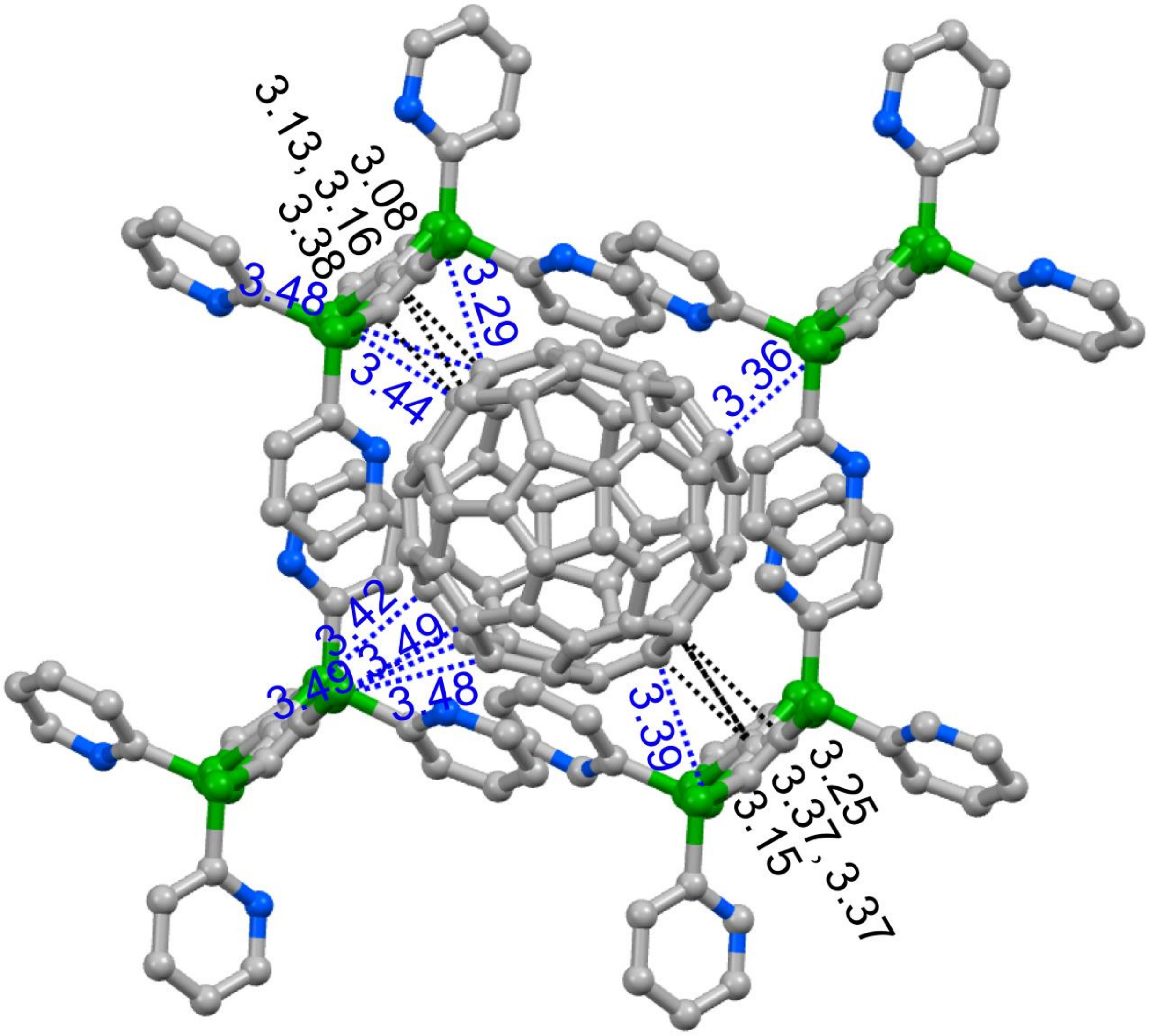

Figure S5: Intermolecular atomic short contacts (blue and black dashed lines for C–S and C–C contacts respectively, in unit of Å) between the central core of **7** and C_{60} in **7**•C_{60}. The grey, blue and green balls represent carbon, nitrogen and sulfur atoms, respectively, and the hydrogen atoms are omitted for clarity.

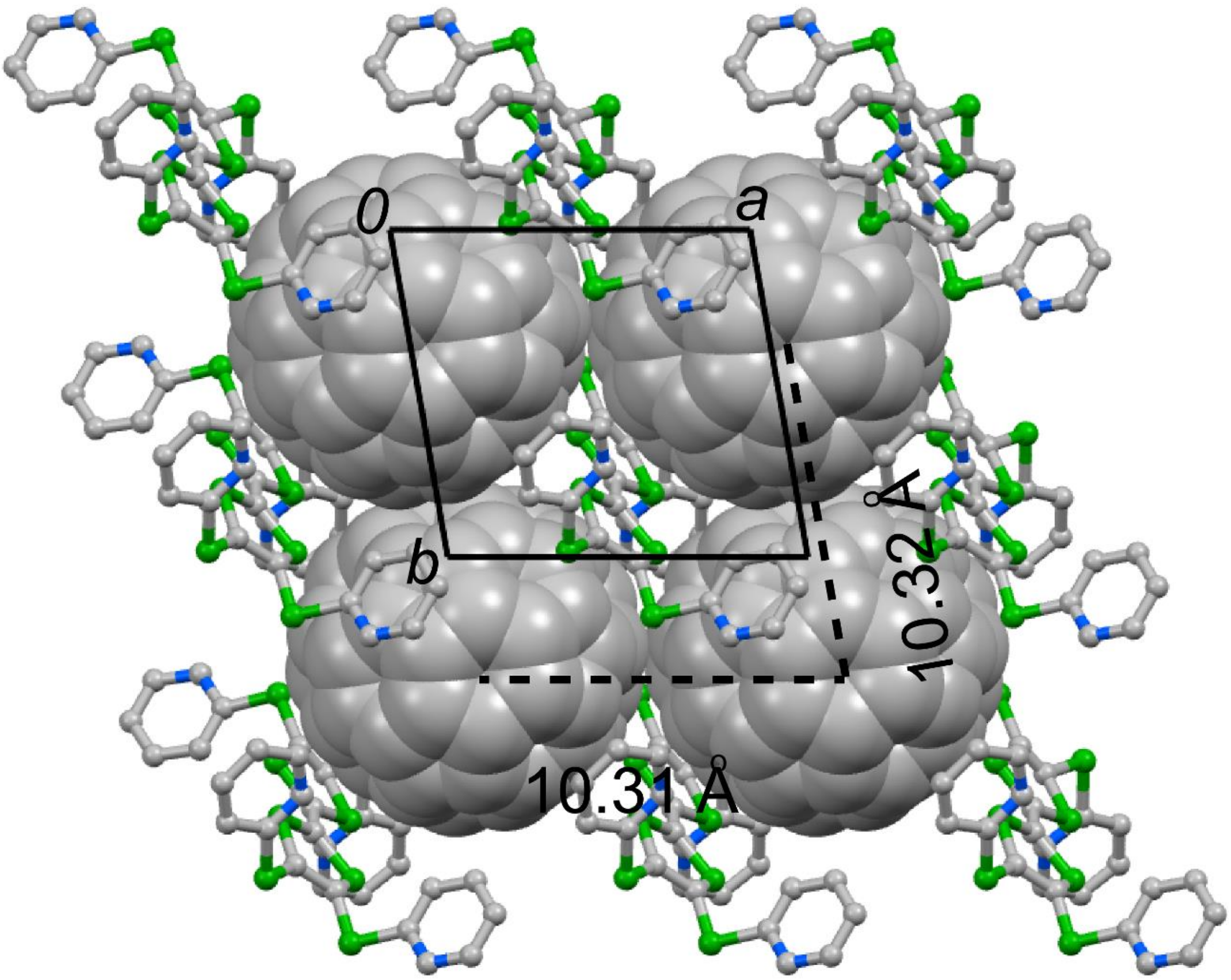

Figure S6: Crystal structure of **7**•C_{60} projected along the crystallographic *c*-axis. The centre-to-centre distances between neighboring C_{60} molecules along the *a*- and *b*-axes are 10.31 and 10.32 Å respectively. The C_{60} molecules are drawn in spacefill style and the hydrogen atoms are omitted for clarity.

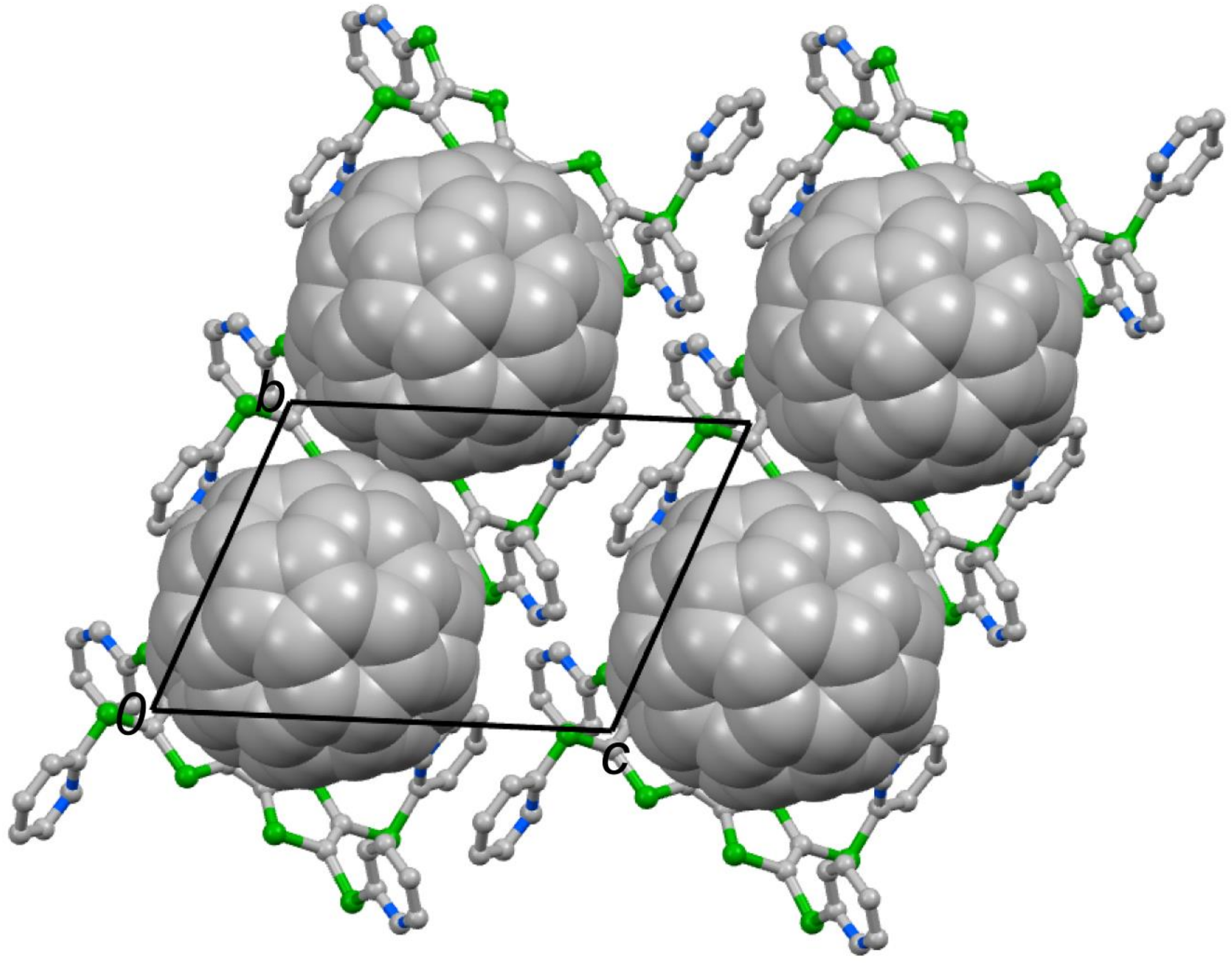

Figure S7: Crystal structure of **7**•C_{60} projected along the crystallographic *a*-axis with hydrogen atoms omitted for clarity. The C_{60} molecules are drawn in spacefill style.

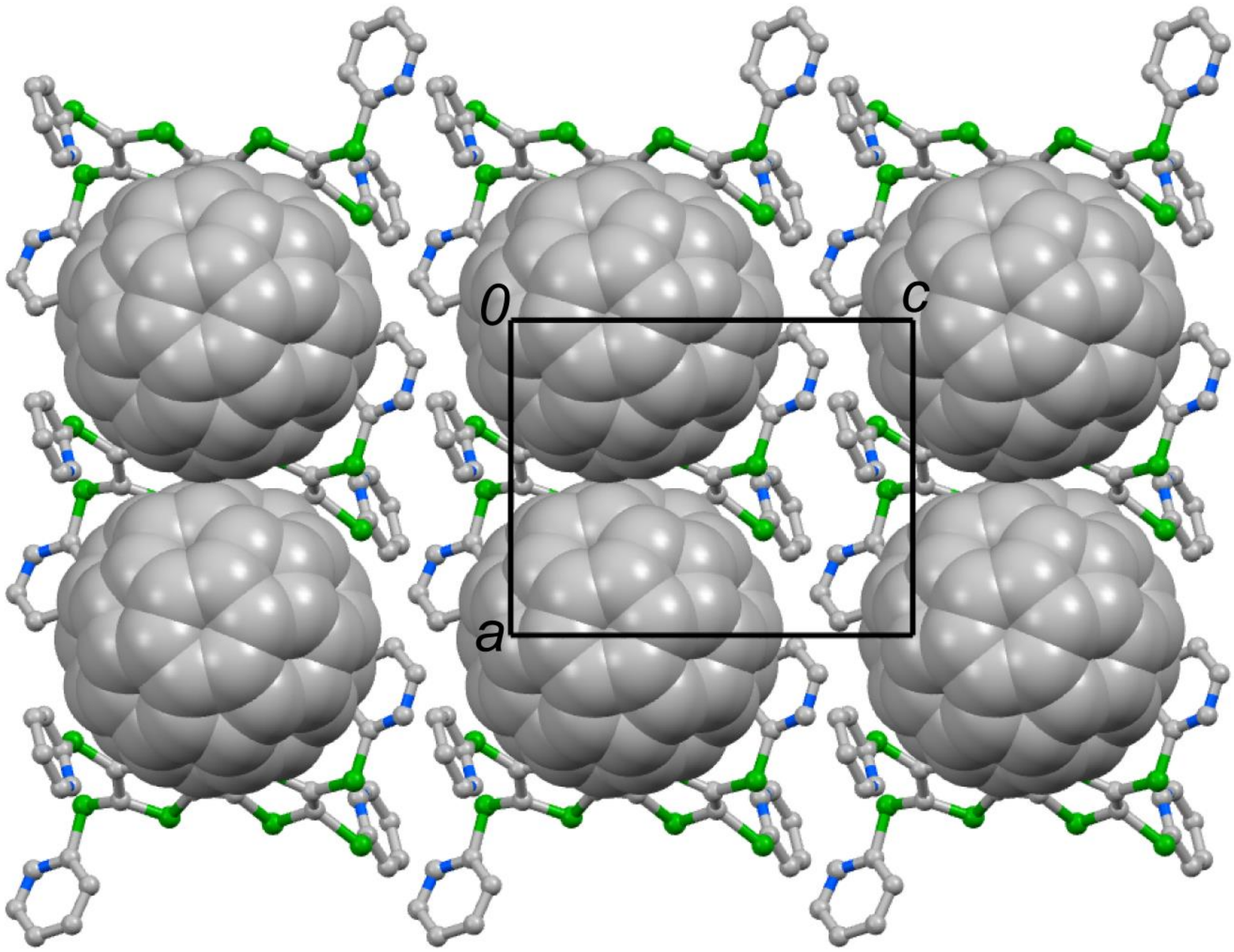

Figure S8: Crystal structure of **7**•C_{60} projected along the crystallographic *b*-axis. The C_{60} molecules are drawn in spacefill style and the hydrogen atoms are omitted for clarity.

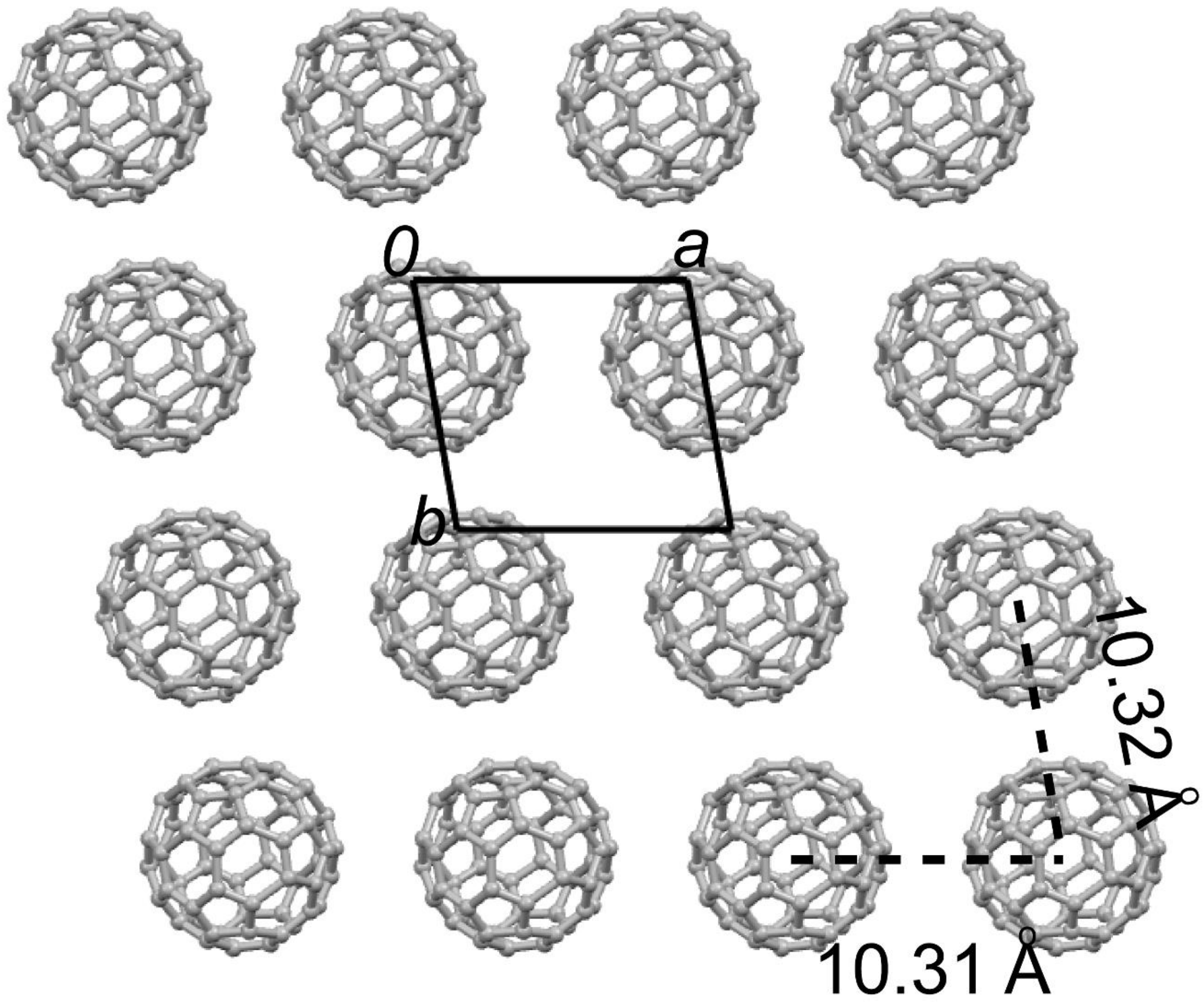

Figure S9: The C_{60} molecules arrangement in the *ab*-plane in complex **7**•C_{60}. The center-to-center distances between C_{60} molecules along *a*- and *b*-axis are 10.32 Å and 10.31 Å, respectively.

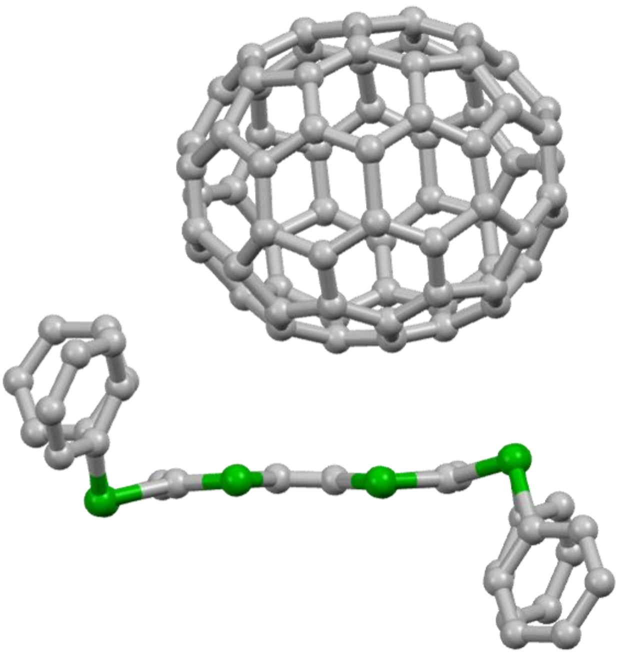

Figure S10: The unit cell contents of **1**•C_{70} viewed along the short axis of **1**. The complex crystallizes in the triclinic space group *P*1, and the asymmetric unit contains one **1** molecule and one C_{70} molecule. The central TTF core on **1** is nearly planar. The long axis of C_{70} and **1** are almost parallel to each other. The hydrogen atoms are omitted for clarity.

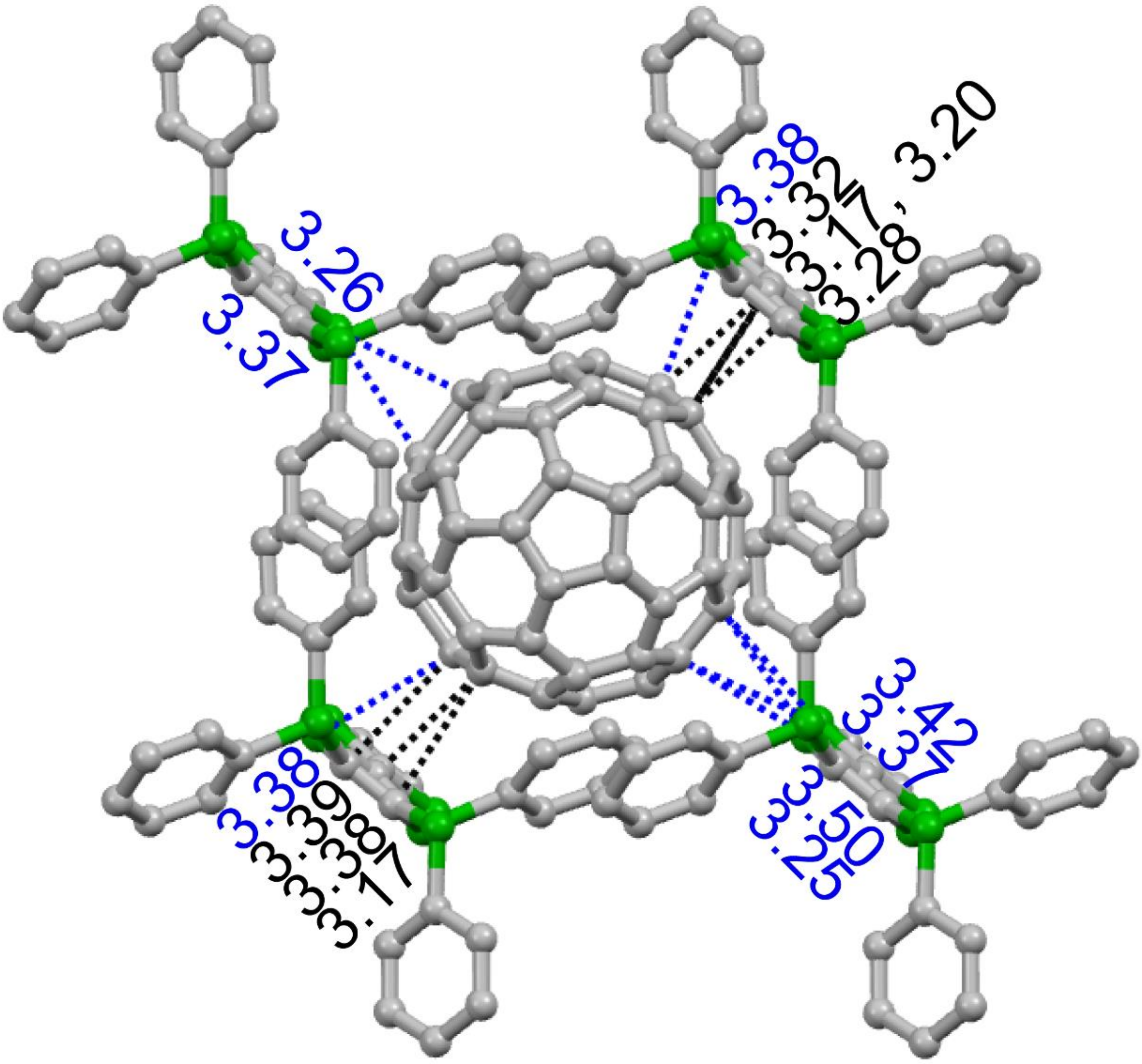

Figure S11: Encapsulation of C_{70} in **1**•C_{70}. The blue and black dashed lines indicate the intermolecular C–S and C–C contacts between the central core of **1** and C_{70}, respectively. The C–C atomic short contacts are also observed between the aryls substituents and C_{70}: 3.22 to 3.36 Å. The grey and green balls represent carbon and sulfur atoms respectively, and the hydrogen atoms are omitted for clarity.

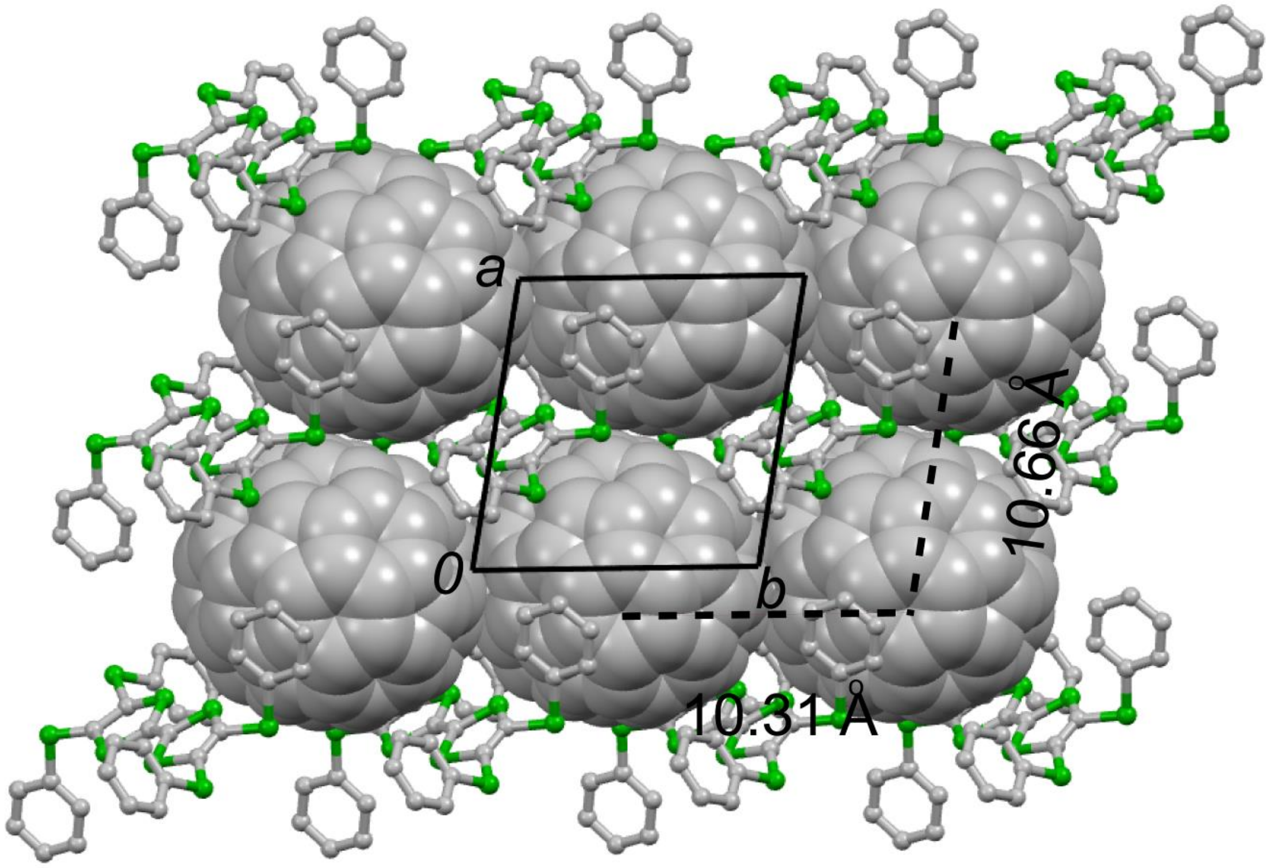

Figure S12: Crystal structure of **1**•C_{70} projected along the crystallographic *c*-axis. The centre-to-centre distances between neighboring C_{70} molecules along the *a*- and *b*-axes are 10.31 and 10.66 Å, respectively. The C_{70} molecules are drawn in spacefill style and the hydrogen atoms are omitted for clarity.

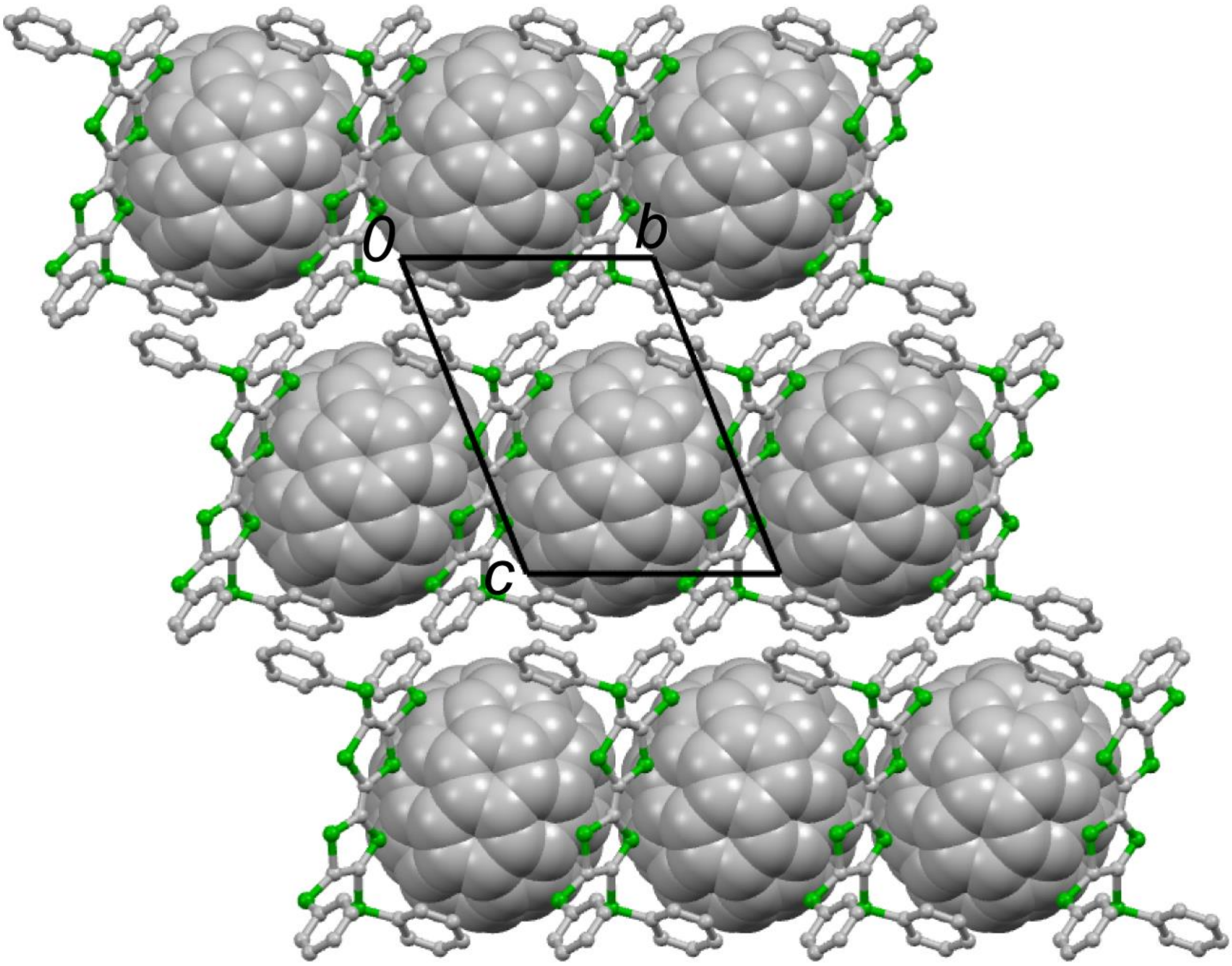

Figure S13: Crystal structure of **1**•C_{70} projected along the crystallographic *a*-axis. The C_{70} molecules are drawn in spacefill style and the hydrogen atoms are omitted for clarity.

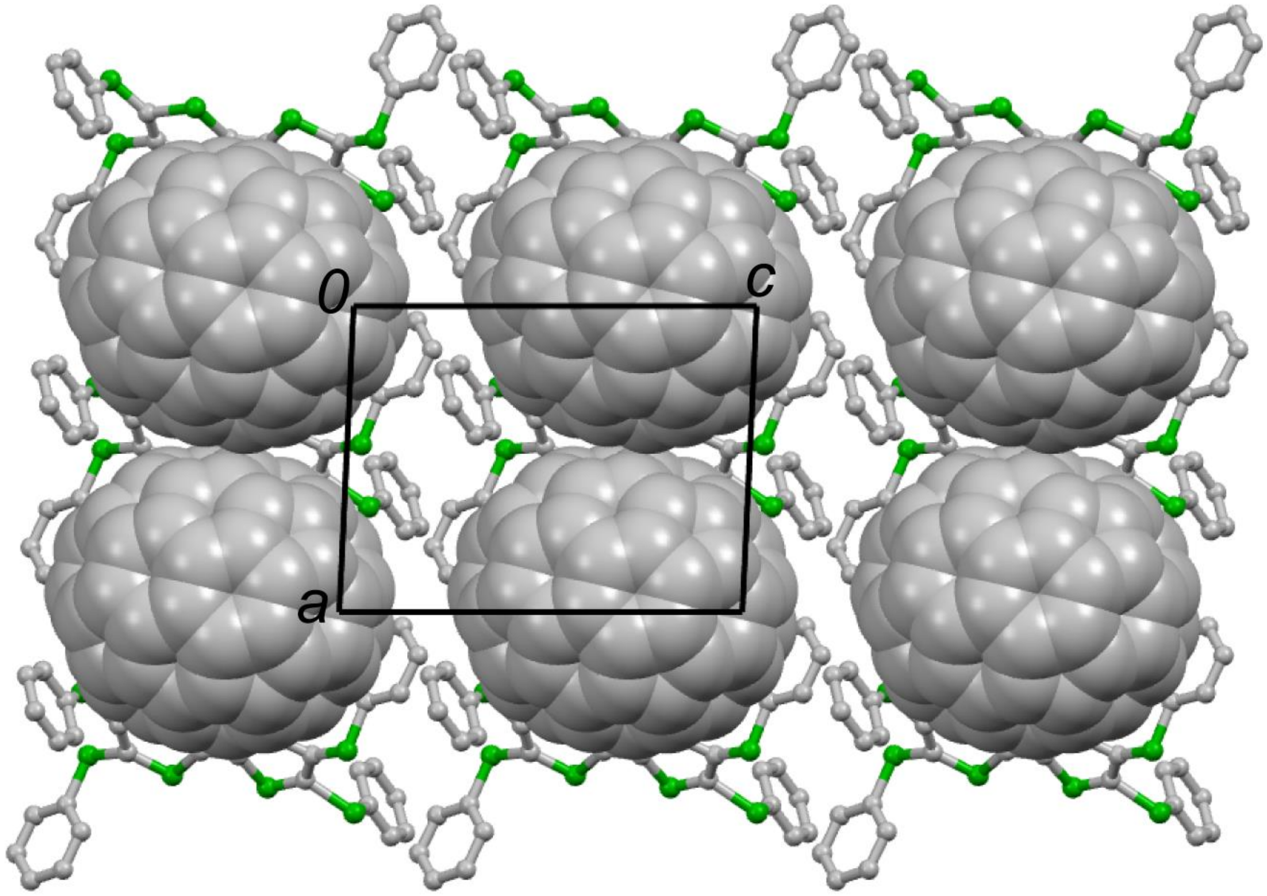

Figure S14: Crystal structure of **1**•C_{70} projected along the crystallographic *b*-axis. The C_{70} molecules are drawn in spacefill style and the hydrogen atoms are omitted for clarity.

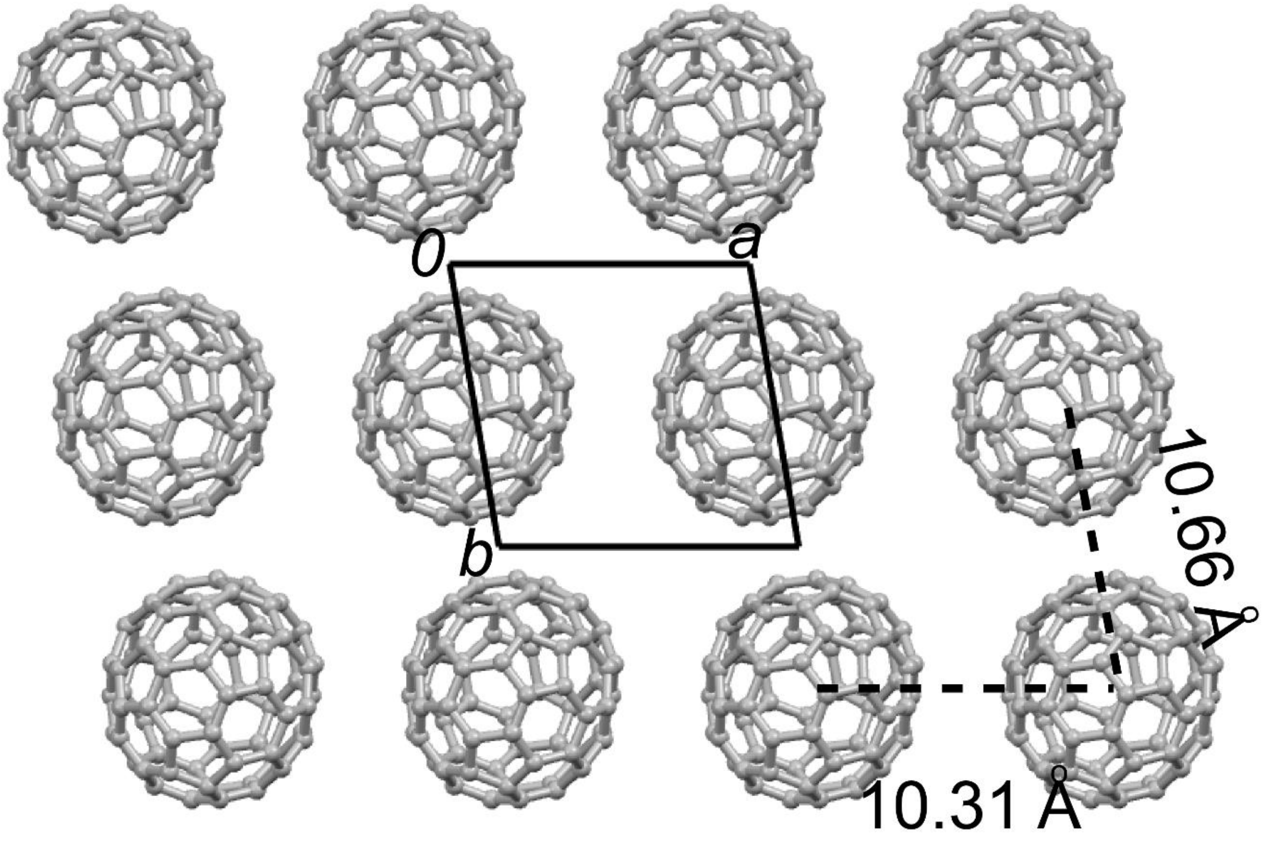

Figure S15: The C_{70} molecules arrangement in the *ab*-plane in complex **1**•C_{70}. The center-to-center distances between C_{70} molecules along *a*- and *b*-axis are 10.31 Å and 10.66 Å, respectively.

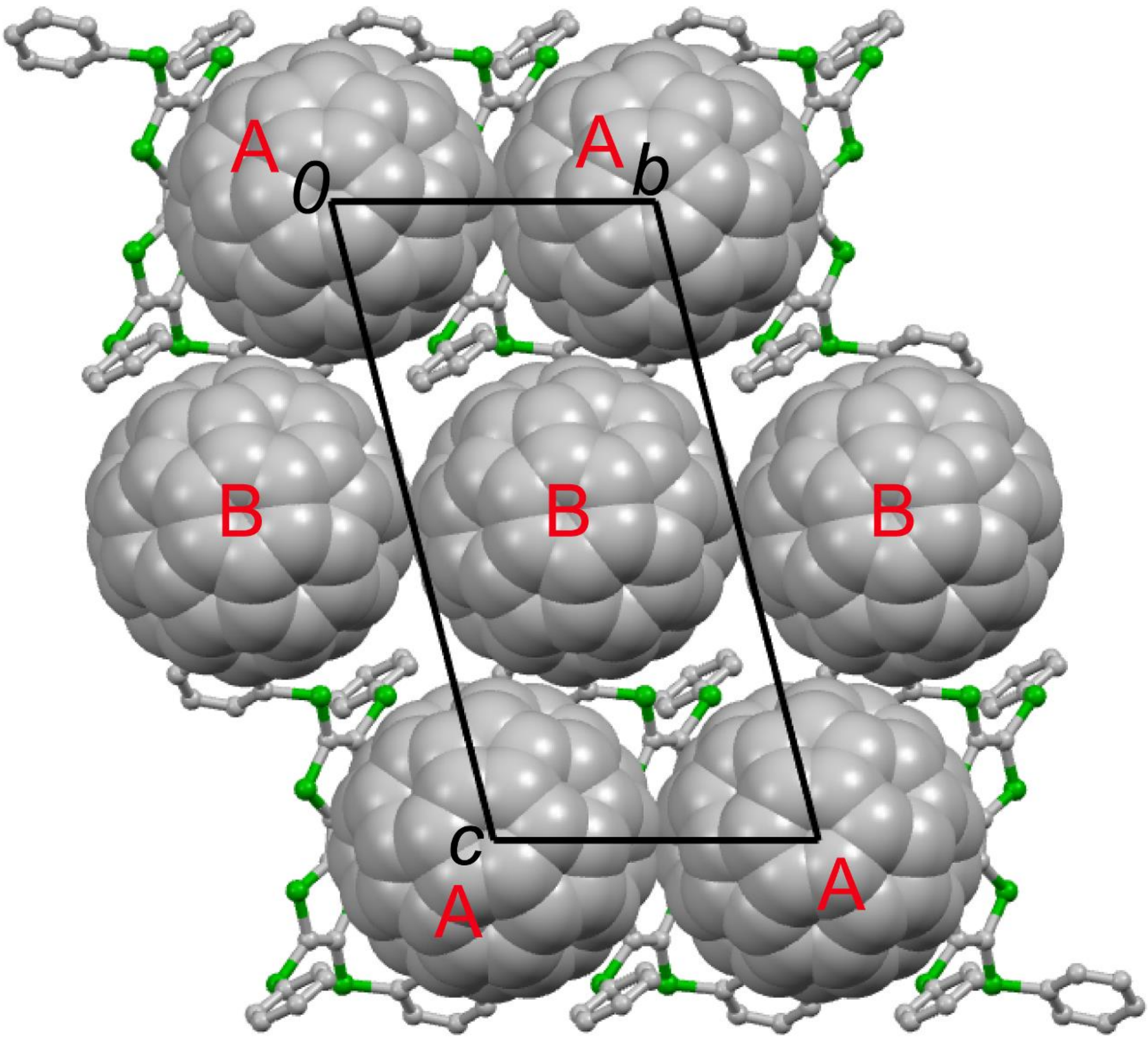

Figure S16: Crystal structure of **1**•$(C_{60})_2$•$(CS_2)_2$ projected along the crystallographic *a*-axis, where ***A*** and ***B*** represent two kinds of C60 molecules as described in the main text. The C_{60} molecules are drawn in spacefill style, and the hydrogen atoms and CS_2 molecules are omitted for clarity.

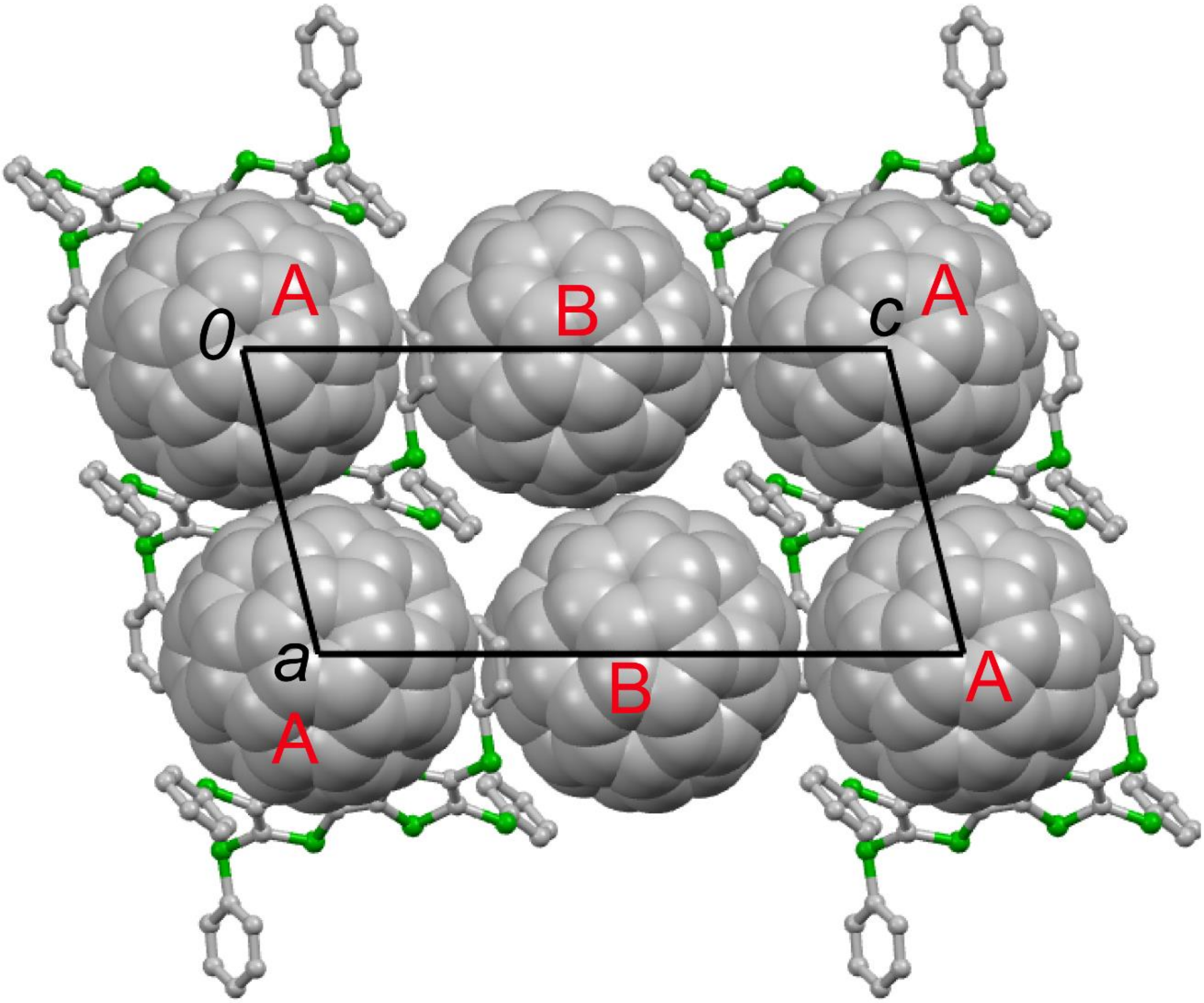

Figure S17: Crystal structure of **1**•$(C_{60})_2$•$(CS_2)_2$ projected along the crystallographic *b*-axis. The C_{60} molecules are drawn in spacefill style and the hydrogen atoms are omitted for clarity.

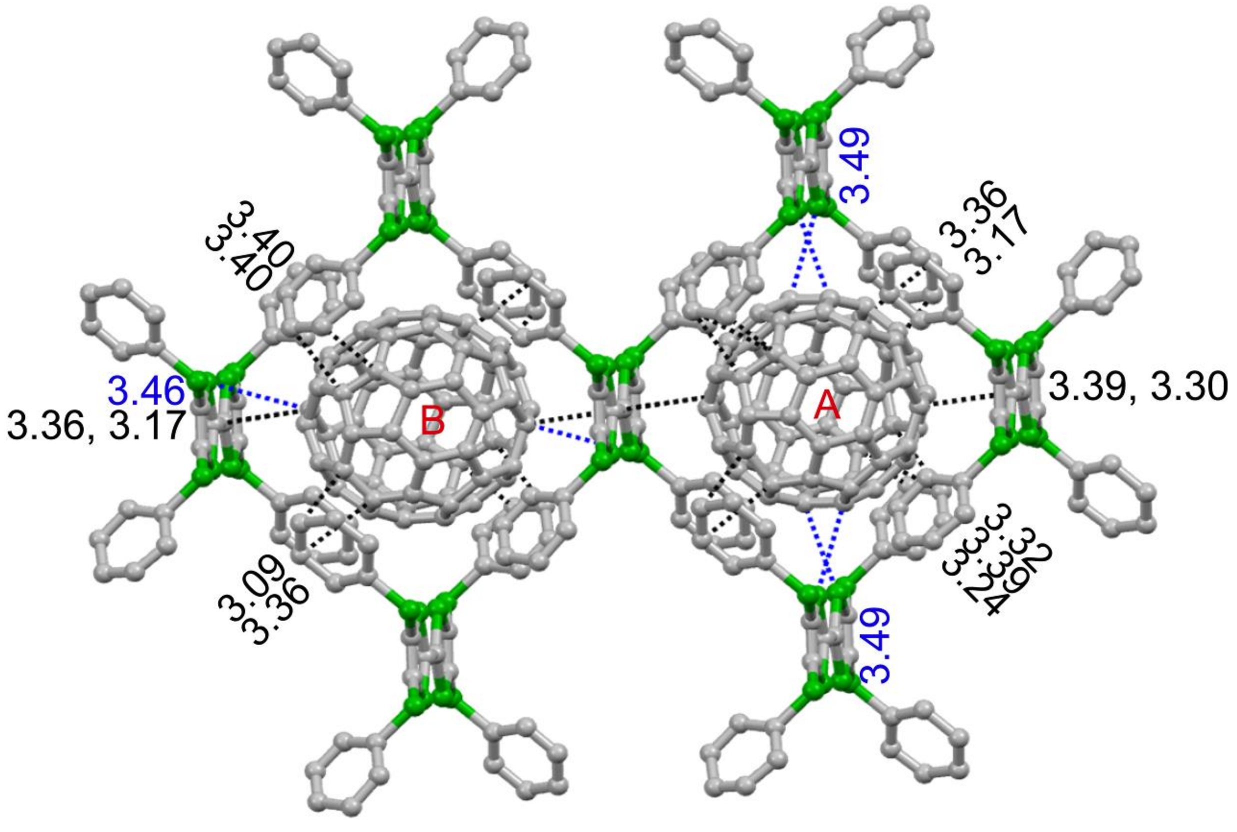

Figure S18: Encapsulation of C_{60} molecules (***A*** and ***B***) by **1** molecules in **1**•$(C_{60})_2$•PhCl. The black and blue dashed lines indicate the intermolecular C–C and C–S atomic short contacts between the **1** and C_{60} respectively. The hydrogen atoms are omitted for clarity.

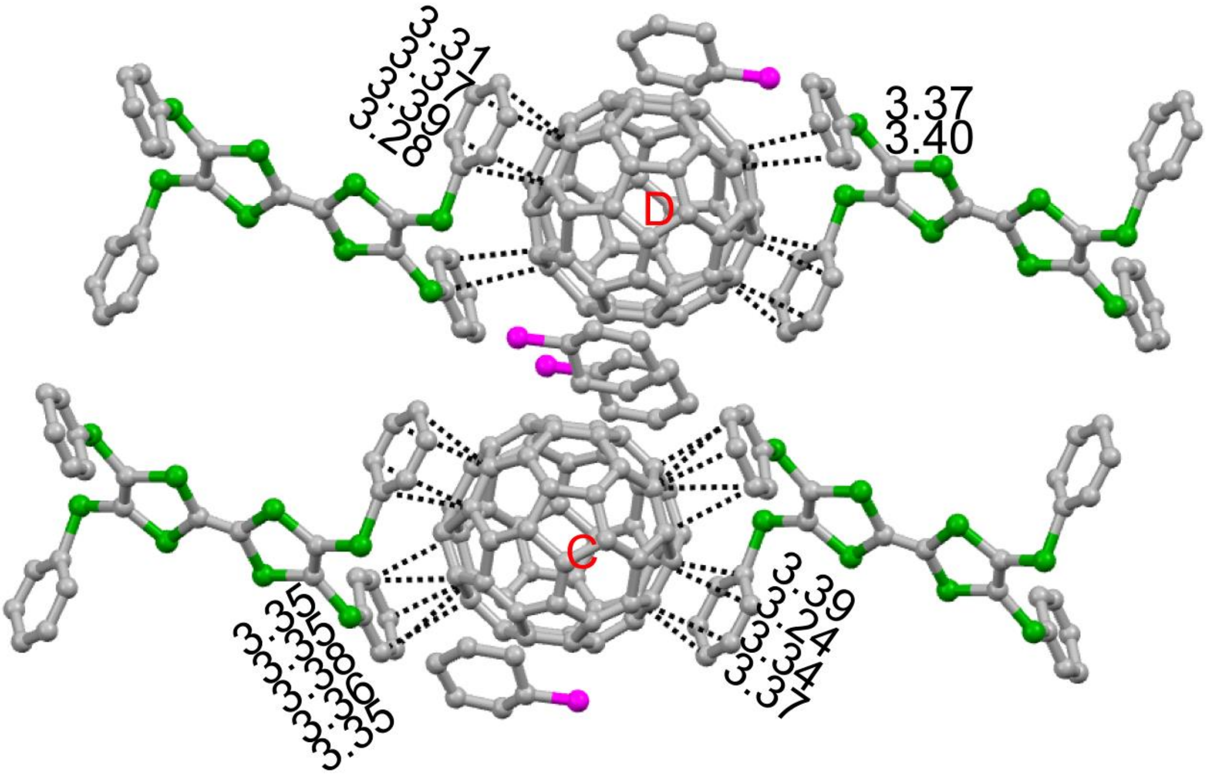

Figure S19: Encapsulation of C_{60} molecules (***C*** and ***D***) by **1** molecules and solvent molecule PhCl in **1**•$(C_{60})_2$•PhCl. The black dashed lines indicate the intermolecular C–C atomic short contacts between the **1** and C_{60}. The atomic close contacts were also observed between the PhCl molecules and C_{60} molecules. For molecule ***C***, short contacts with PhCl molecules are observed: two C–C contacts (3.30–3.35 Å). For molecule ***D***, short contacts between it and PhCl molecules are observed: four C–C contacts (2.88–3.33 Å). The hydrogen atoms are omitted for clarity.

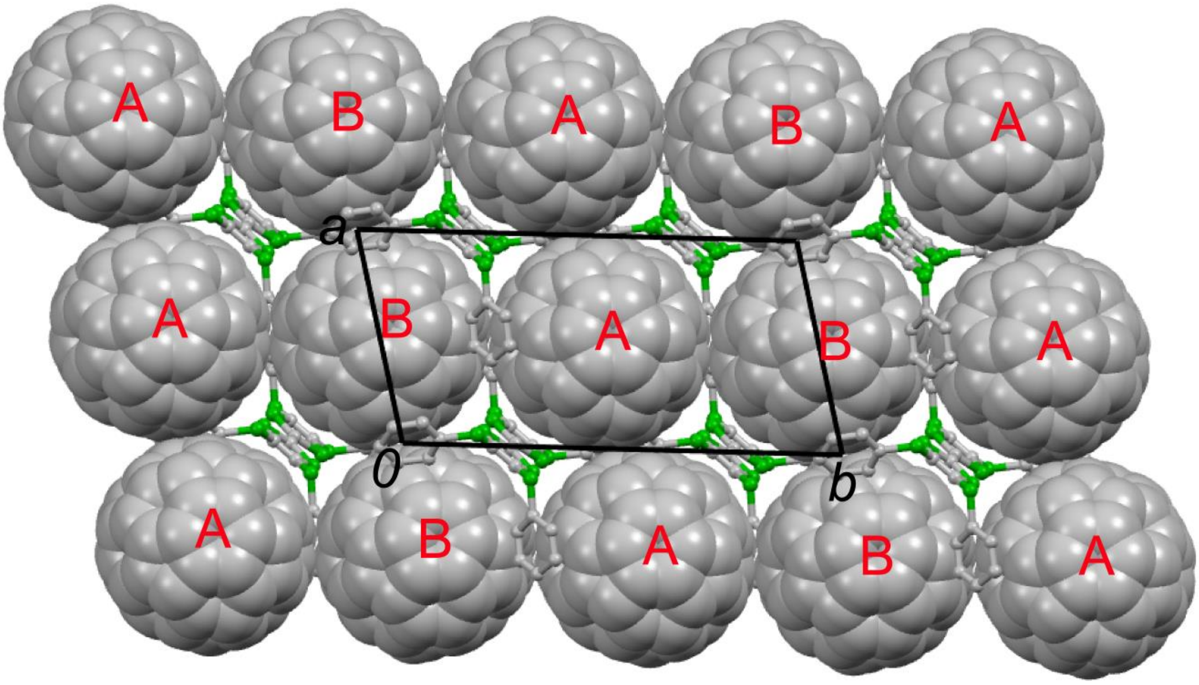

Figure S20: Crystal structure of **1**•$(C_{60})_2$•PhCl projected along the crystallographic *c*-axis. The C_{60} molecules are drawn in spacefill style, and the hydrogen atoms and solvent molecules are omitted for clarity.

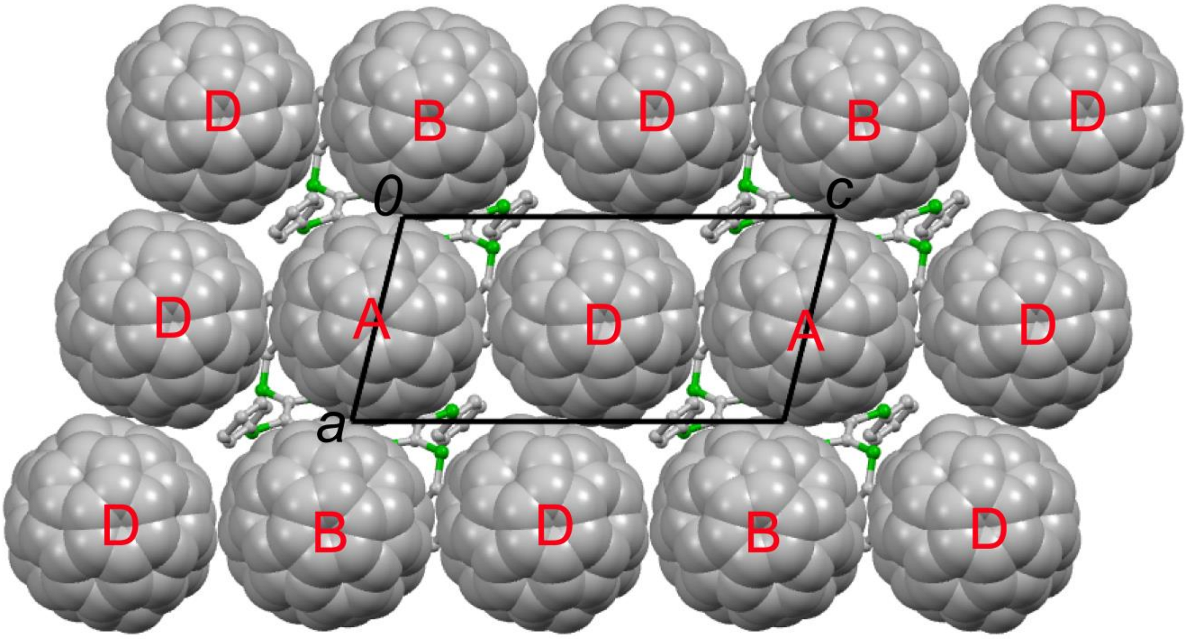

Figure S21: Crystal structure of **1**•$(C_{60})_2$•PhCl projected along the crystallographic *b*-axis. The C_{60} molecules are drawn in spacefill style, and the hydrogen atoms and solvent molecules are omitted for clarity.

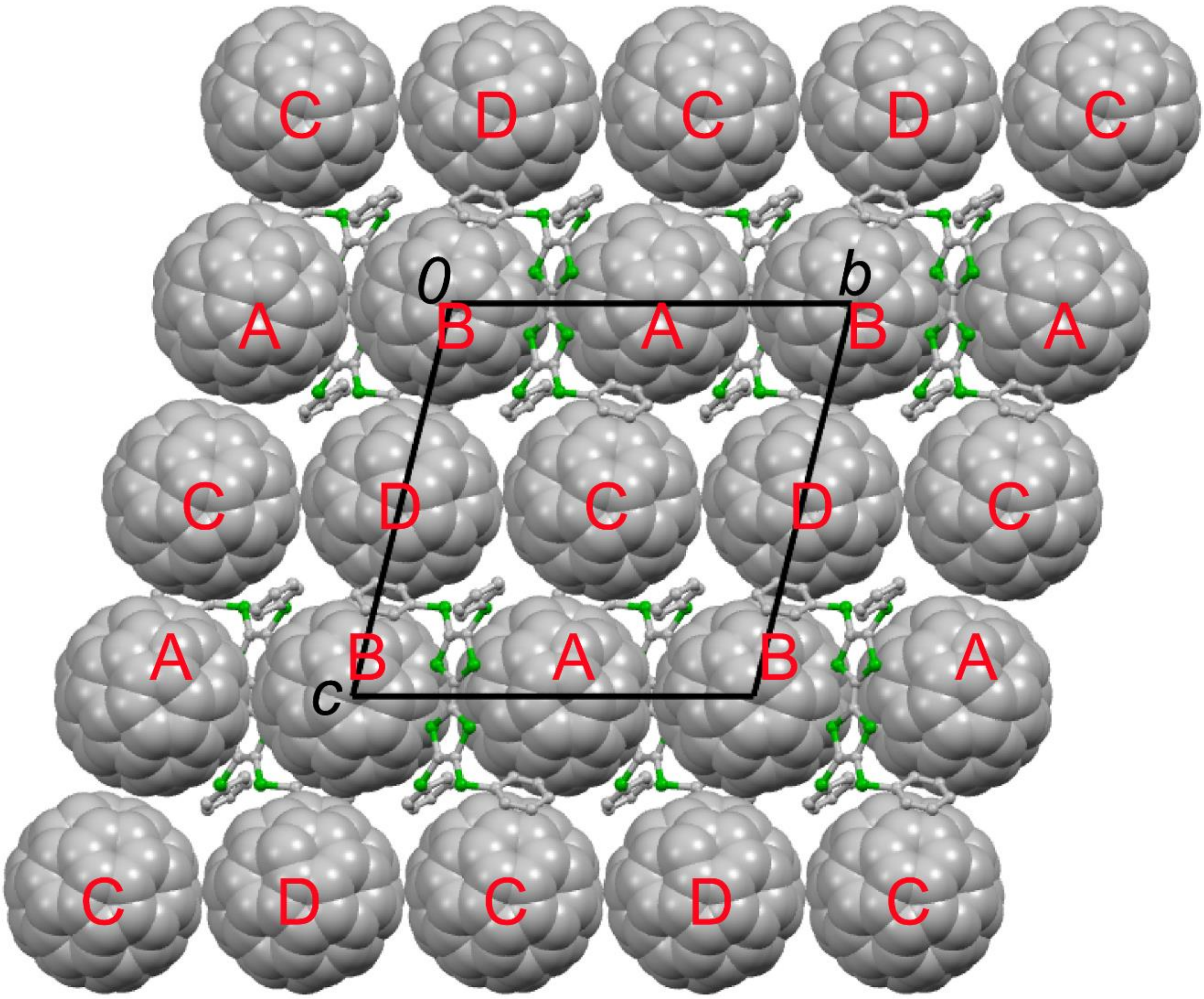

Figure S22: Crystal structure of **1**•$(C_{60})_2$•PhCl projected along the crystallographic *a*-axis. The C_{60} molecules are drawn in spacefill style, and the hydrogen atoms and solvent molecules are omitted for clarity.

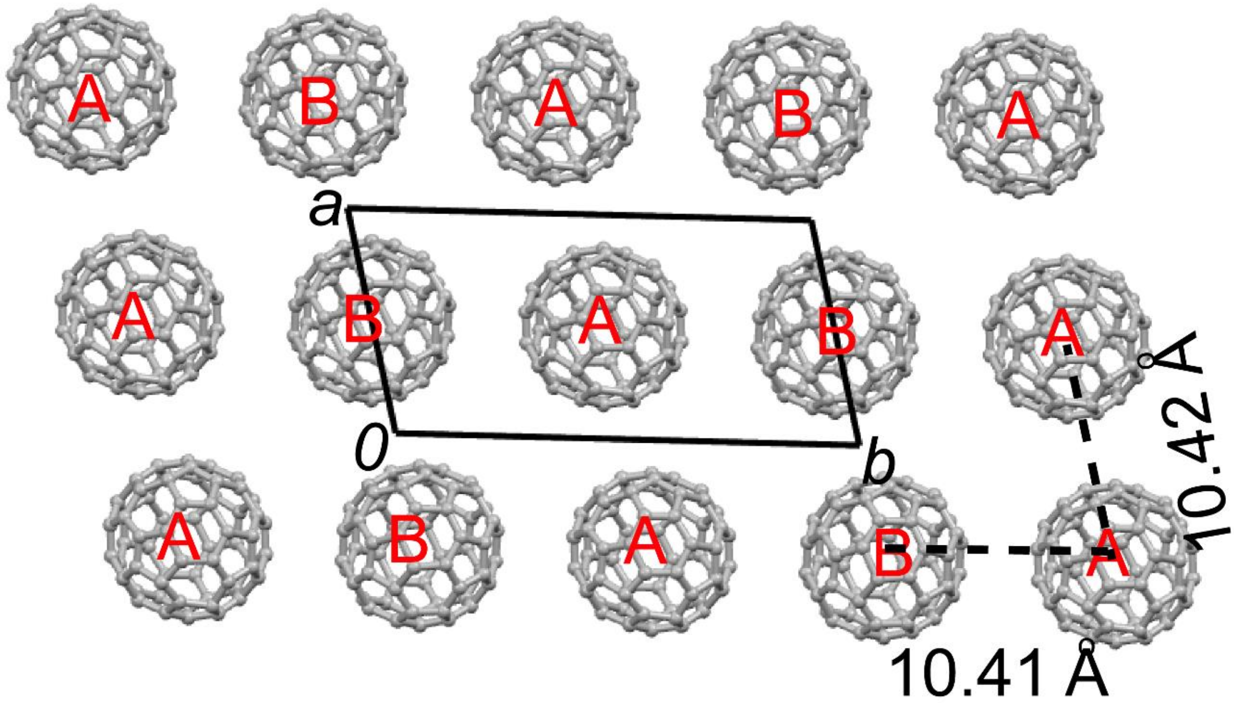

Figure S23: Arrangement of C_{60} molecules ***A*** and ***B*** in the *ab*-plane in complex **1**•$(C_{60})_2$•PhCl.

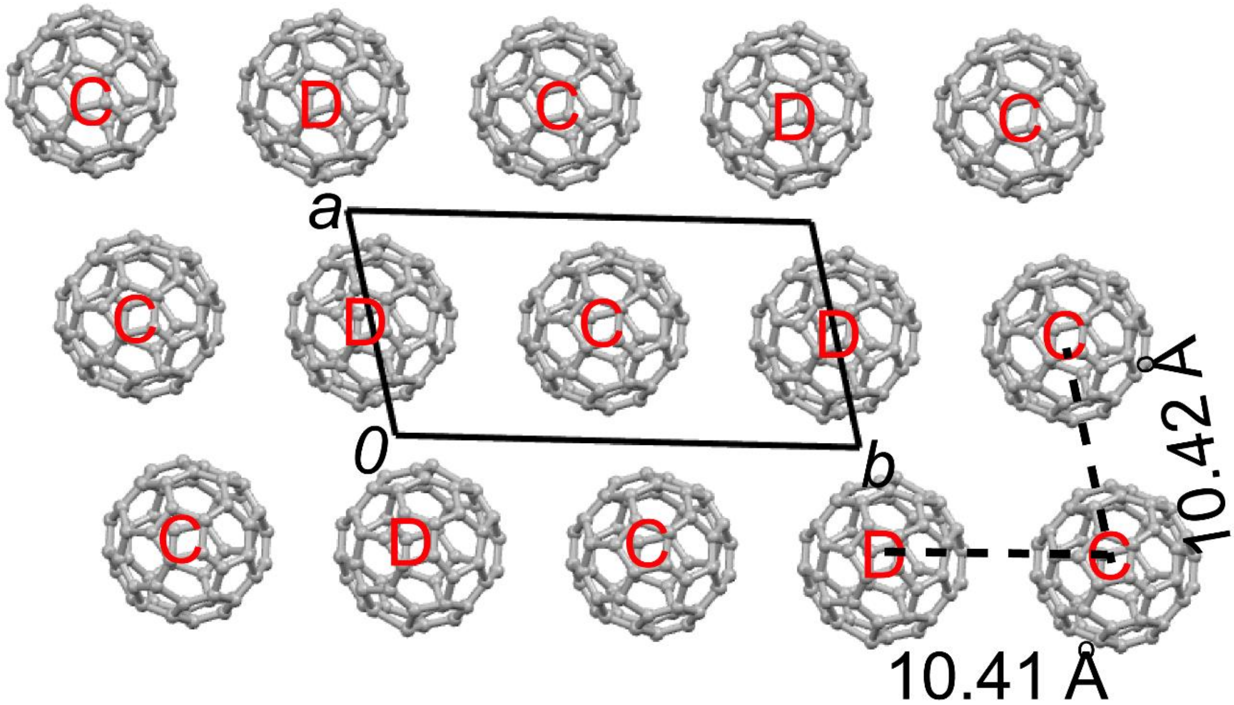

Figure S24: Arrangement of C_{60} molecules ***C*** and ***D*** in the *ab*-plane in complex **1**•$(C_{60})_2$•PhCl.

6•(C_{60})$_2$•(PhCl)$_2$ crystallizes in the triclinic space group *P*-1, and the asymmetric unit contains one molecule **6**, two C_{60} molecules and two PhCl molecules. The central TTF core on **6** is in boat conformation.

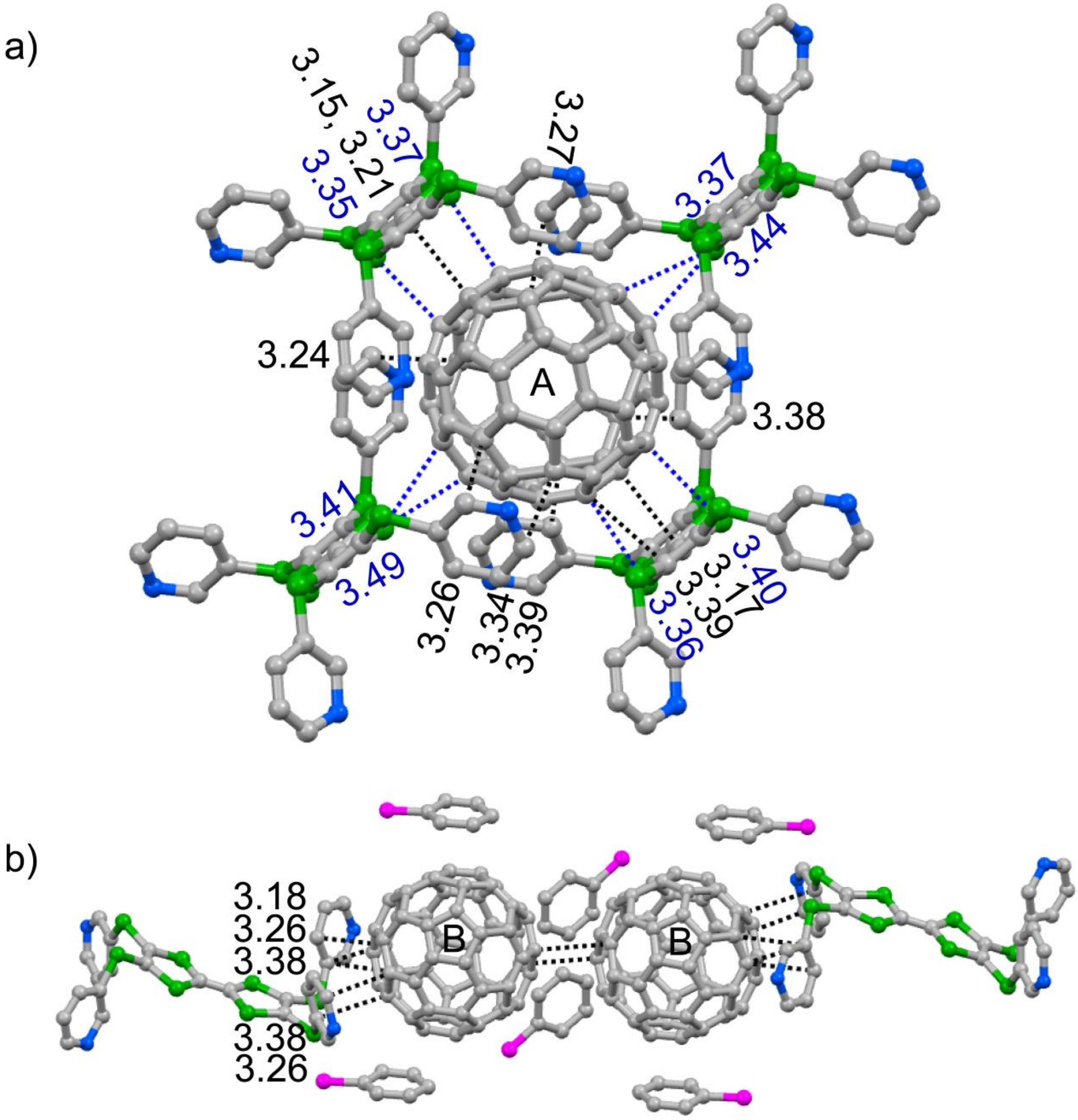

Figure S25: Encapsulation of C_{60} molecules (**A** and **B**) by **6** and solvent PhCl in **6**•(C_{60})$_2$•(PhCl)$_2$. a) ***A*** is encapsulated by four molecules **6**; b) two **B** molecules acted as dimer through two C–C interactions (3.36 Å) and the dimer is surrounded by two **6** and six PhCl molecules. The blue and black dashed lines indicated the intermolecular C–S and C–C contacts between **6** and C_{60} in **6**•(C_{60})$_2$•(PhCl)$_2$, respectively. For molecule **B**, short contacts with PhCl molecules are observed: fourteen C–C contacts (3.20 to 3.38 Å). The hydrogen atoms are omitted for clarity.

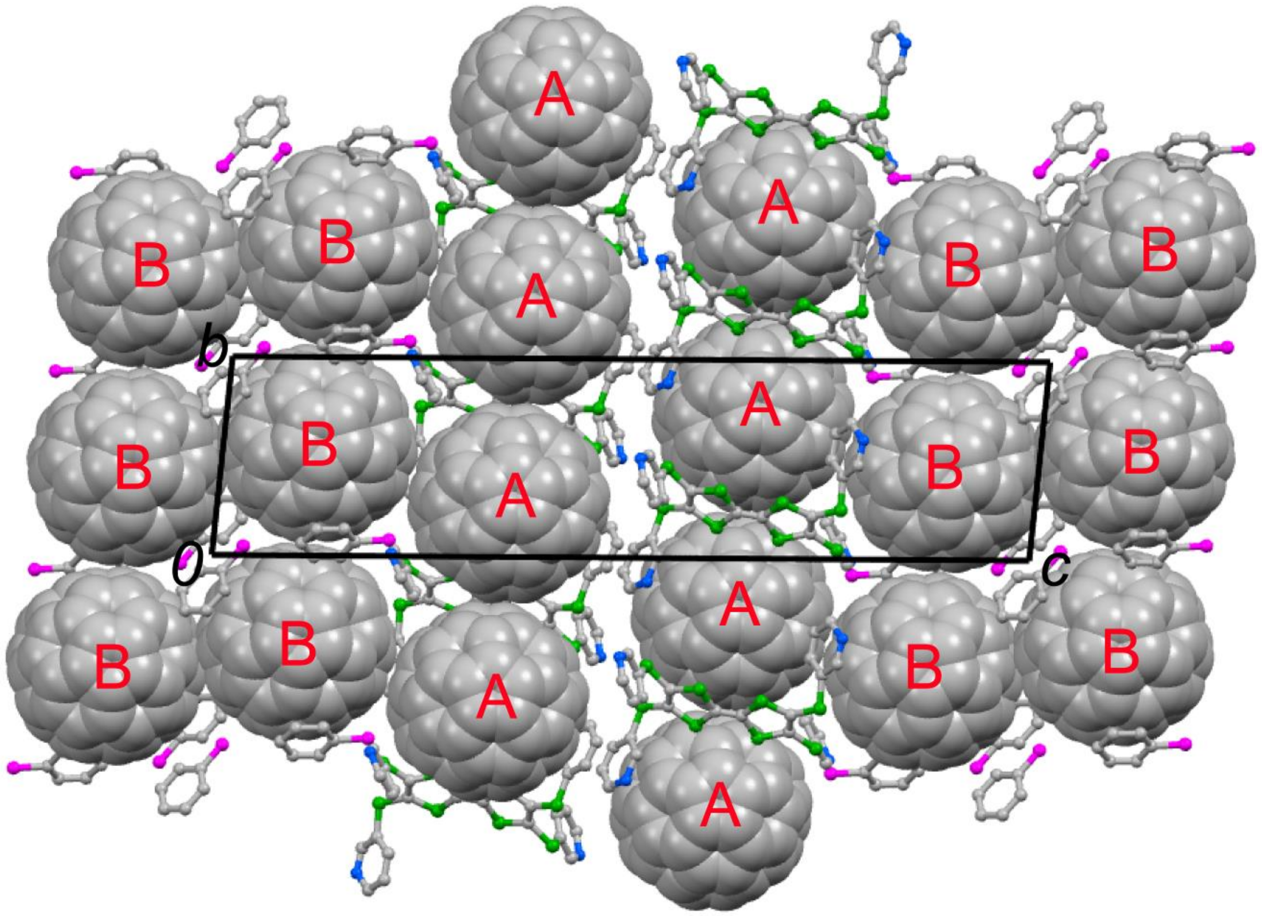

Figure S26: Crystal structure of **6**•$(C_{60})_2$•$(PhCl)_2$ projected along the crystallographic *a*-axis. The C_{60} molecules are drawn in spacefill style and the hydrogen atoms are omitted for clarity.

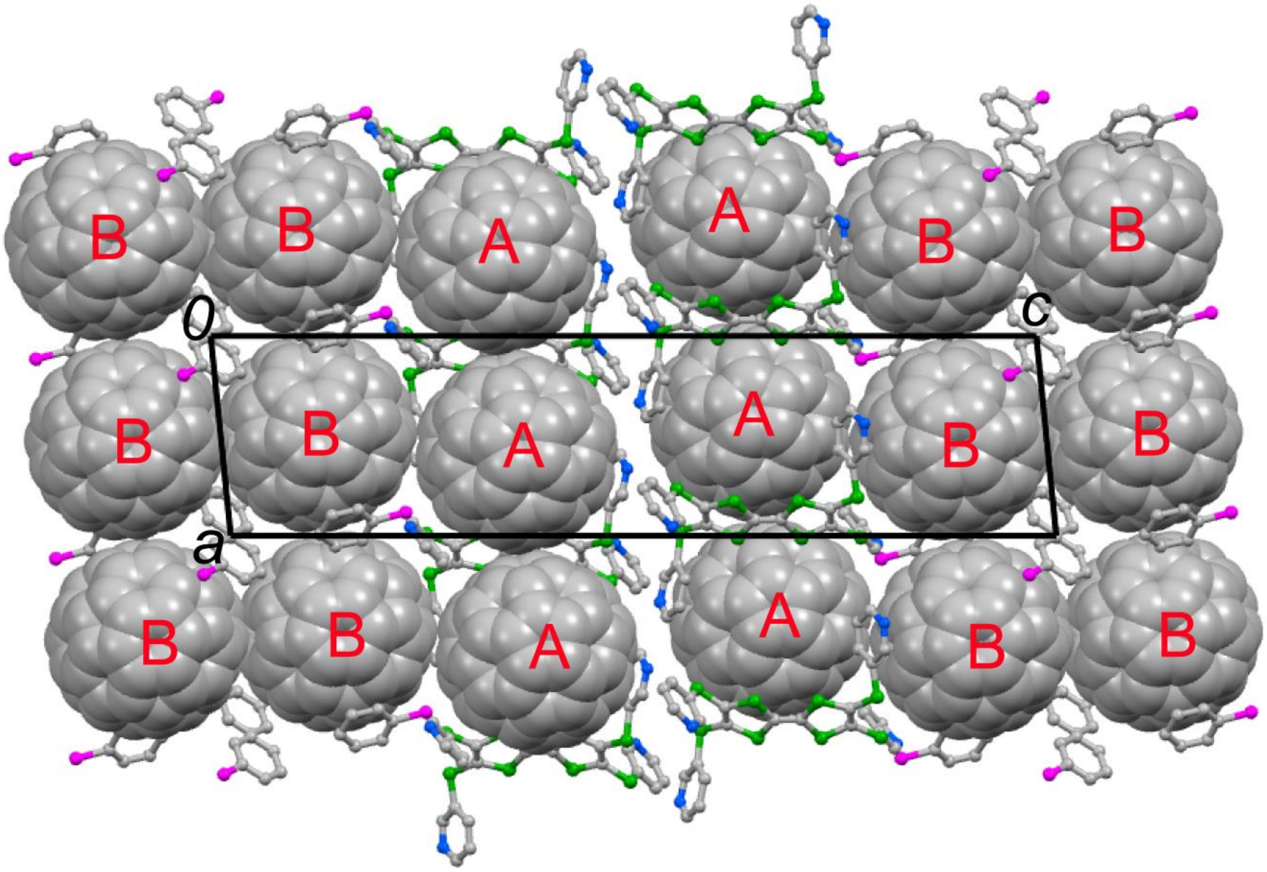

Figure S27: Crystal structure of **6**•$(C_{60})_2$•$(PhCl)_2$ projected along the crystallographic *b*-axis. The C_{60} molecules are drawn in spacefill style and the hydrogen atoms are omitted for clarity.

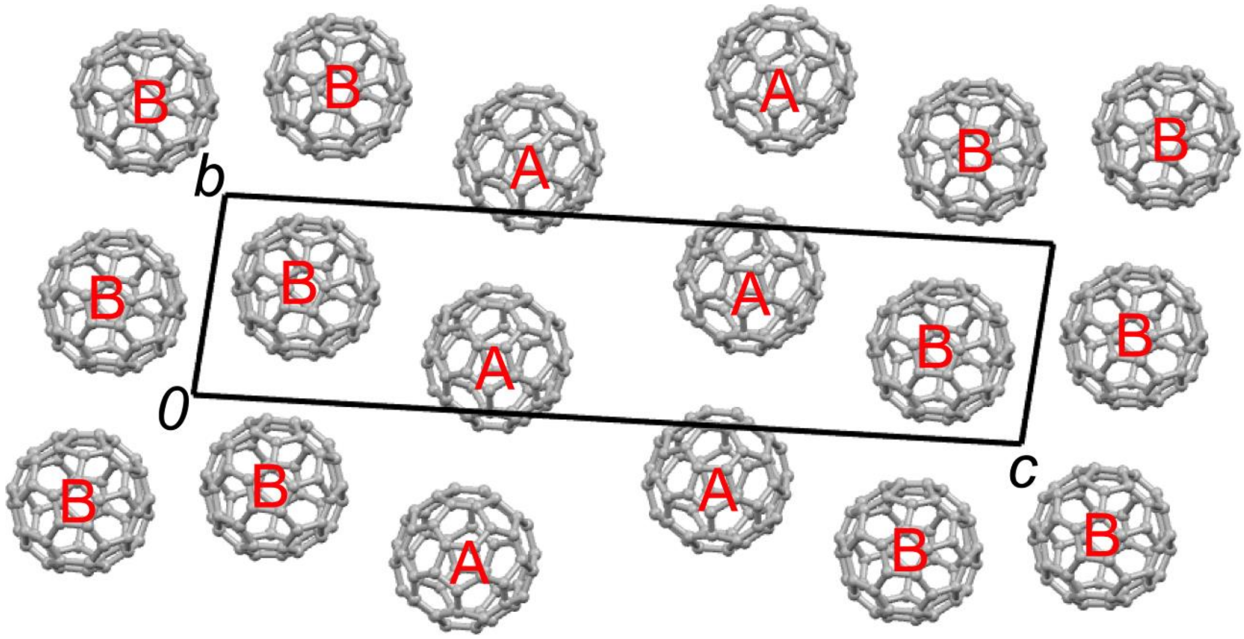

Figure S28: The C_{60} molecules arrange in the *bc*-plane in complex **6**•$(C_{60})_2$•$(PhCl)_2$.

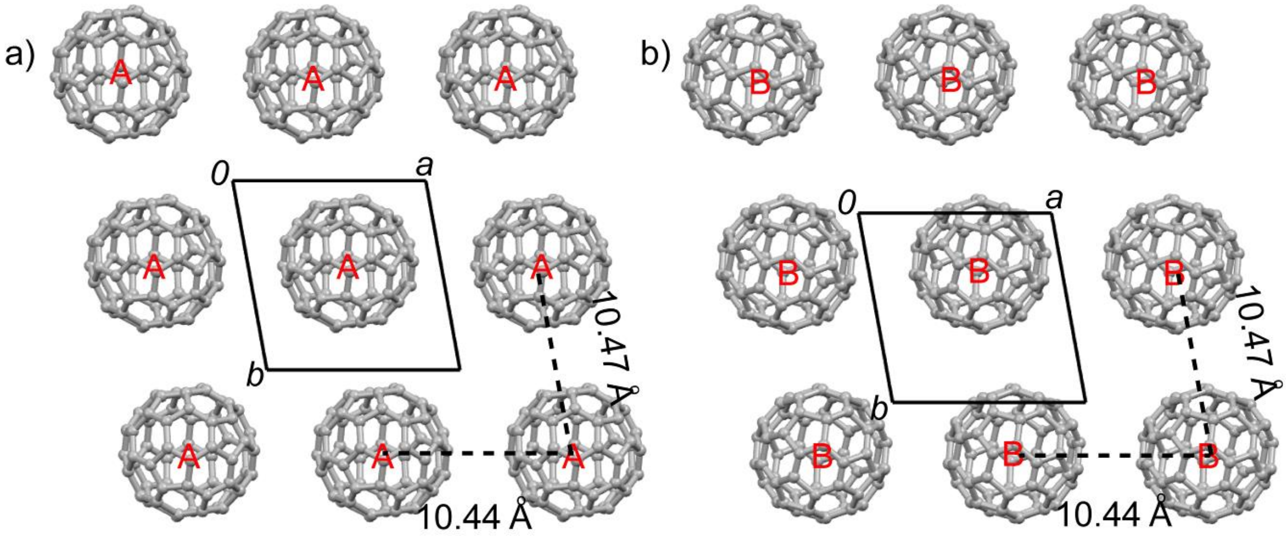

Figure S29: The C_{60} molecules arrange in the *ab*-plane in complex **6**•$(C_{60})_2$•$(PhCl)_2$. a) The **A** on the *ab*-plane; b) the **B** on the *ab*-plane.

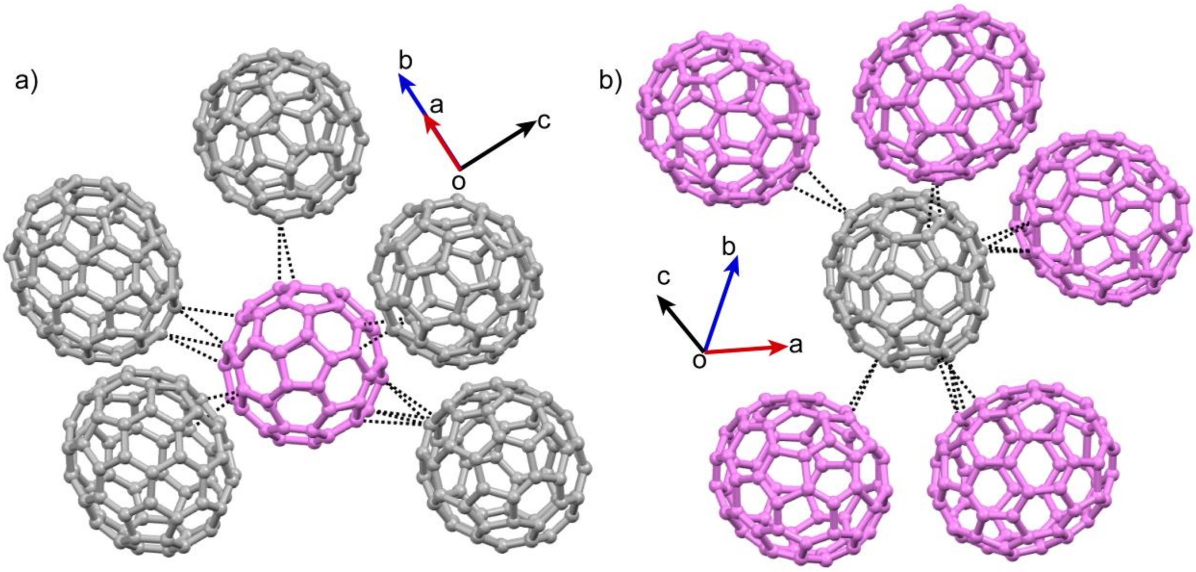

Figure S30: The C_{70} molecules are arranged as three-dimensional network structure in **2**·$(C_{70})_4$·$(PhCl)_2$. In order to explained that, we put **A** and **B** (violet and grey, respectively) in the core and discuss the arrangement of **B** and **A**, respectively. The adjacent C_{70} molecules are distributed as slant four cones. a) the C–C contacts between core **A** and adjacent **B** are range from 3.20 to 3.38 Å; b) the distances of contacts between core **B** and adjacent **A** are same as a).

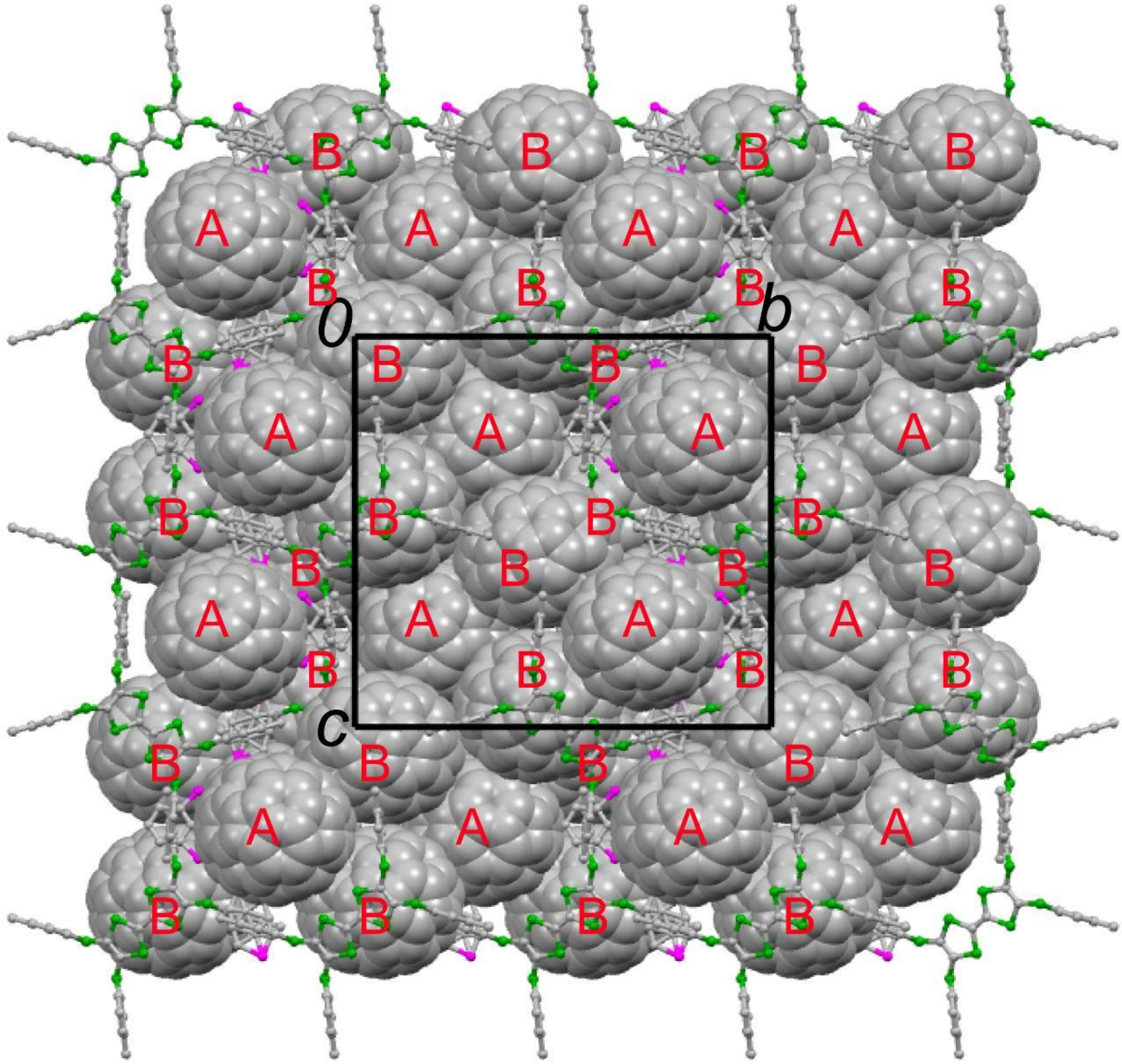

Figure S31: Crystal structure of **2**•$(C_{70})_4$•$(PhCl)_2$ projected along the crystallographic *a*-axis. The C_{70} molecules are drawn in spacefill style and the hydrogen atoms are omitted for clarity.

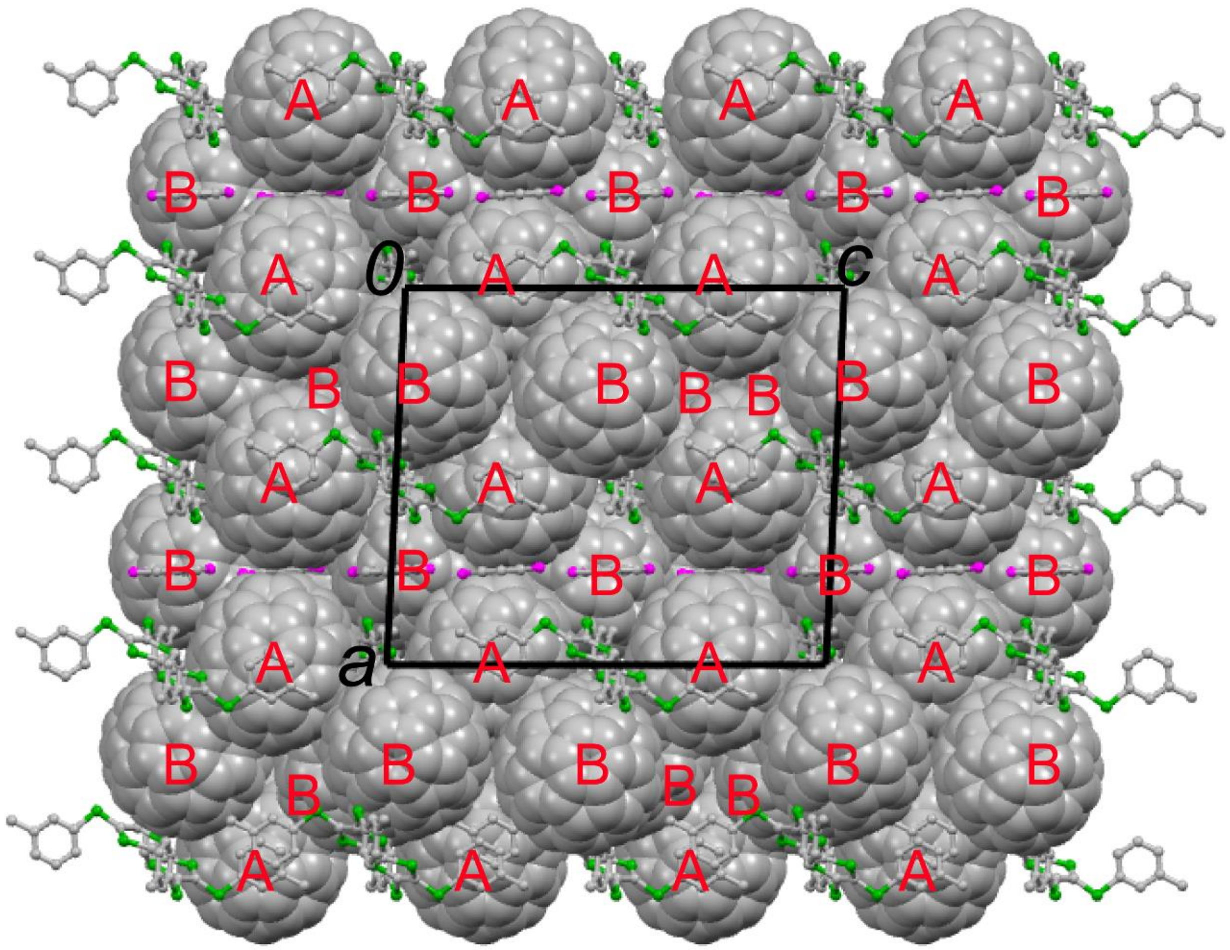

Figure S32: Crystal structure of **2**•$(C_{70})_4$•$(PhCl)_2$ projected along the crystallographic *b*-axis. The C_{70} molecules are drawn in spacefill style and the hydrogen atoms are omitted for clarity.

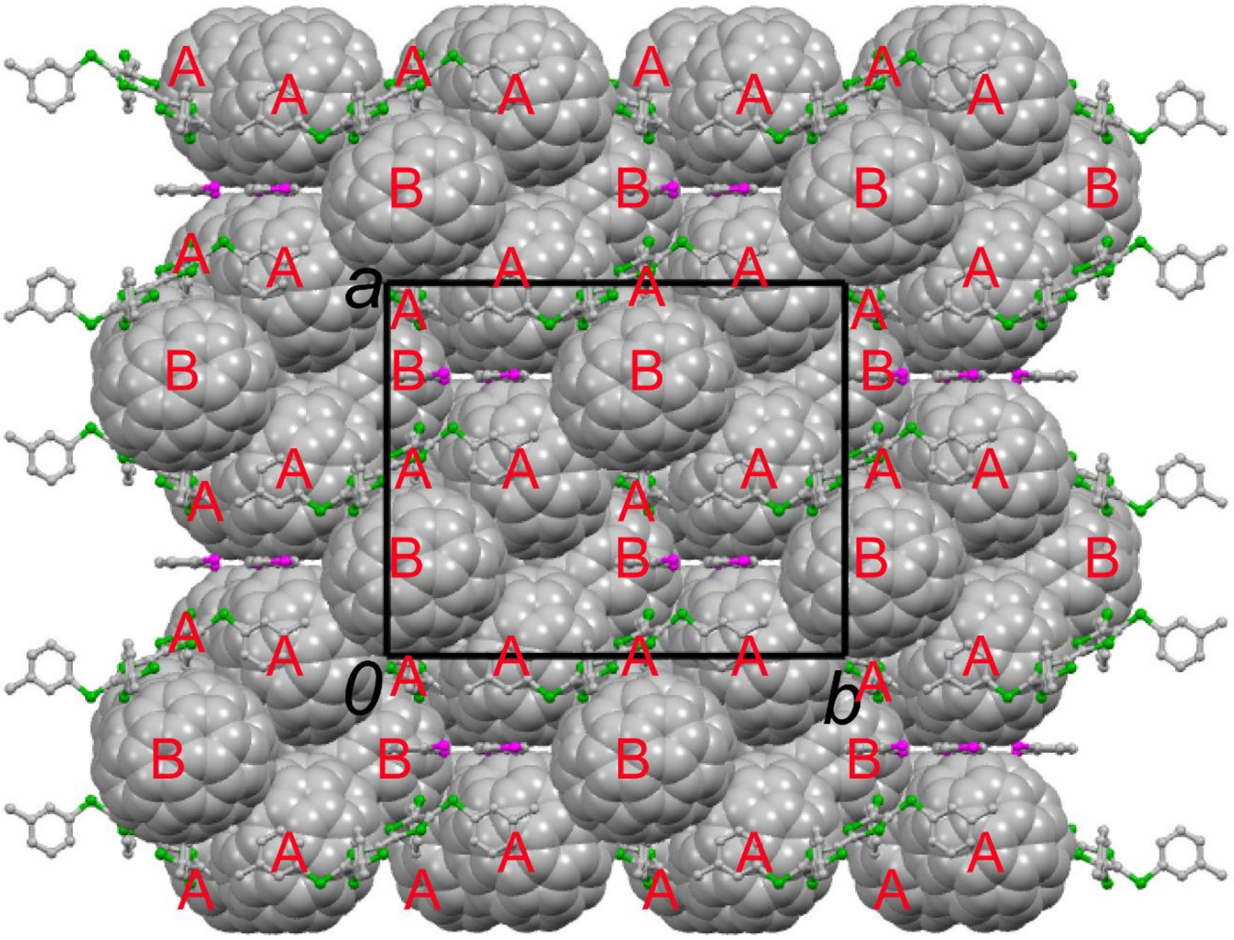

Figure S33: Crystal structure of **2**•$(C_{70})_4$•$(PhCl)_2$ projected along the crystallographic *c*-axis. The C_{70} molecules are drawn in spacefill style and the hydrogen atoms are omitted for clarity.

IR Spectra

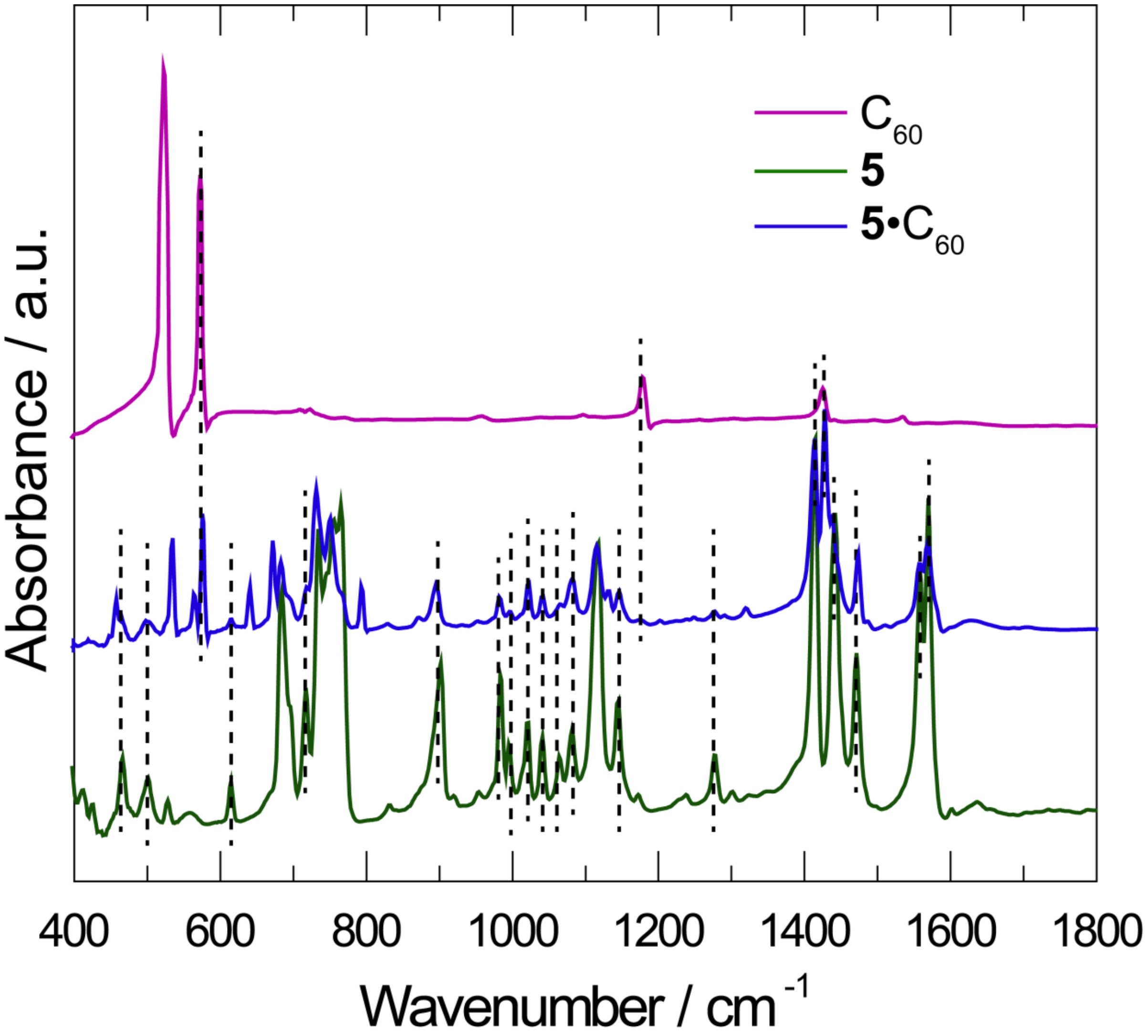

Figure S34: IR spectra of **5**•C_{70} along with those of **5** and C_{70} for comparison (in KBr). The black dashed lines are guides for the eye.

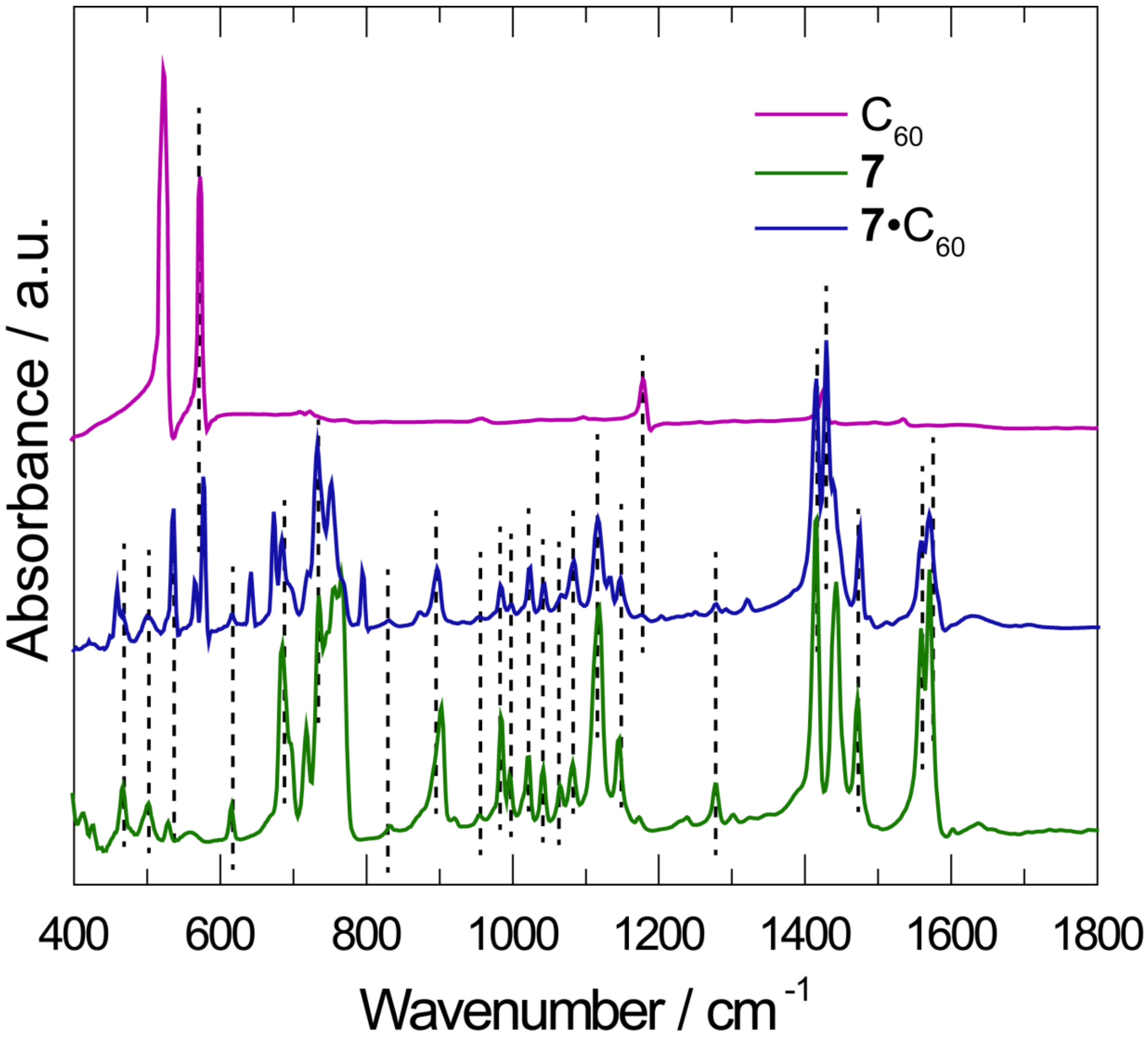

Figure S35: IR spectra of **7**•C_{60} along with those of **7** and C_{60} for comparison (in KBr). The black dashed lines are guides for the eye.

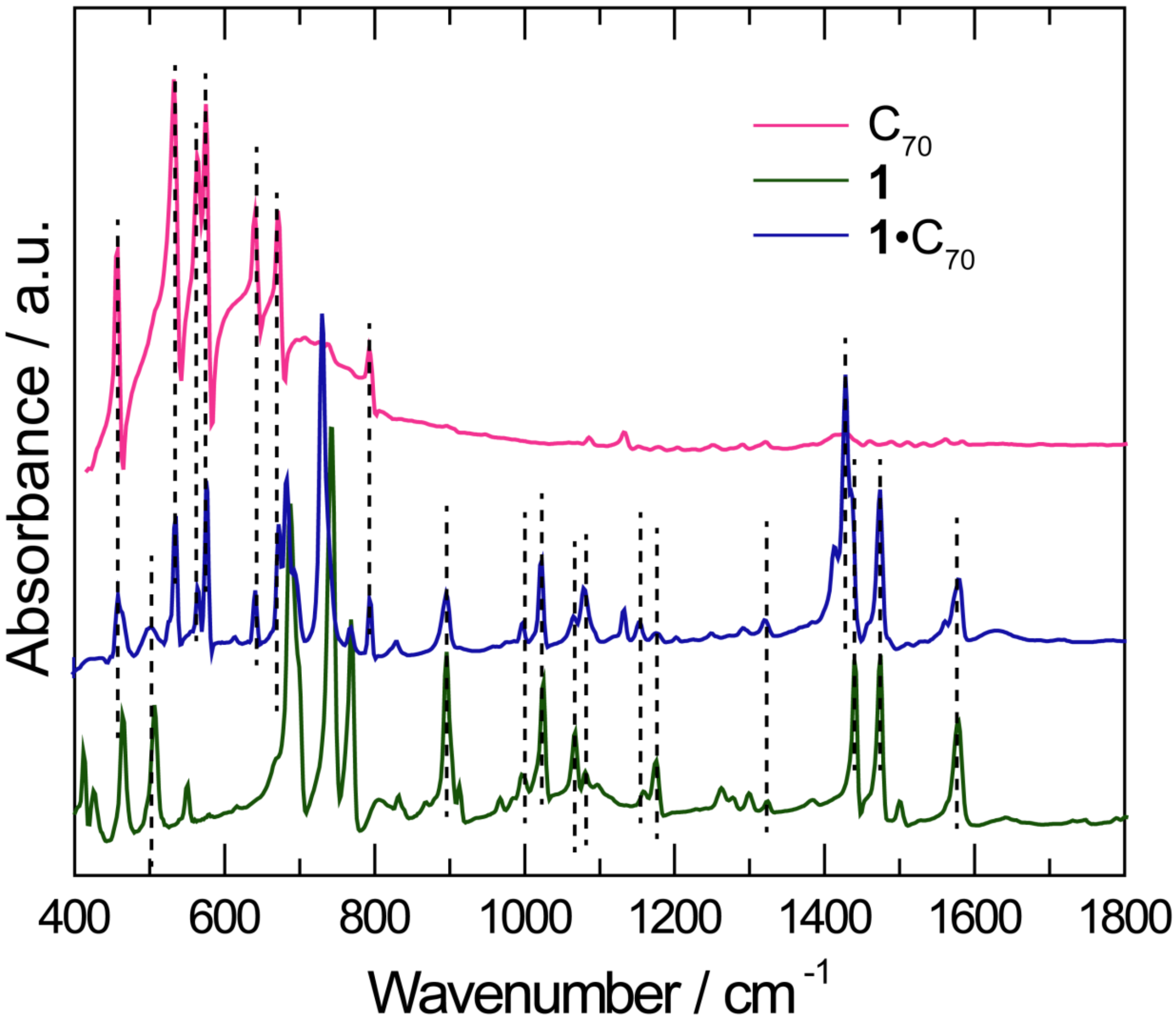

Figure S36: IR spectra of **1**•C_{70} along with those of **1** and C_{70} for comparison (in KBr). The black dashed lines are guides for the eye.

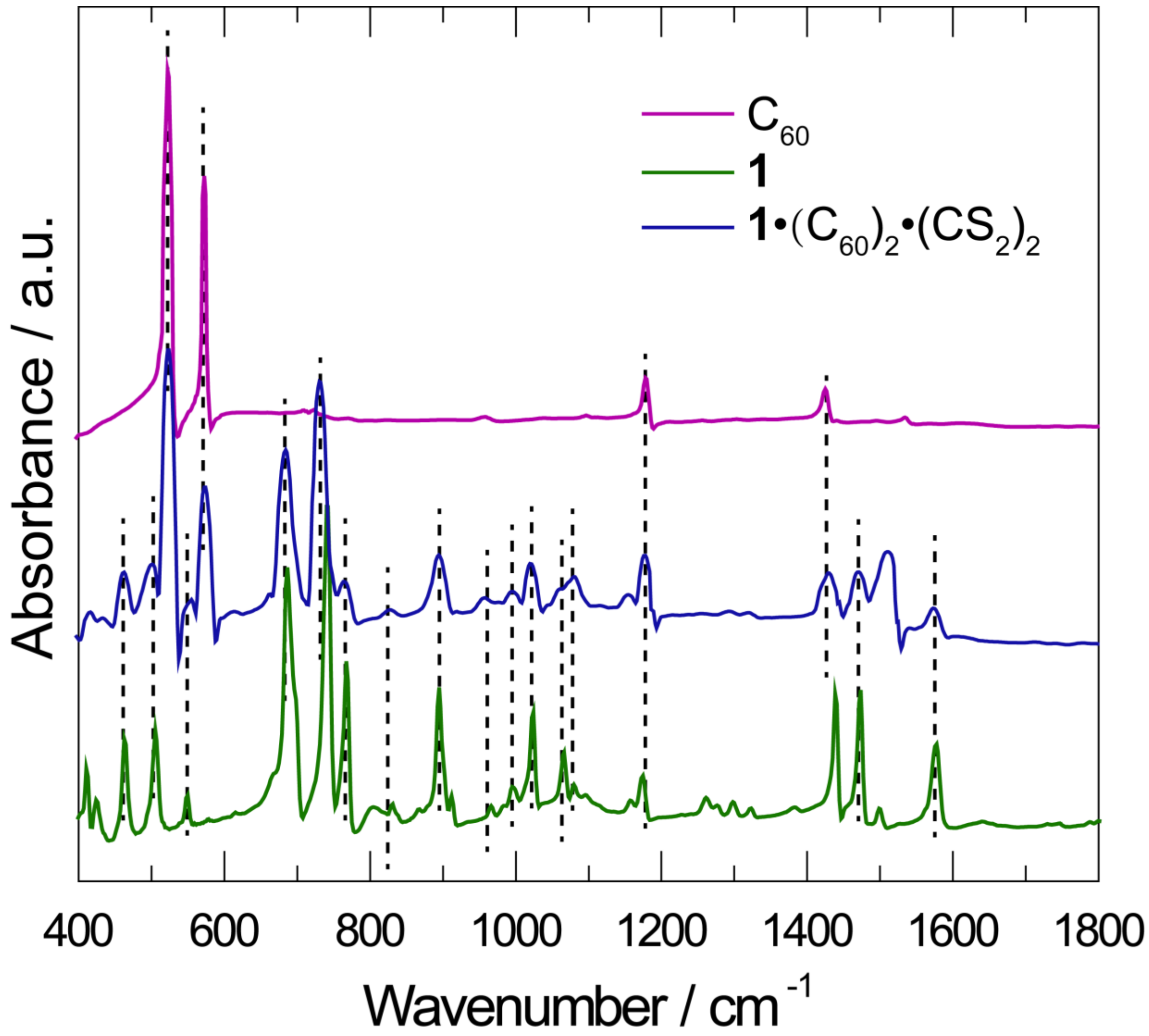

Figure S37: IR spectra of **1**•$(C_{60})_2$•$(CS_2)_2$ along with those of **1** and C_{60} for comparison (in KBr). The black dashed lines are guides for the eye.

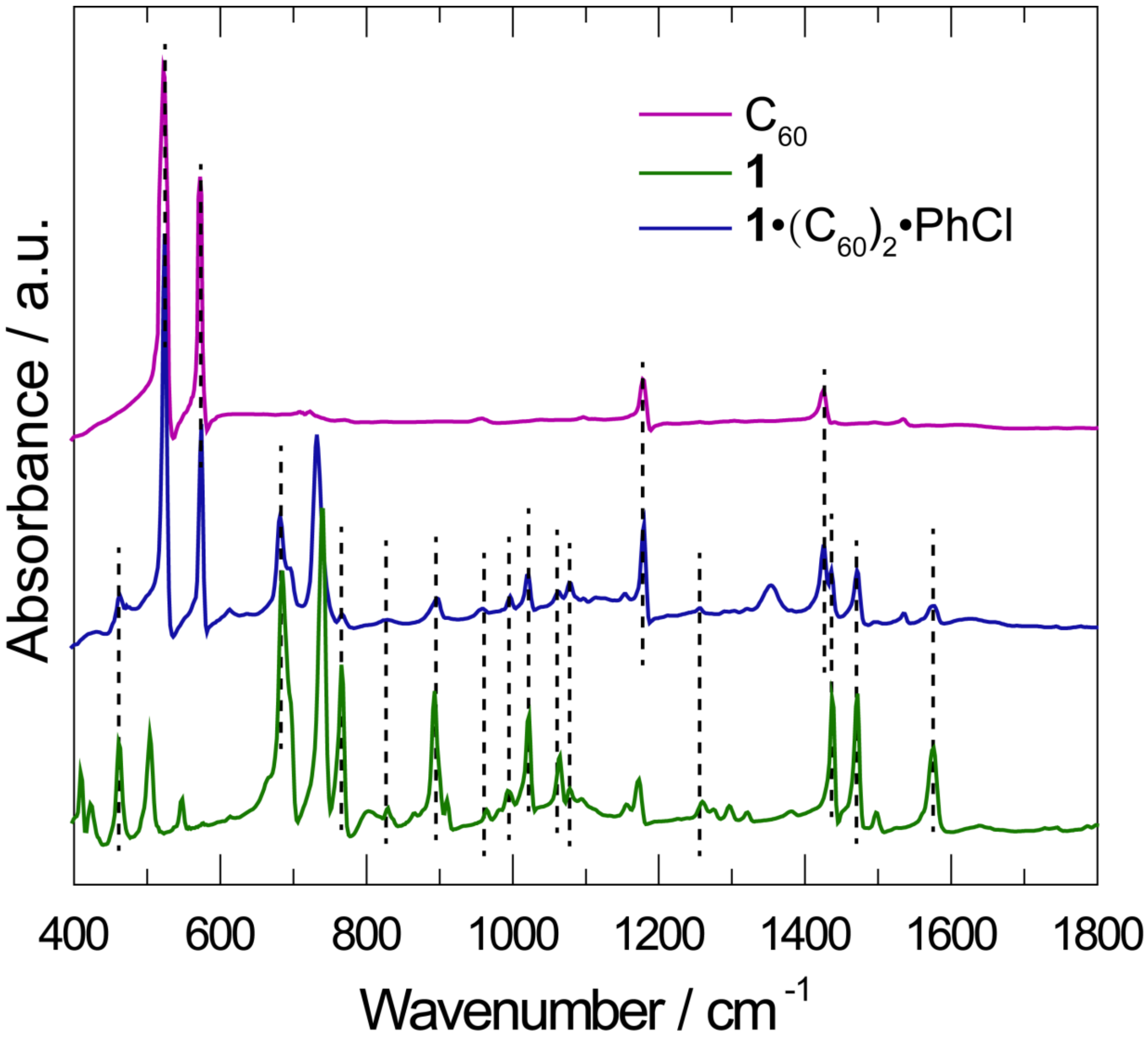

Figure S38: IR spectra of **1**•(C_{60})$_2$•PhCl along with those of **1** and C_{60} for comparison (in KBr). The black dashed lines are guides for the eye.

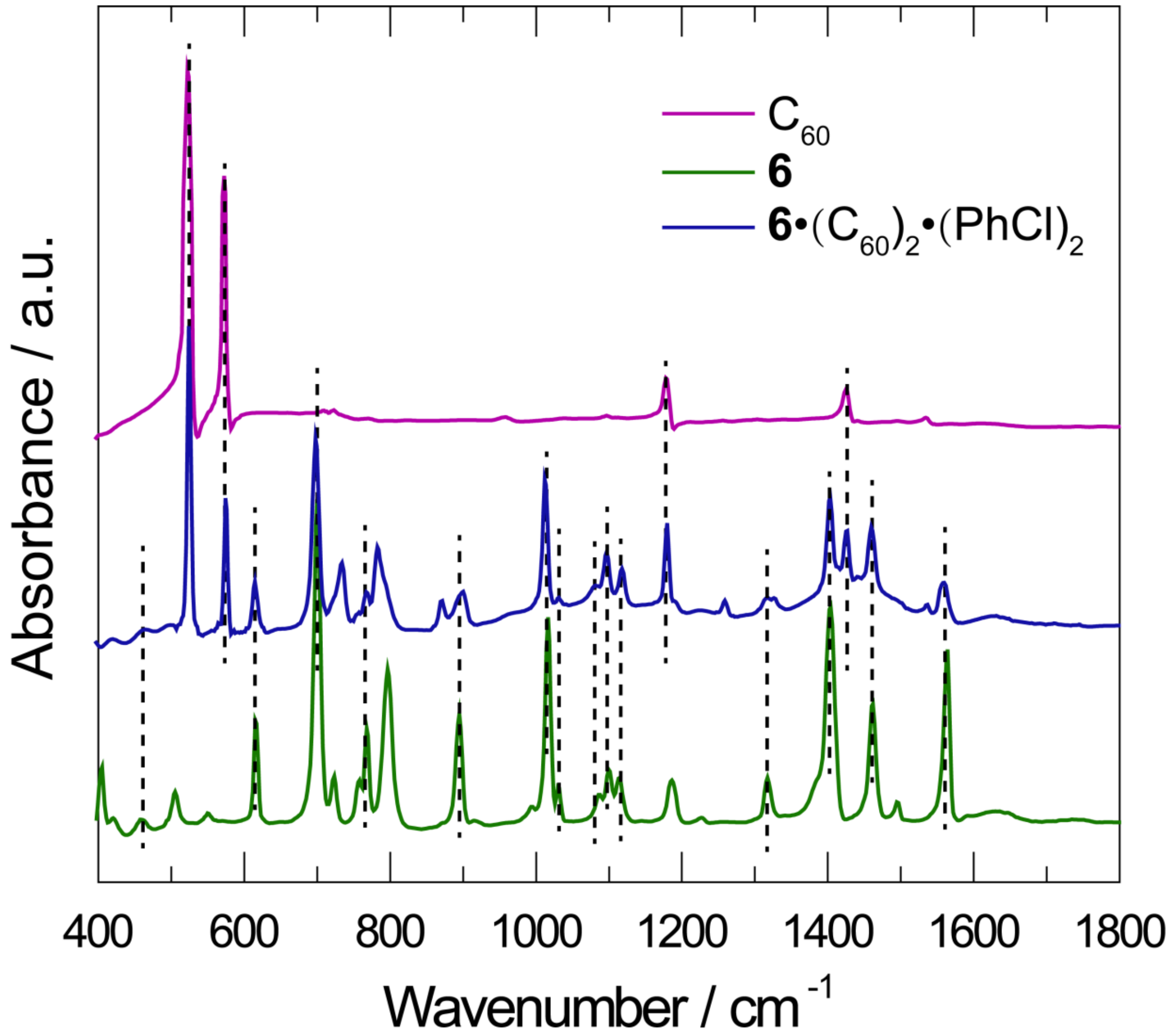

Figure S39: IR spectra of **6**•$(C_{60})_2$•$(PhCl)_2$ along with those of **6** and C_{60} for comparison (in KBr). The black dashed lines are guides for the eye.

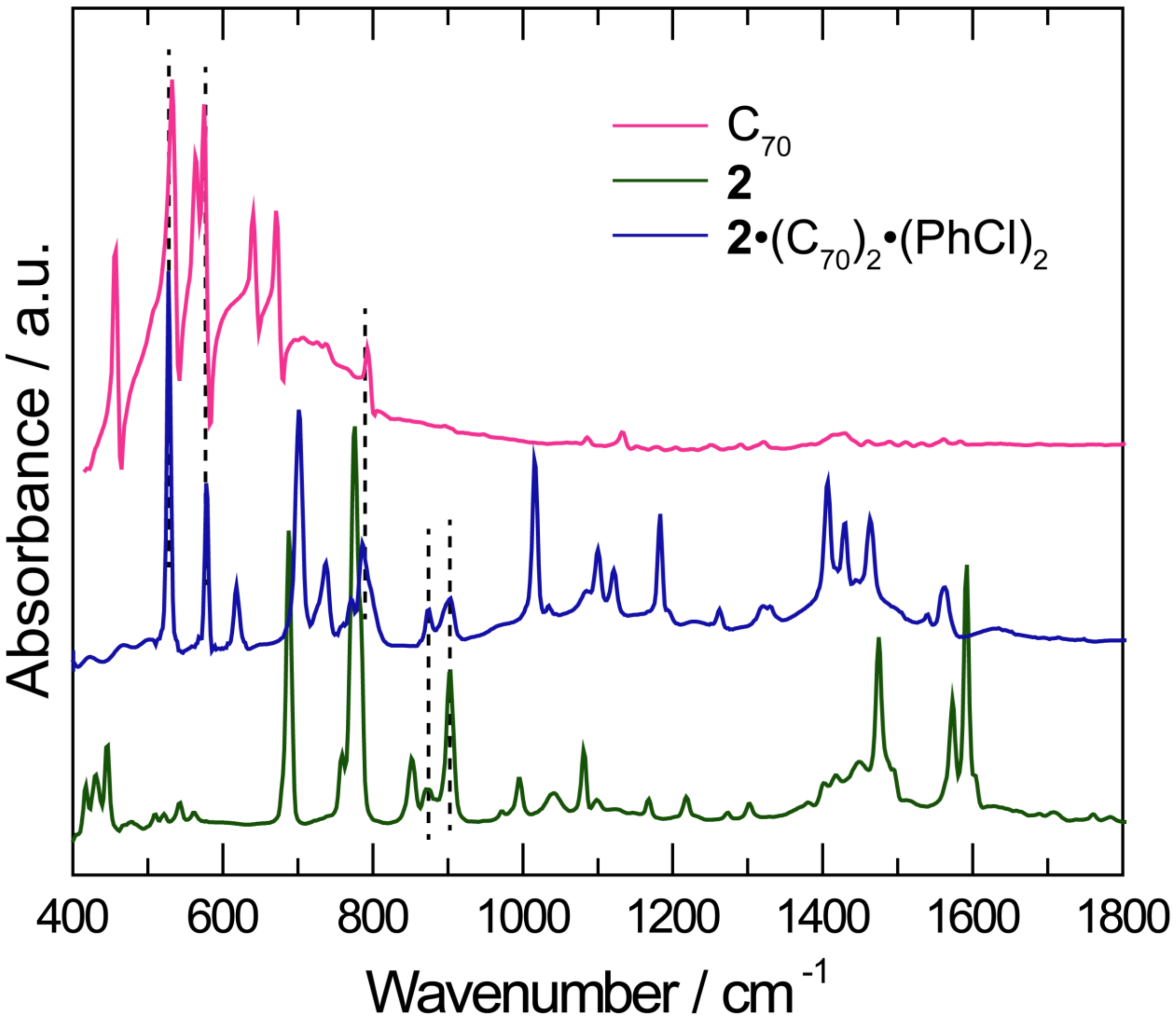

Figure S40: IR spectra of **2**•$(C_{70})_4$•$(PhCl)_2$ along with those of **2** and C_{70} for comparison (in KBr). The black dashed lines are guides for the eye.

X-ray powder diffraction (PXRD)

To insure the bulk samples having the same phase with the single crystals, the powder X-ray diffraction analysis (PXRD) of the co-crystals were performed. The observed PXRD patterns are almost identical to the corresponding PXRD calculated from the single crystal structure analysis, thus the bulky samples have the same phase as the corresponding single crystals. The subtle differences would be ascribable to the measurement temperatures: the PXRD measurement was performed at room temperature, whereas most of the single crystal diffraction was performed at temperature lower than 150 K.

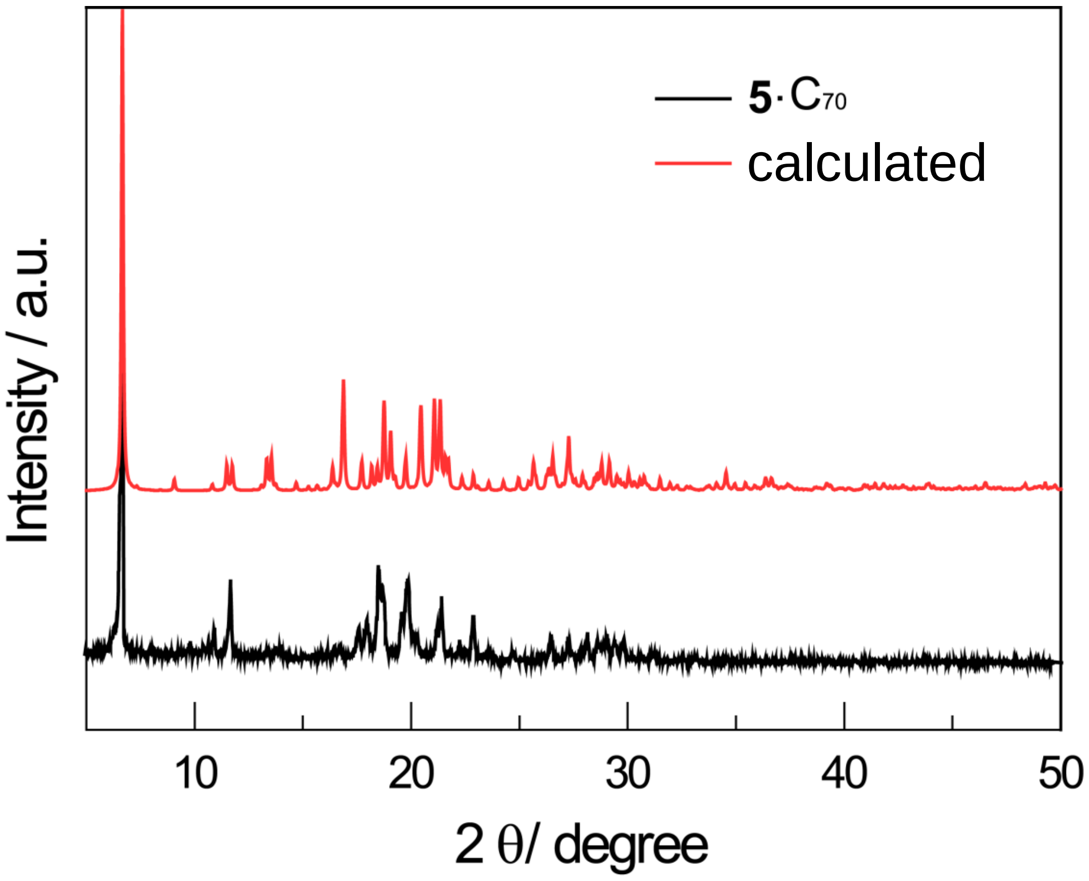

Figure S41: Experimental (black line) and calculated (red line) PXRD patterns for **5**•C_{70}.

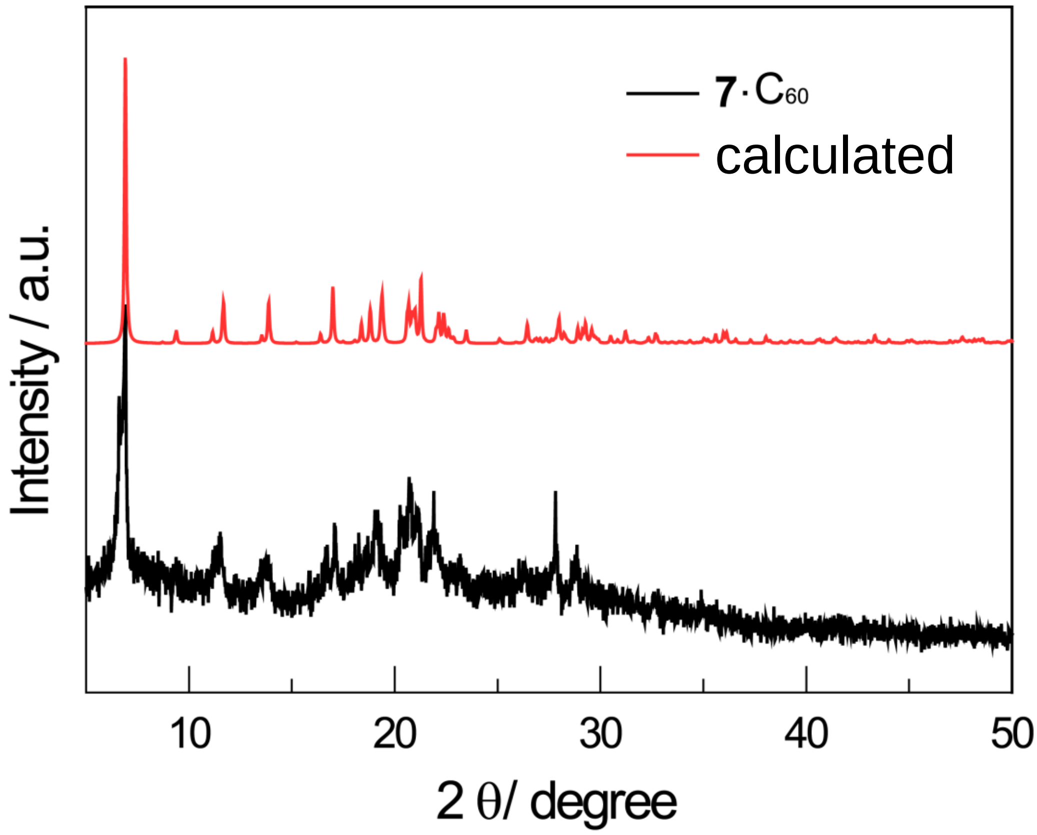

Figure S42: Experimental (black line) and calculated (red line) PXRD patterns for **7**•C_{60}.

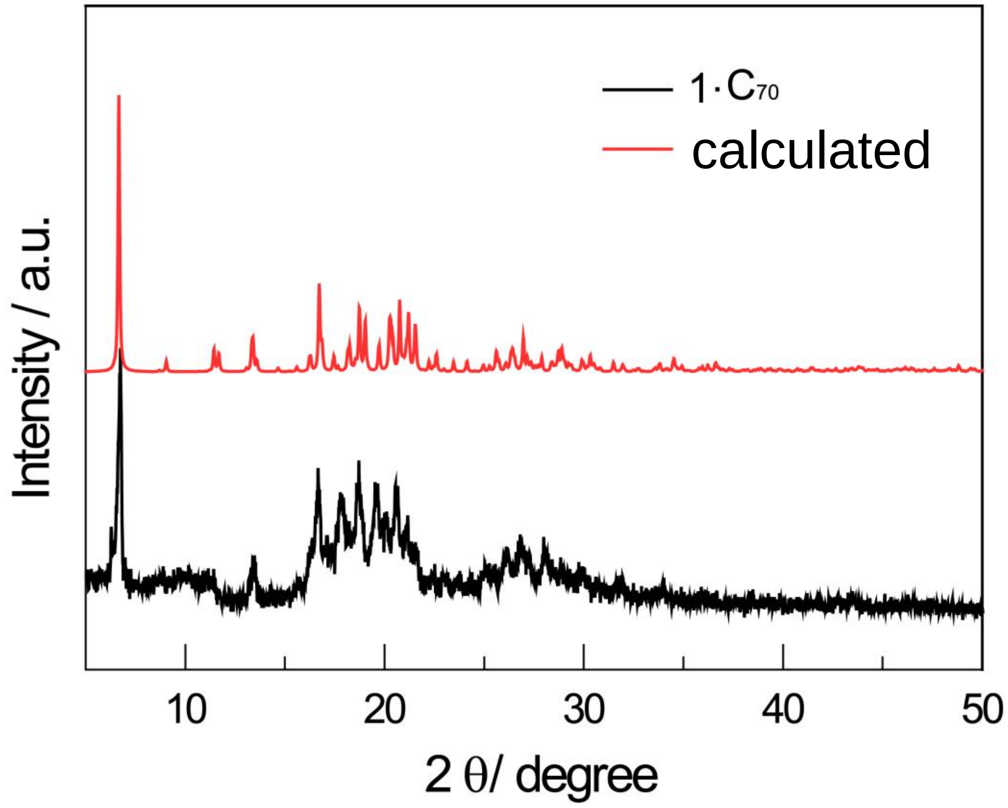

Figure S43: Experimental (black line) and calculated (red line) PXRD patterns for **1**•C_{70}.

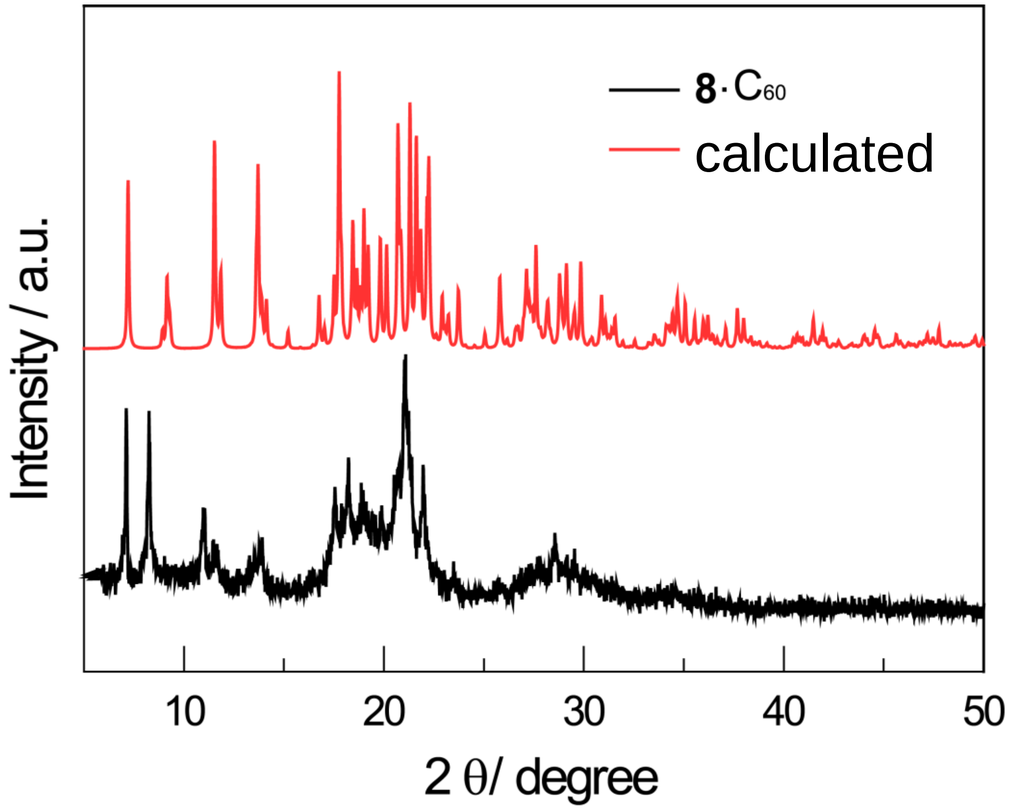

Figure S44: Experimental (black line) and calculated (red line) PXRD patterns for **8**•C_{60}.

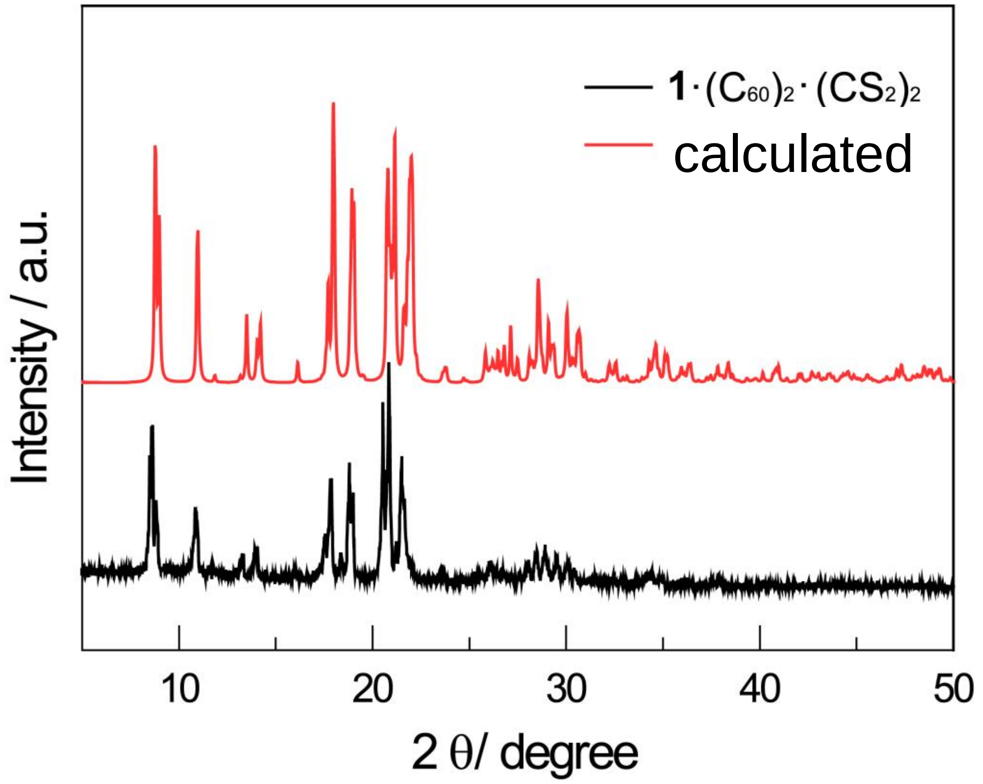

Figure S45: Experimental (black line) and calculated (red line) PXRD patterns for **1**•$(C_{60})_2$•$(CS_2)_2$.

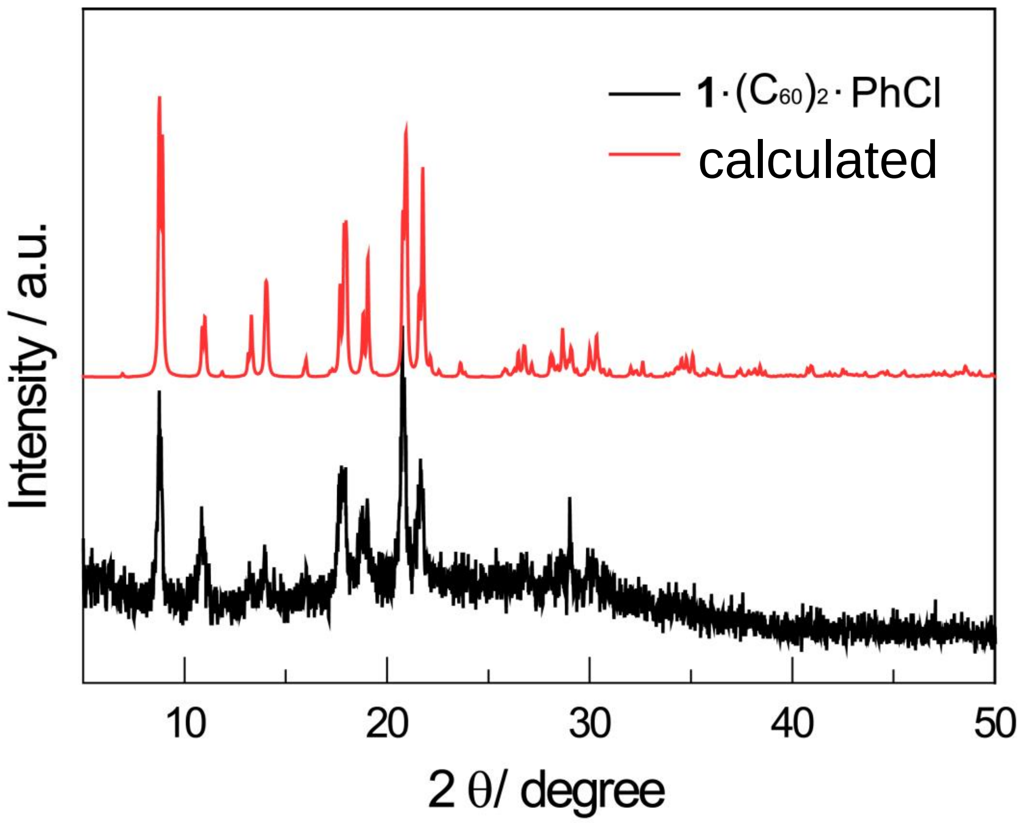

Figure S46: Experimental (black line) and calculated (red line) PXRD patterns for **1**•$(C_{60})_2$•PhCl.

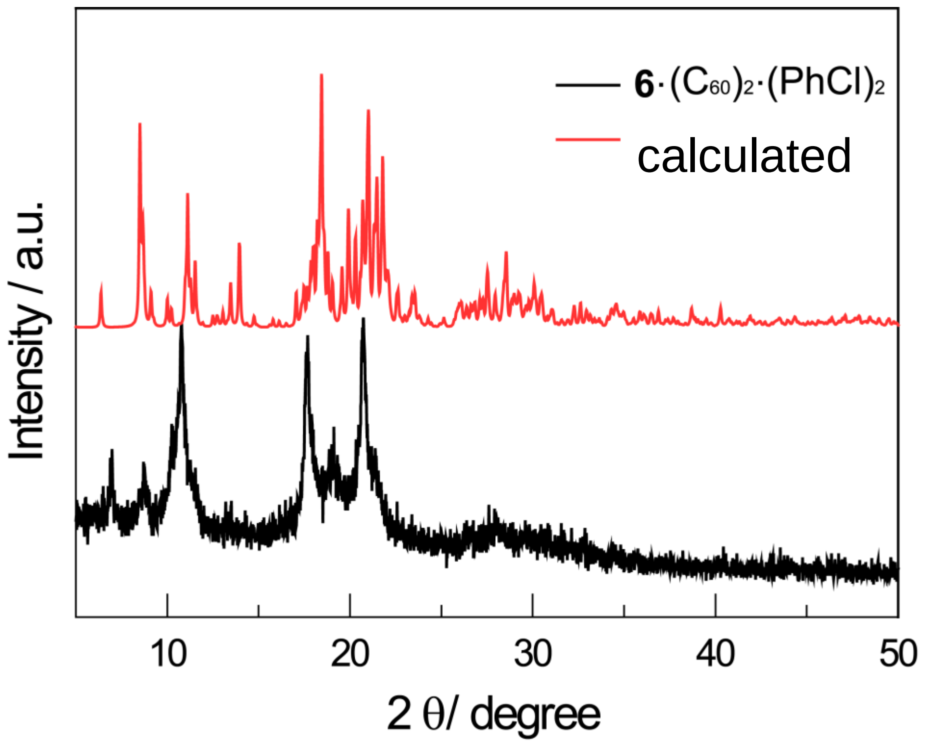

Figure S47: Experimental (black line) and calculated (red line) PXRD patterns for **6**•$(C_{60})_2$•$(PhCl)_2$.

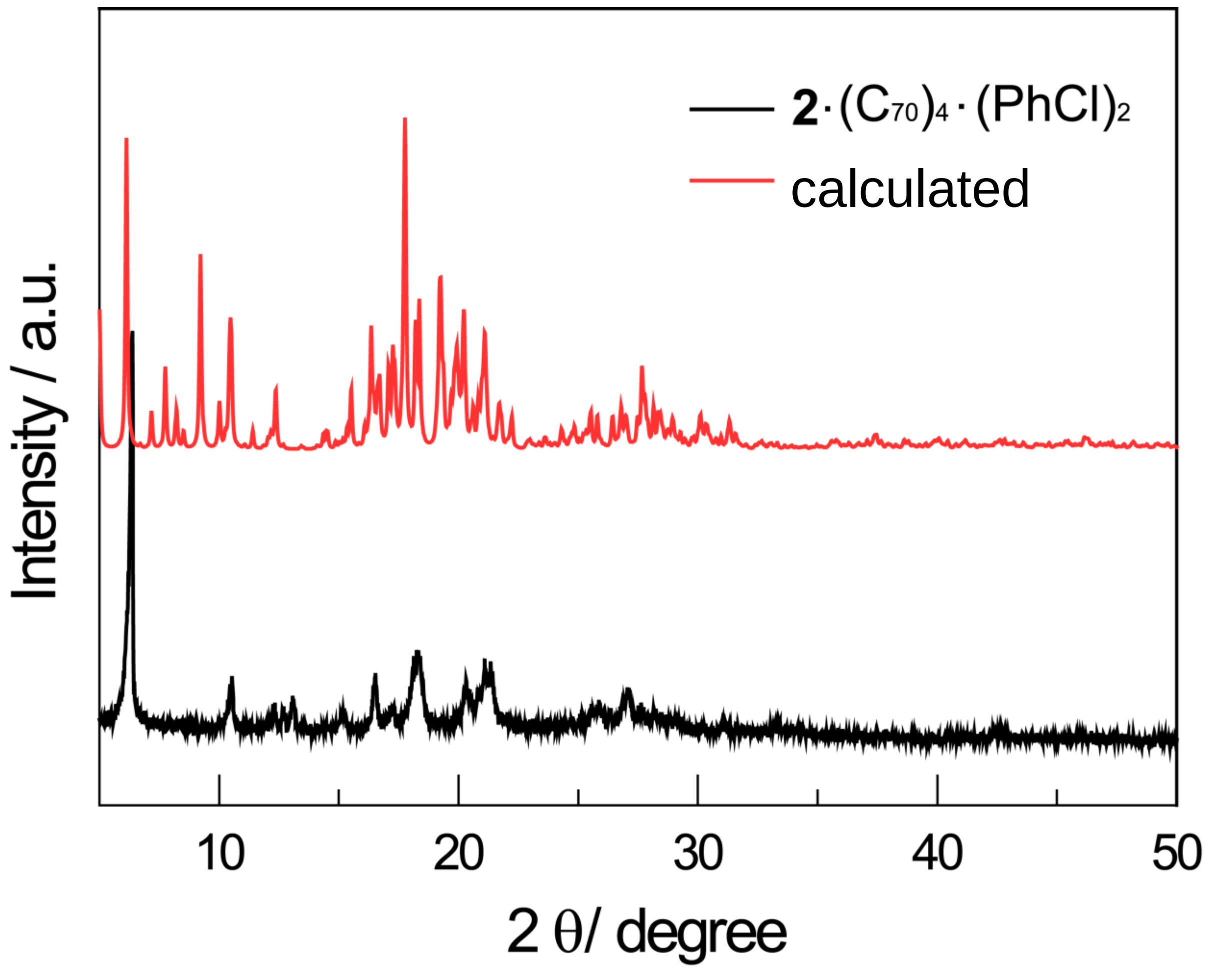

Figure S48: Experimental and calculated PXRD patterns for **2**•$(C_{60})_4$•$(PhCl)_2$.

Thermogravimetric analyses (TGA)

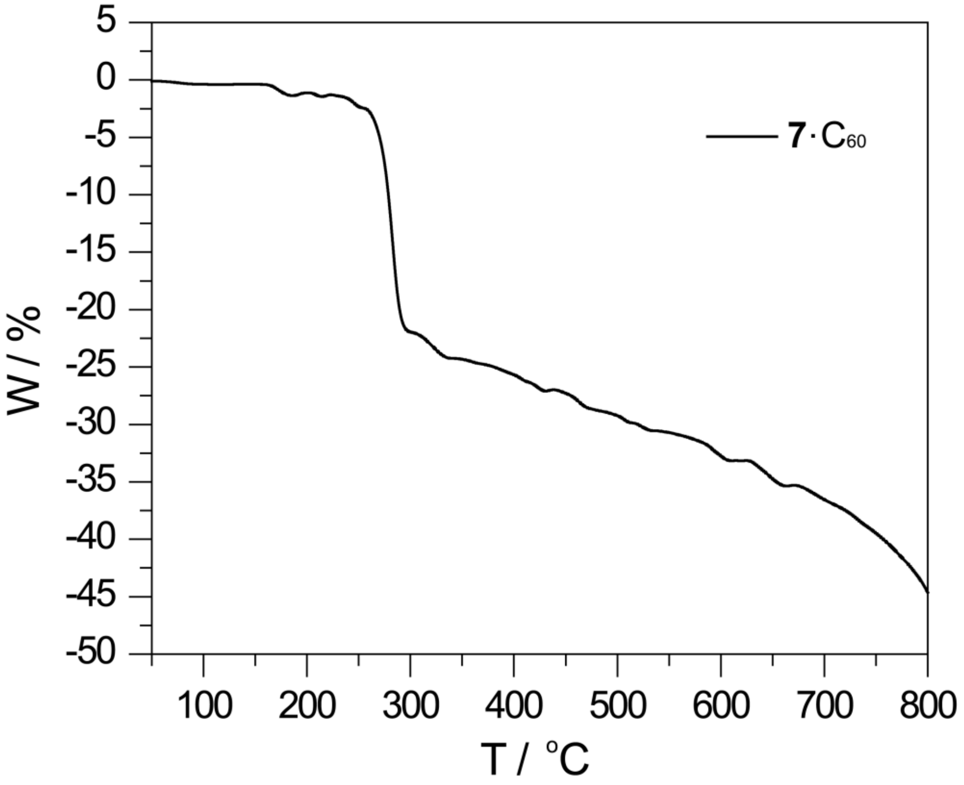

Figure S48: TGA graph of **7**•C_{60}.

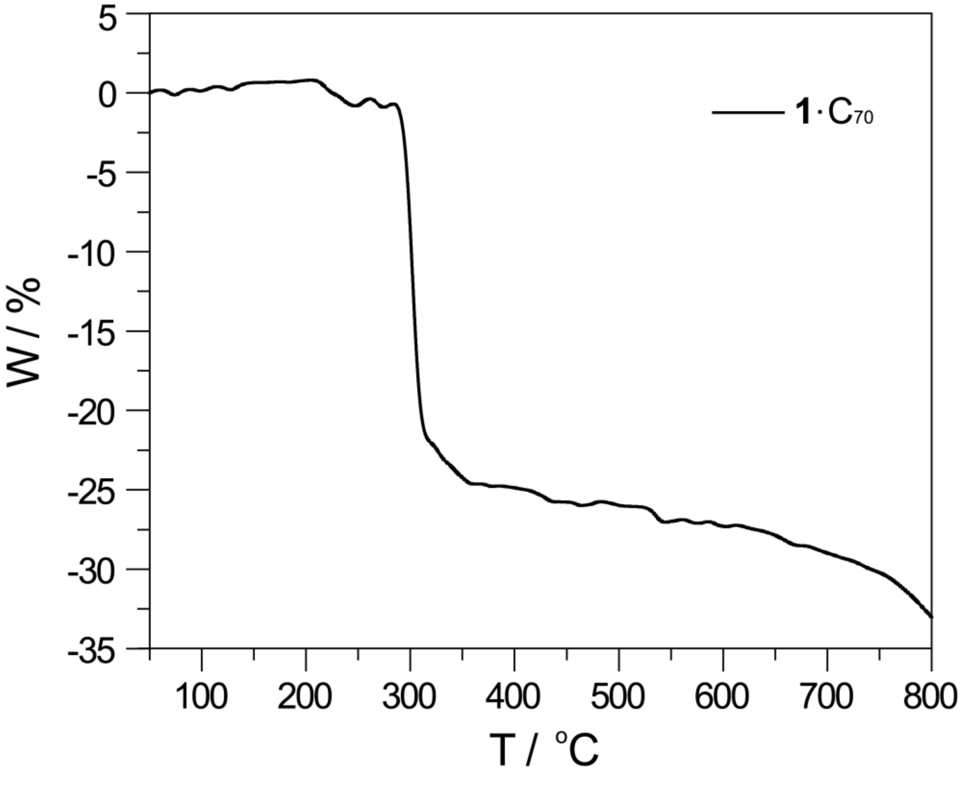

Figure S49: TGA graph of **1**•C_{60}.

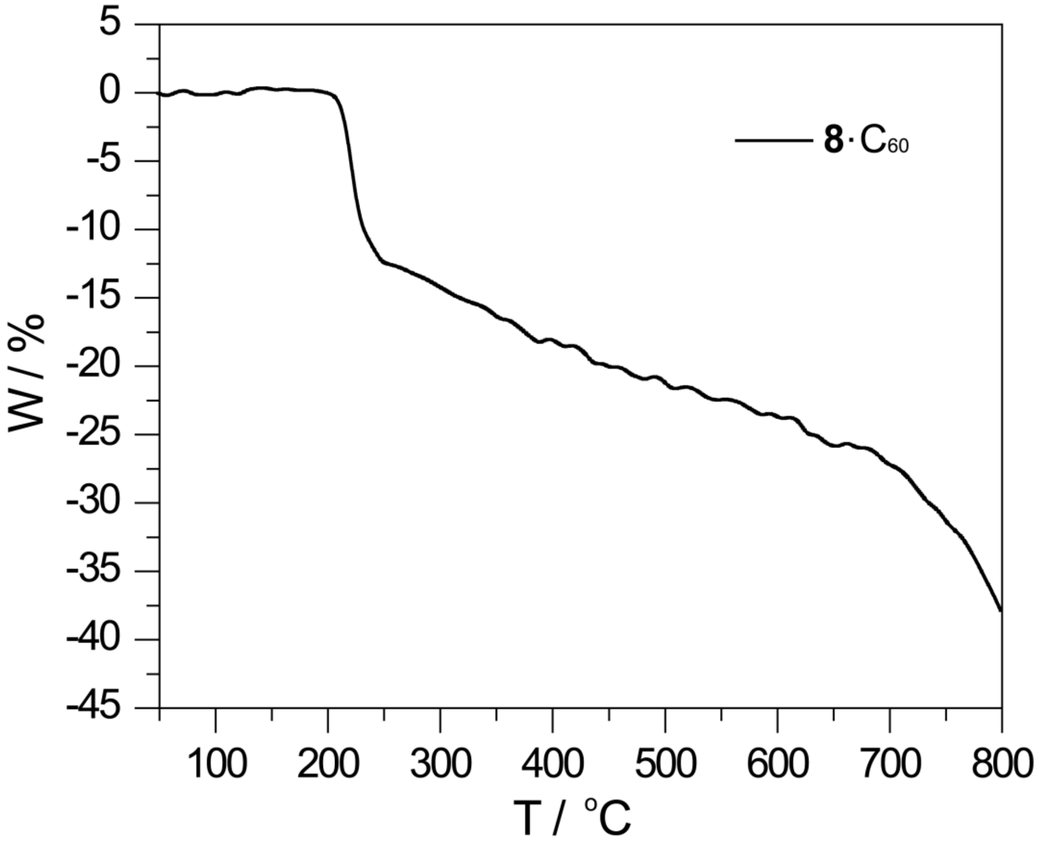

Figure S50: TGA graph of complex **8**•C_{60}.

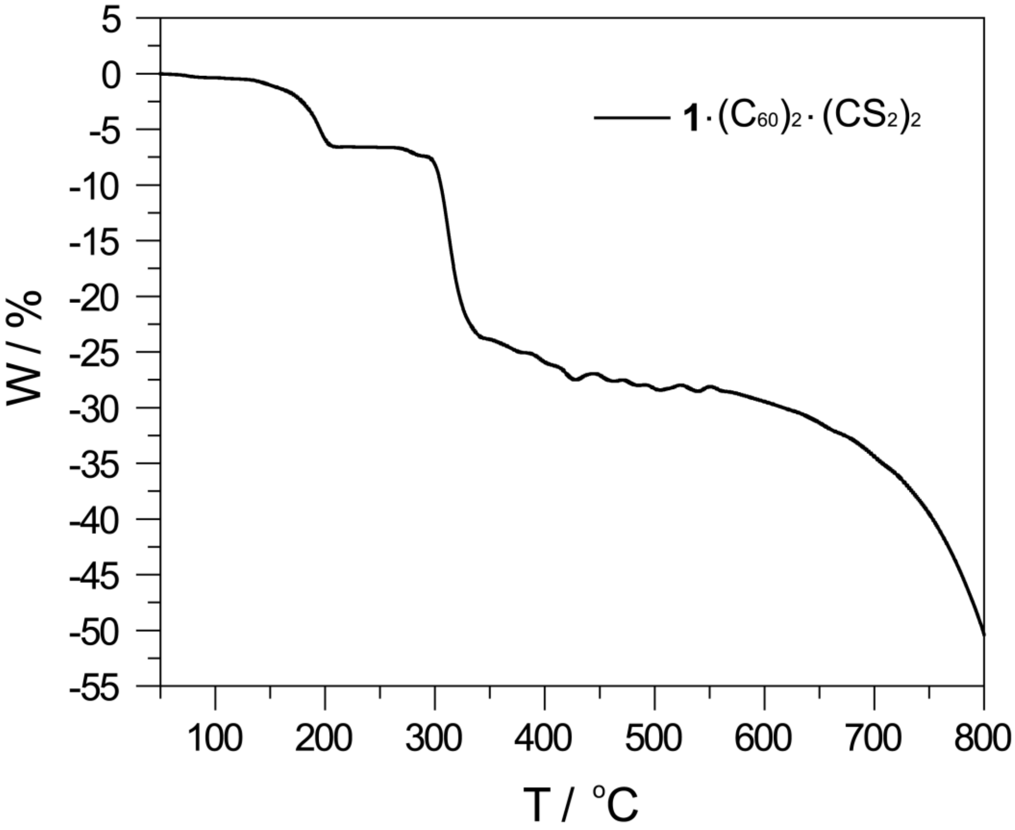

Figure S51: TGA graph of complex **1**•$(C_{60})_2$•$(CS_2)_2$.

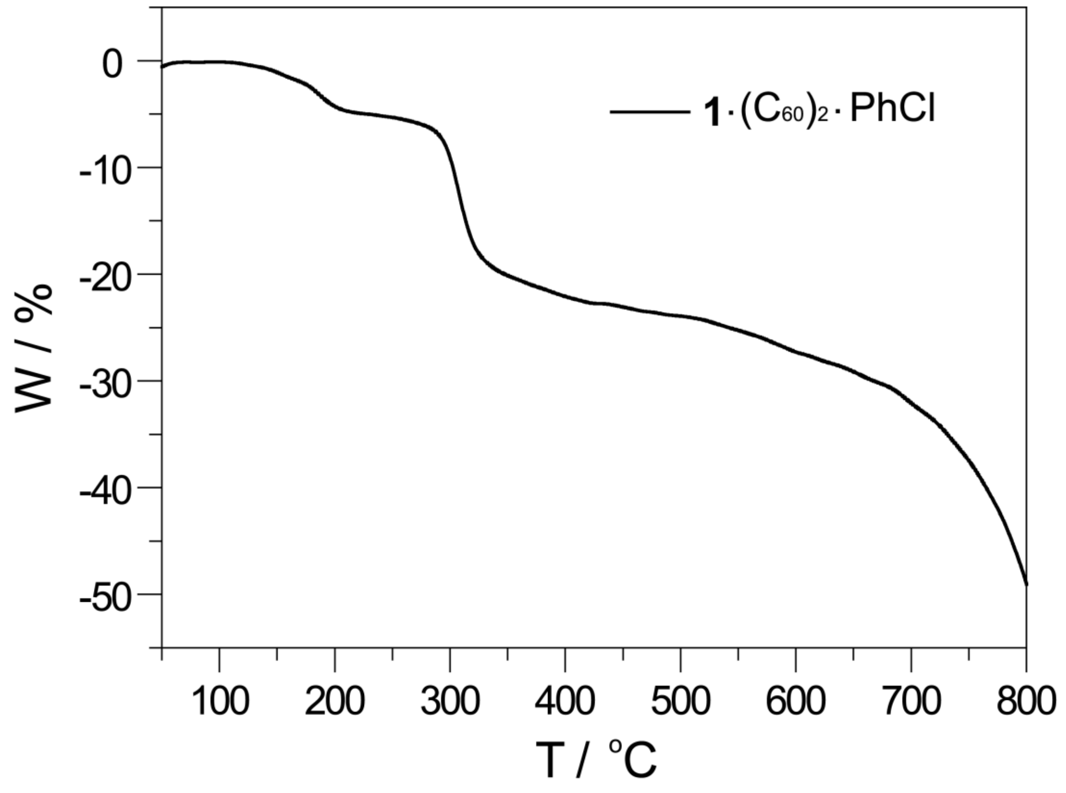

Figure S52: TGA graph of complex **1**•$(C_{60})_2$•PhCl.

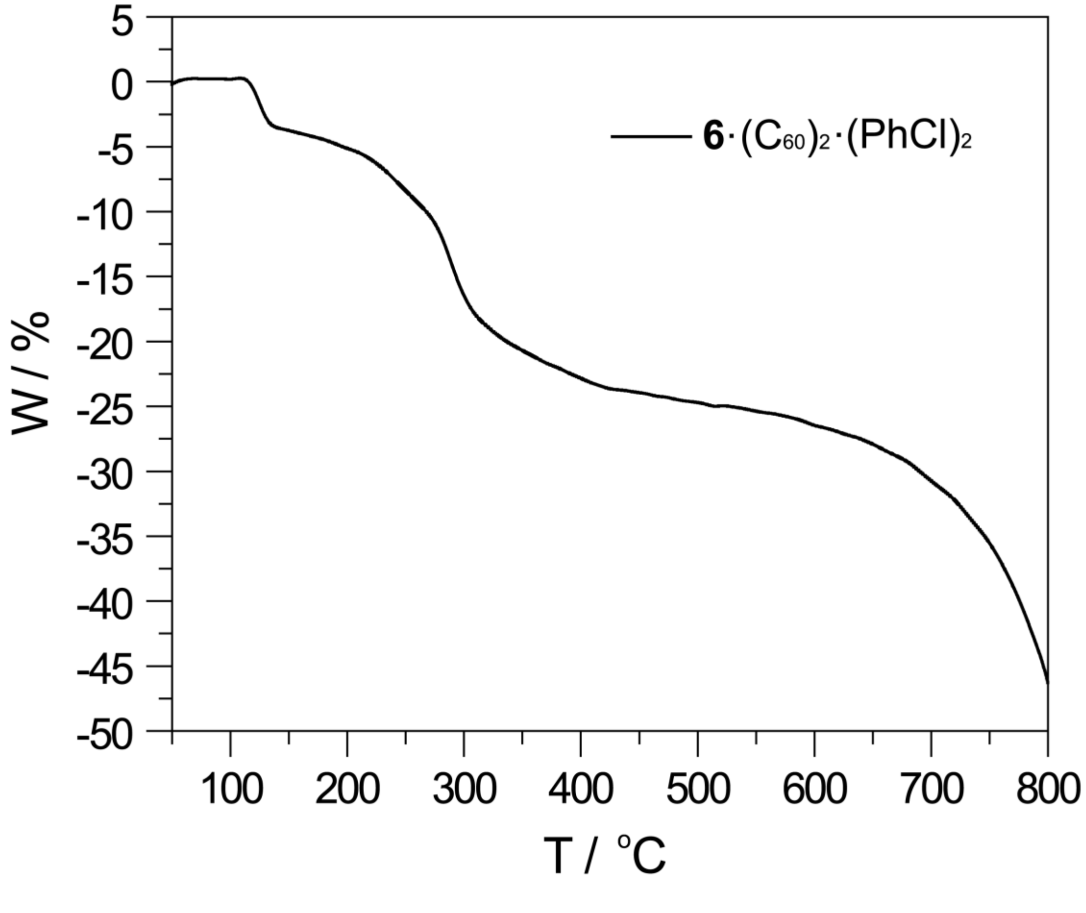

Figure S53: TGA graph of complex **6**•$(C_{60})_2$•$(PhCl)_2$.

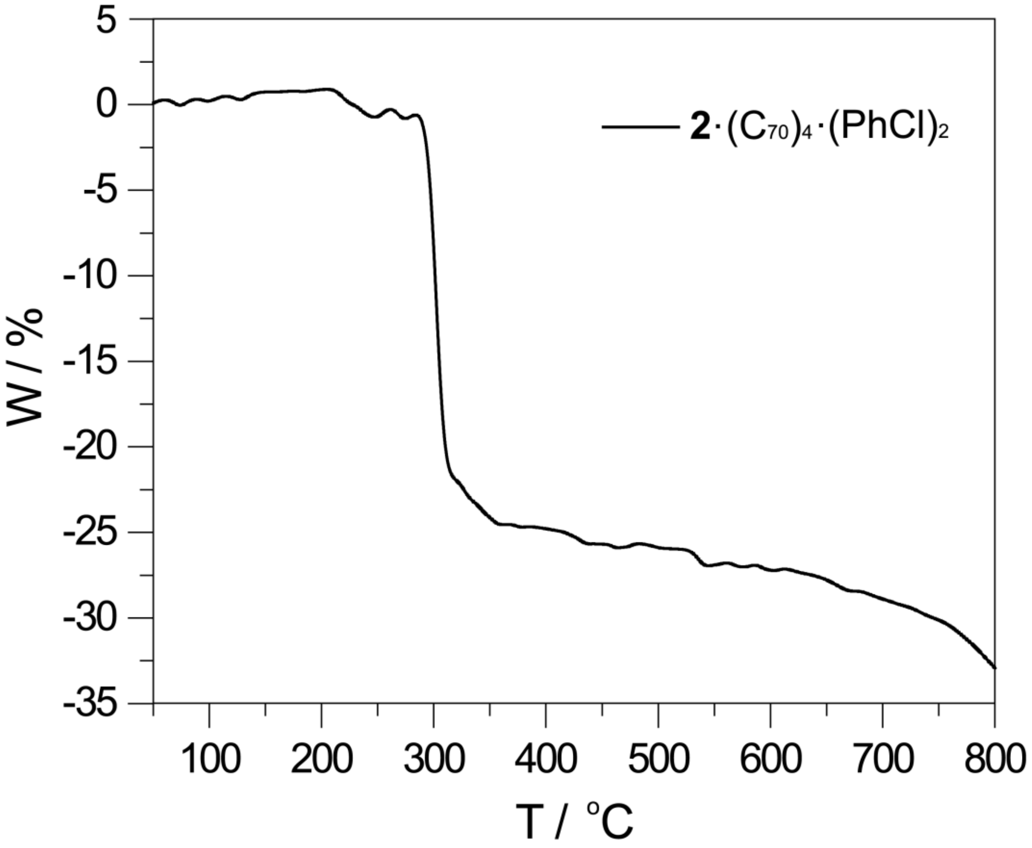

Figure S54: TGA graph of complex **2**•(C_{60})$_4$•(PhCl)$_2$.
